# The efficacy and safety of dachaihu decoction in the treatment of type 2 diabetes mellitus: A systematic review and meta-analysis

**DOI:** 10.3389/fphar.2022.918681

**Published:** 2022-08-08

**Authors:** Zehua Zhang, Yulin Leng, Xiaoxu Fu, Chan Yang, Hongyan Xie, Haipo Yuan, Qingzhi Liang, Hong Gao, Chunguang Xie

**Affiliations:** ^1^ Hospital of Chengdu University of Traditional Chinese Medicine, Chengdu, China; ^2^ TCM Regulating Metabolic Diseases Key Laboratory of Sichuan Province, Hospital of Chengdu University of Traditional Chinese Medicine, Chengdu, China; ^3^ West China Hospital of Sichuan University, Chengdu, China

**Keywords:** dachaihu decoction, type 2 diabetes mellitus, traditional Chinese medicine, systematic review, meta-analysis

## Abstract

**Background:** Type 2 diabetes mellitus (T2DM) is a clinical metabolic syndrome characterized by persistent hyperglycemia, which is caused by defective insulin secretion and decreased function in regulating glucose metabolism. Dachaihu Decoction (DCHD) is a traditional Chinese medicine formula that has been gradually used in T2DM treatment. A comprehensive analysis on the efficacy and safety of DCHD in T2DM treatment is necessary.

**Objective:** This meta-analysis aimed to systematically assess the clinical efficacy and safety of DCHD in the T2DM treatment and provide a reference for subsequent research and clinical practice.

**Methods:** Both Chinese and English databases were searched from their inceptions to November 2021. All retrieved studies were screened according to inclusion and exclusion criteria and randomized controlled trials about DCHD on T2DM were enrolled. The quality of the literature was assessed using the bias risk assessment tool in the Cochrane Handbook. Data extraction was performed on the selected studies. Review Manager 5.4 and Stata 16.0 were used for meta-analysis. Sources of heterogeneity were also explored by using meta-regression and subgroup analysis. Funnel plot and Egger’s test were used to assess publication bias and the evidence quality was assessed by GRADE.

**Results:** 17 eligible studies, involving 1,525 patients, were included in this study. Compared with conventional treatment, combined treatment with DCHD was significantly better in improving HbA1c (MD = −0.90%, 95%CI: −1.20 to −0.60, *p* < 0.01), FBG (MD = −1.08 mmol/L, 95%CI: −1.28 to −0.87, *p* < 0.01), 2hPG (MD = −1.25 mmol/L, 95%CI: −1.42 to −1.09, *p* < 0.01), TC (MD = −0.50 mmol/L, 95%CI: −0.70 to −0.30, *p* < 0.01), TG (MD = −0.44 mmol/L, 95%CI: −0.61 to −0.26, *p* < 0.01), LDL-C (MD = −0.58 mmol/L, 95%CI: −0.85 to −0.31, *p* < 0.01), HOMA-IR (SMD = −2.04, 95%CI: −3.09 to −0.99, *p* < 0.01), HOMA-β (SMD = 2.48, 95%CI: 2.20 to 2.76, *p* < 0.01) and BMI (MD = −1.52 kg/m^2^, 95%CI: −2.55 to −0.49, *p* < 0.01). When DCHD used alone, it had a similar efficacy to conventional treatment in HbA1c (MD = −0.04%, 95%CI: −0.17 to 0.09, *p* = 0.57) and FBG (MD = 0.13 mmol/L, 95%CI: −0.09 to 0.36, *p* = 0.24). It can also reduce 2hPG, even if not as effective as conventional treatment (MD = 0.54 mmol/L, 95%CI: 0.19 to 0.89, *p* < 0.01). Due to the small number of included studies, it is unclear whether DCHD used alone has an improving effect on lipid metabolism, BMI, HOMA-IR and HOMA-β. Analysis of adverse events showed DCHD was relatively safe. No obvious publication bias was detected by Funnel plot and Egger’s test.

**Conclusion:** Based on this meta-analysis, we found that the combination with DCHD in the T2DM treatment has more advantages than conventional treatment alone, which can further regulate the glucose and lipid metabolism, reduce insulin resistance, improve islet function and lower BMI. DCHD alone also plays a certain role in regulating glucose. Meanwhile, DCHD is relatively safe. However, limited by the quality and quantity of included studies, the efficacy and safety of DCHD remain uncertain. More high-quality studies are still needed to provide more reliable evidence for the clinical application of DCHD.

**Systematic Review Registration:** https://www.crd.york.ac.uk/prospero/display_record.php?ID=CRD42021296718, identifier CRD42021296718.

## Introduction

Type 2 diabetes mellitus (T2DM) is a clinical metabolic syndrome characterized by persistent hyperglycemia, which is caused by multiple factors such as heredity, environment and immunity. The intrinsic pathological mechanism on T2DM is an impairment in the metabolism and utilization of glucose and lipid due to insulin resistance or insufficient insulin secretion (Roden and Shulman, 2019; Reed et al., 2021). As the disease progresses, it causes damage to target organs such as the kidney, heart, blood vessels and nerves, resulting in dysfunction or failure of tissues and organs and even disability or death (Singh et al., 2022). With the improvement of living standards and changes in diet structure, the prevalence of T2DM has shown an obvious upward trend (Ma, 2018; Lin et al., 2020; Wang et al., 2021). In 2021, there were about 573 million adults with diabetes worldwide, representing approximately 10.5% of the world’s population, of whom about 90% had T2DM (IDF, 2021). China has the largest number of diabetics, with around 140 million people suffering from diabetes. T2DM and its complications have become one of the leading causes of death worldwide (Stanaway et al., 2018; Glovaci et al., 2019; Martinez et al., 2020; WHO, 2020). It not only seriously affects the life quality of patients but also brings huge medical and economic burdens to individuals and society (WHO, 2016; Cannon et al., 2018; Zheng et al., 2018). How to effectively prevent the occurrence and development of T2DM and its complications has always been an important public health issue (Li et al., 2020; Ling et al., 2020; Liu et al., 2020).

The current treatment for T2DM is based on a combination of blood glucose regulation, blood pressure control, lipid lowering, microcirculation improvement and lifestyle intervention (ADA, 2021). However, the existing hypoglycemic drugs have the potential to cause gastrointestinal adverse effects, reduce vitamin B12 concentrations and induce urinary tract infection (Baye et al., 2021). At the same time, even if blood glucose is well controlled, the existence of metabolic memory still makes it difficult to effectively prevent the emergence and progression of T2DM and its complications (Bianchi et al., 2013; Zhang and Wu, 2014; Galicia-Garcia et al., 2020). Therefore, there is an urgent need to find safer and more effective treatments.

In recent years, traditional Chinese medicine (TCM) has gradually shown its unique advantages in treating T2DM. Many studies have shown that TCM treatment can significantly improve the clinical symptoms and life quality of T2DM patients, reduce insulin resistance, decrease the occurrence of adverse effects and consolidate clinical efficacy (Ji et al., 2013; Tong et al., 2013; Lian et al., 2015; Zhang Y. et al., 2019; Pang et al., 2021). Dachaihu Decoction (DCHD) is one of the classical formulas in ancient China. It comes from the *Treatise on Cold Damage and Miscellaneous Diseases* (*Shang Han Za Bing Lun*) by *Zhongjing Zhang*, a famous doctor in the *Eastern Han Dynasty*. It is composed of eight herbs: Chinese Thorowax Root (*Chaihu*, Bupleurum falcatum L.), Baical Skullcap Root (*Huangqin*, Scutellaria baicalensis Georgi), Rhubarb (*Dahuang*, Rheum palmatum L.), Immature Orange Fruit (*Zhishi*, Citrus aurantium L.), Pinellia Tuber (*Banxia*, Pinellia ternata (Thunb.) Makino), White Paeony Root (*Baishao*, Paeonia lactiflora Pall.), Chinese Date (*Dazao*, Ziziphus jujuba Mill.) and Fresh Ginger (*Shengjiang*, Zingiber officinale Roscoe). It has the functions of soothing liver and relieving depression, clearing stomach and purging heat, and is mainly used to treat the syndrome of heat stagnation in liver and stomach. The main active components of DCHD measured by high performance liquid chromatography include paeoniflorin, naringin, hesperidin, neohesperidin, baicalin, baicalein and saikosaponin A (Li et al., 2006; Liu, 2014). Many studies have shown that DCHD has the effect of anti-inflammatory, regulating bile acid metabolism, balancing intestinal flora, protecting liver function and modulating blood lipids (Yoshie et al., 2004; Feng et al., 2019; Yang et al., 2019; Cui H. et al., 2020; Yang et al., 2021; Wang et al., 2022). It can be used in treating diseases such as cholecystitis, acute pancreatitis, bile reflux gastritis, fatty liver and hyperlipidemia (Xu and Sun, 2013; Zou and Wang, 2014; Qian et al., 2016; Dou et al., 2019; Han et al., 2020; Yang et al., 2021). In recent years, DCHD has also been gradually used to treat T2DM. The indications of DCHD for T2DM patients include thirst, bitter taste in mouth, fever, impatience and irascibility, hypochondriac pain, epigastric burning pain, increased eating with rapid hungering, red tongue with yellow coating and wiry or rapid pulse (CDS, 2021). Studies ranging from case reports, retrospective studies to randomized controlled trials (RCTs) suggest that DCHD or its modified may be able to relieve clinical symptoms related to T2DM, improve glucose and lipid metabolism and reduce insulin resistance (Zhang et al., 2015; Zhang, 2016; Li and Yan, 2019; Deng and Huang, 2020; Zou et al., 2021). However, the clinical efficacy of DCHD in T2DM remains uncertain due to limited sample size, inconsistent trial designs, different efficacy indicators and ambiguous methodological quality. Moreover, there is no clinical evidence summarizing the efficacy and safety of DCHD in the T2DM treatment. Therefore, this study comprehensively collected RCTs of DCHD alone or in combination with hypoglycemic drugs in the T2DM treatment and evaluated the clinical efficacy and safety of DCHD, in order to provide a reference for subsequent research and clinical practice.

## Materials and methods

### Study registration

This systematic review and meta-analysis was conducted and reported under the guidance of the Cochrane Handbook for Systematic Reviews of Interventions version 6.3 (updated 2022) and the Preferred Reporting Items for Systematic Review and Meta-Analysis (PRISMA) 2020 Statement (Moher et al., 2009; Page et al., 2021). The PRISMA 2020 checklist is provided in Supplementary Material S1. Before starting, this study was registered in the International Prospective Register of Systematic Reviews (PROSPERO) (Registration number: CRD42021296718). Data were derived from published clinical studies.

### Database and search strategies

We conducted a comprehensive search of three English electronic databases, namely PubMed, EMBASE and the Cochrane Library, and three Chinese electronic databases, including the China National Knowledge Infrastructure (CNKI), Wan Fang Database, and China Science and Technology Journal Database (VIP), from their inceptions to November 2021. The clinical trials related to DCHD, modified DCHD, T2DM were searched using a combination of subject terms and text words. The search terms mainly included: “Dachaihu,” “Dachaihu Decoction,” “Dachaihu Tang,” “Da Chaihu,” “Da Chaihu Tang,” “Da Chaihu Decoction,” “Major Bupleurum Decoction,” “Major Bupleurum Tang,” “Daisaikoto,” “Type 2 Diabetes Mellitus,” “Diabetes Mellitus, Type 2,” “Type 2 Diabetes,” “Diabetes, Type 2” and “Noninsulin-Dependent Diabetes Mellitus”. The detailed search strategies containing more search terms are provided in Supplementary Material S2. To understand ongoing studies, the ClinicalTrials.gov database and Chinese Clinical Trial Registry (CHiCTR) were also retrieved. Additionally, references of related reviews and meta-analyses were also screened to discover literature that may be missed in online searches. Only original articles in English and Chinese were included. All literature was selected according to inclusion criteria and exclusion criteria.

### Inclusion criteria

#### Type of studies

RCTs were included without restriction on origins or countries, for they were considered to have high-quality evidence in assessing the effects of interventions. The publication language was limited to English or Chinese.

#### Type of participants

Adults (at least 18 years old) diagnosed with T2DM were included regardless of complications. Patients had no demographic restrictions such as age, race or gender.

#### Type of interventions

Interventions involving DCHD or modified DCHD were included, without limit to dosage form (decoction, capsule or granules), frequency or dosage. The experimental group can be DCHD alone or DCHD combined with conventional treatment. The control group can be placebo or conventional treatment. Conventional treatment refers to the classic treatment measures of western medicine, including diabetes health education, diet management, exercise intervention, blood glucose monitoring and hypoglycemic drugs. There is no restriction on the type or dosage form (oral preparation or injection) of hypoglycemic drugs. If the experimental group was combined with conventional treatment, it should be the same as the control group.

#### Type of comparisons

The following comparisons were made respectively in this study:

DCHD combined with conventional treatment vs. conventional treatment
DCHD vs. conventional treatment
DCHD vs. placebo

#### Type of outcome measures

To comprehensively evaluate the efficacy and safety of DCHD in patients with T2DM, the analysis was made from the perspectives of glucose metabolism, lipid metabolism, insulin resistance, pancreatic islet function, body mass index (BMI) and adverse events. RCTs evaluating any of the following outcomes were included:

1) Primary Outcomes
  Glucose metabolism index: Glycated hemoglobin (HbA1c), Fasting blood glucose (FBG), 2-h postprandial glucose (2hPG)
2) Secondary Outcomes
  Lipid metabolism index: Total cholesterol (TC), Triglyceride (TG), High-density lipoprotein cholesterol (HDL-C), Low-density lipoprotein cholesterol (LDL-C)
  Insulin resistance index: Homeostasis model assessment of insulin resistance (HOMA-IR)
  Pancreatic islet function index: Homeostasis model assessment of beta-cell function (HOMA-β)
  Body mass index
  If a study reported multiple time points, the result with the longest time point was included in the analysis.
3) Safety Outcomes
  Any adverse events that occurred during the study should be recorded, such as the incidence of hypoglycemia, the incidence of adverse events, the incidence of serious adverse events and the incidence of gastrointestinal adverse reactions.

### Exclusion criteria

#### Type of studies

1) Studies designed as non-RCTs, such as cohort studies, case-control studies, cross-sectional studies, case reports, animal studies and reviews.
2) For any replicate studies, the one with more complete data was selected and the other study was excluded.
3) Meeting abstracts were excluded if no relevant data were provided.
4) Studies were excluded if the full text could not be obtained by searching online or contacting the authors.

#### Type of participants

1) Patients with acute metabolic disorders, such as diabetic ketoacidosis or infections.
2) Patients with severe hepatic and renal impairment, severe cardiovascular disease, pregnancy or lactation were excluded.

#### Type of interventions

The interventions used TCM treatments other than DCHD, such as acupuncture, moxibustion, massage, or acupoint injection.

#### Type of comparisons

The control group used measures other than conventional treatment.

#### Type of outcome measures

Studies with obvious data errors, incomplete data, questionable authenticity and lack of required indicators were excluded.

### Study selection and data extraction

The search results were imported into EndNote X9 software in the form of bibliography to establish a database. Two researchers independently screened the literature according to the inclusion and exclusion criteria. Firstly, duplicate literature was deleted. Secondly, the literature that did not meet the criteria was preliminarily screened by reading the title and abstract. The literature that was uncertain in the preliminary screening was browsed by reading the full text. After reading the full text, literature that still did not meet the criteria was excluded. If there was any difference, it was determined after discussion or consultation with XF and HX. Two researchers independently extracted data from included studies according to the pre-designed data extraction table. If some additional data are needed, we contacted the authors by email. The research data extracted mainly included: first author, publication year, study design, diagnostic criteria, sample size, gender, average age, course of disease, treatment duration, intervention measures, outcome indicators, comorbidity, adverse events and was cross-checked.

### Risk of bias assessment

The quality of the literature was assessed using the bias risk assessment tool in the Cochrane Handbook. This part was embedded in and implemented by Review Manager 5.4 software. This tool assessed seven important bias sources, including random sequence generation, allocation concealment, blinding of participants and personnel, blinding of outcome assessment, incomplete outcome data, selective reporting and other bias. Each included study was assessed for risk of bias from these seven aspects. By evaluating the completeness of research reporting and the correctness of methodological implementation, each aspect was assessed as “high risk,” “low risk” or “unclear risk”. Two researchers performed independently and examined each other. If there were different opinions on the evaluation results, the third researcher participated in the discussion and made the final decision.

### Statistical analysis

Review Manager 5.4 and Stata 16.0 were used for meta-analysis. For binary variables, the relative risk (RR) and 95% confidence interval (CI) were used to express the effect size. For continuous variables, when the same outcome indicator used the same unit, the mean difference (MD) and 95% CI were used to represent the effect size; otherwise, the standardized mean difference (SMD) and 95% CI were used. Heterogeneity was evaluated according to χ^2^ test and I^2^ test. If *p* > 0.1, I^2^ < 50%, it indicated that the heterogeneity between studies was small, and the fixed effect model was used to calculate the pooled effect size. If *p* ≤ 0.1, I^2^ ≥ 50%, it suggested significant statistical heterogeneity among the studies; therefore, the random effect model was used. Subgroup analysis and sensitivity analysis were performed to explore the source of heterogeneity and to judge the stability of the research results. The indicators of HbA1c, FBG and 2hPG included more than 10 studies, and we additionally performed a meta-regression on sample size, publication year and average age to explore the influence of these factors on heterogeneity. Meanwhile, we performed funnel chart and Egger’s test to evaluate publication bias on HbA1c, FBG and 2hPG. *p* > 0.05 indicated no obvious publication bias, and *p* < 0.05 indicated possible publication bias. Finally, we used the Grading of Recommendations Assessment, Development and Evaluation (GRADE) method to assess the evidence quality.

### Subgroup analysis

We prespecified factors that might influence treatment effect and performed subgroup analysis based on these prespecified assumptions to explore sources of heterogeneity. The following subgroup analyses were performed: Course of disease (≤5 years or >5 years); Treatment duration (≥3 months or <3 months); Average age (≤50 years old or >50 years old); Baseline BMI (≤24 or >24).

## Results

### Database search results

**FIGURE 1:**
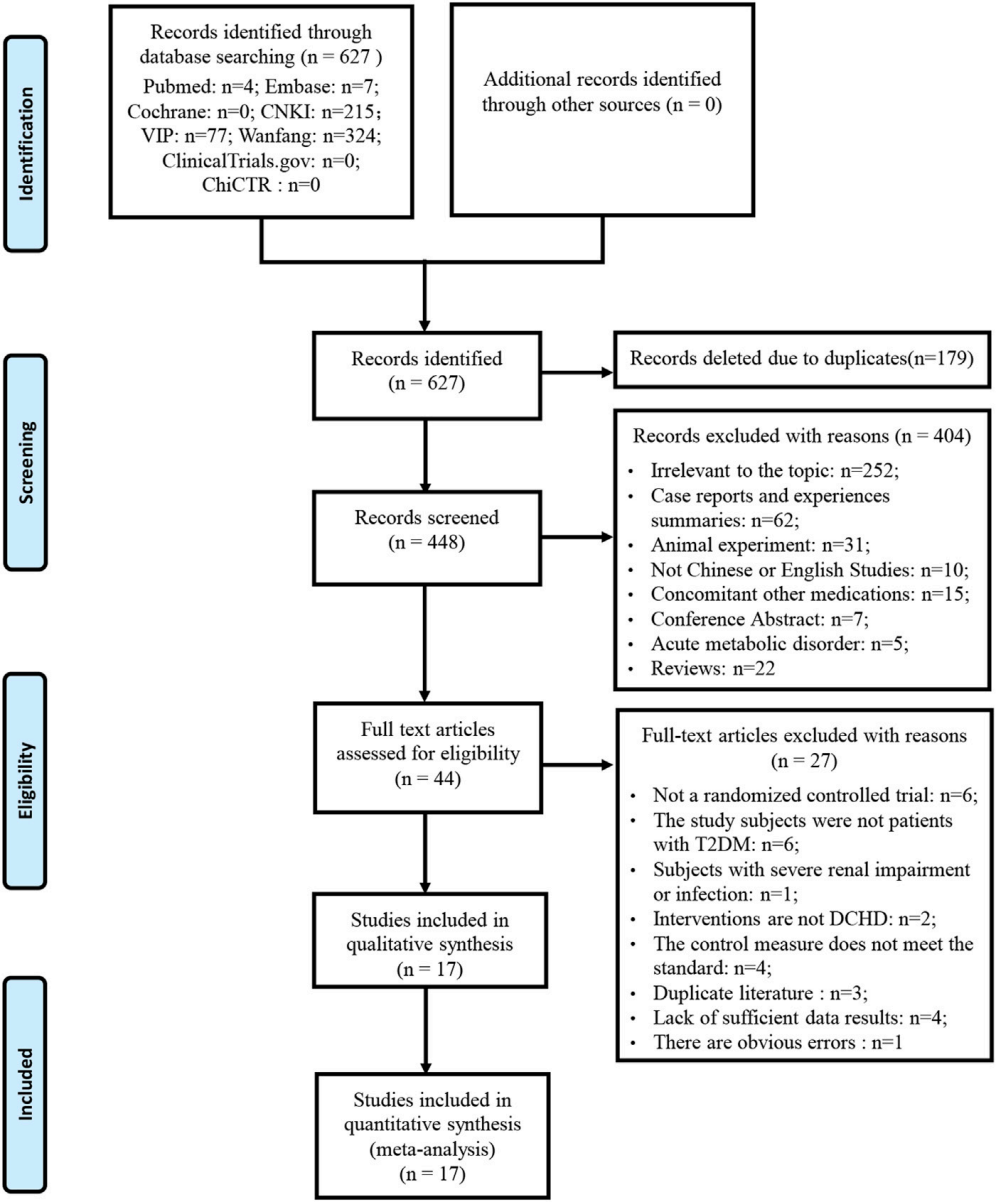
Flowchart of study selection and identification.

A total of 627 studies were retrieved by searching Chinese and English databases. 179 studies were excluded due to duplication. Of the remaining 448 studies, 404 studies were excluded by reading the titles and abstracts. The full texts of the remaining 44 studies were read, and 27 studies were excluded according to the inclusion and exclusion criteria. No additional studies were identified by screening references to relevant reviews and meta-analyses. Finally, 17 eligible studies were included in the quantitative analysis. The preliminary screening of the literature is provided in Supplementary Material S3. Literature excluded after reading the full text and reasons is listed in Supplementary Material S4. A detailed flowchart for screening eligible studies is shown in Figure 1.

### Characteristics of included studies

A total of 17 RCTs were included in this study, all completed in China and published between 2002 and 2021 (Liu, 2002; Shen and Wu, 2007; Cui and Chen, 2015; Zhao et al., 2016; Li et al., 2018; Zhang, 2018; Zhang et al., 2018; Gao, 2019; Li, 2019; Zhang H. S. et al., 2019; Bao et al., 2020; Chang, 2020; Ji and Che, 2020; Wang, 2020; Duan, 2021; Zhang Q. J. et al., 2021; Zou et al., 2021). A total of 1,525 patients with T2DM were included in this study, including 771 patients in experimental group and 754 patients in control group. One study (Ji and Che, 2020) evaluated the efficacy of metformin vs. DCHD alone and metformin vs. combination treatment, so we divided this study into part 1 and part 2. In terms of diagnostic criteria, the World Health Organization (WHO) definition was adopted in 10 studies (Shen and Wu, 2007; Cui and Chen, 2015; Zhao et al., 2016; Li et al., 2018; Zhang H. S. et al., 2019; Bao et al., 2020; part1- Ji and Che, 2020; part2- Ji and Che, 2020; Duan, 2021; Zhang Q. J. et al., 2021), the American Diabetes Association (ADA) definition was adopted in 2 studies (Liu, 2002; Zhang, 2018), the Chinese guideline diagnostic criteria were adopted in five studies (Zhang et al., 2018; Gao, 2019; Li, 2019; Wang, 2020; Zou et al., 2021), and the diagnostic criteria were not reported in one study (Chang, 2020). four studies (Zhang, 2018; Zhang H. S. et al., 2019; Bao et al., 2020; Zou et al., 2021) were treated with the original DCHD, and 14 studies (Liu, 2002; Shen and Wu, 2007; Cui and Chen, 2015; Zhao et al., 2016; Li et al., 2018; Zhang et al., 2018; Gao, 2019; Li, 2019; Chang, 2020; part1- Ji and Che, 2020; part2- Ji and Che, 2020; Wang, 2020; Duan, 2021; Zhang Q. J. et al., 2021) were treated with modified DCHD. The composition of DCHD or modified DCHD is shown in Table 1, and none of these studies reported the quality control or chemical analysis of DCHD. There were 13 studies (Shen and Wu, 2007; Zhang, 2018; Zhang et al., 2018; Gao, 2019; Li, 2019; Zhang H. S. et al., 2019; Bao et al., 2020; Chang, 2020; Ji and Che, 2020; Wang, 2020; Duan, 2021; Zhang Q. J. et al., 2021; Zou et al., 2021) in which the control group was treated with conventional treatment, and the treatment group was treated with DCHD or its modified on the basis of the control group. And there were four studies (Liu, 2002; Zhao et al., 2016; Li et al., 2018; Ji and Che, 2020) in which the control group was treated with conventional treatment, and the treatment group was treated with DCHD or its modified alone. Therefore, none of the above studies were sufficiently blinded. Only one study (Cui and Chen, 2015) claimed to have performed a placebo control. However, the blinding effect could not be achieved due to the difference in dosage form and frequency between placebo and DCHD. The basic characteristics of the included studies are shown in Table 2.

**TABLE 1:** Components of DCHD or its modified used in the included studies.

References	Formula	Components
Liu (2002)	modified DCHD	Chinese Thorowax Root (Chaihu, *Bupleurum falcatum* L.), Baical Skullcap Root (Huangqin, *Scutellaria baicalensis* Georgi), Golden thread (Huanglian, *Coptis chinensis* Franch.), Pinellia Tuber [Banxia, *Pinellia ternata* (Thunb.) Makino], Immature Orange Fruit (Zhishi, *Citrus aurantium* L.), Rhubarb (Dahuang, *Rheum palmatum* L.) and Smoked Plum (Wumei, *Prunus mume* (Siebold) Siebold and Zucc.).
Shen and Wu (2007)	modified DCHD	No specific components are mentioned
Cui and Chen (2015)	modified DCHD	Chinese Thorowax Root (Chaihu, *Bupleurum falcatum* L.) 15 g, Baical Skullcap Root (Huangqin, *Scutellaria baicalensis* Georgi) 15 g, Golden thread (Huanglian, *Coptis chinensis* Franch.) 10 g, Pinellia Tuber [Banxia, *Pinellia ternata* (Thunb.) Makino) 10 g, White Paeony Root (Baishao, *Paeonia lactiflora* Pall.) 25 g, Rhubarb (Dahuang, *Rheum palmatum* L.) 10 g, Immature Orange Fruit (Zhishi, *Citrus aurantium* L.) 15 g, Thomson Kudzuvine root [Gegen, *Pueraria montana* var. thomsonii (Benth.) M.R.Almeida] 20 g, Gypsum (Shigao, Gypsum Fibrosum) 30 g, Earthworm (Dilong, Pheretima) 15 g, Peach Seed (Taoren, *Prunus persica* (L.) Batsch) 15 g, Herba Hedyotidis (Baihuasheshecao, *Scleromitrion diffusum* (Willd.) R.J.Wang) 15 g, Common Anemarrhena Rhizome (Zhimu, *Anemarrhena asphodeloides* Bunge) 15 g and Danshen Root (Danshen, *Salvia miltiorrhiza* Bunge) 30 g. If dry mouth and thirst were identified, Figwort Root (Xuanshen, *Scrophularia ningpoensis* Hemsl.) 15 g and Snakegourd Root (Tianhuafen, *Trichosanthes kirilowii* Maxim.) 30 g were added. If shortness of breath and lack of strength were identified, Milkvetch Root (Huangqi, *Astragalus mongholicus* Bunge) 20 g and Tangshen (Dangshen, Codonopsis pilosula (Franch.) Nannf.) 15 g were added. If dizziness, headache and hypertension were identified, Chrysanthemum Flower (Juhua, *Chrysanthemum indicum* L.) 15 g, Oyster Shell (Shengmuli, Ostreae Concha) 30 g and Abalone Shell (Shijueming, Haliotidis Concha) 15 g were added. If slimy yellow tongue fur and damp-heat were identified, Largehead Atractylodes Rh (Baizhu, Atractylodes macrocephala Koidz.) 15 g and Chinese Gentian (Longdancao, *Gentiana* scabra Bunge) 10 g were added
Zhao et al. (2016)	modified DCHD	Chinese Thorowax Root (Chaihu, Bupleurum falcatum L.), Baical Skullcap Root (Huangqin, Scutellaria baicalensis Georgi), Rhubarb (Dahuang, Rheum palmatum L.), Immature Orange Fruit (Zhishi, Citrus aurantium L.), Pinellia Tuber (Banxia, Pinellia ternata (Thunb.) Makino), White Paeony Root (Baishao, Paeonia lactiflora Pall.), Chinese Gentian (Longdancao, *Gentiana* scabra Bunge), Hawthorn Fruit (Shanzha, Crataegus pinnatifida Bunge), Virgate Wormwood Herb (Yinchen, Artemisia capillaris Thunb.), Oriental Water Plantain Rhizome (Zexie, Alisma plantago-aquatica L.) and Danshen Root (Danshen, Salvia miltiorrhiza Bunge)
Zhang et al. (2018)	modified DCHD	Chinese Thorowax Root (Chaihu, *Bupleurum falcatum* L.) 20 g, Baical Skullcap Root (Huangqin, *Scutellaria baicalensis* Georgi) 20 g, Rhubarb (Dahuang, *Rheum palmatum* L.) 15 g, Golden thread (Huanglian, *Coptis chinensis* Franch.) 15 g, Immature Orange Fruit (Zhishi, *Citrus aurantium* L.) 15 g, Snakegourd Fruit (Gualou, *Trichosanthes kirilowii* Maxim.) 15 g, Pinellia Tuber [Banxia, *Pinellia ternata* (Thunb.) Makino] 10 g, White Paeony Root (Baishao, *Paeonia lactiflora* Pall.) 10 g and Liquorice Root (Gancao, *Glycyrrhiza glabra* L.) 6 g
Zhang (2018)	DCHD	Baical Skullcap Root (Huangqin, Scutellaria baicalensis Georgi) 9g, Pinellia Tuber (Banxia, Pinellia ternata (Thunb.) Makino) 8g, White Paeony Root (Baishao, Paeonia lactiflora Pall.) 9g, Immature Orange Fruit (Zhishi, Citrus aurantium L.) 9g, Fresh Ginger (Shengjiang, Zingiber officinale Roscoe) 16g, Rhubarb (Dahuang, Rheum palmatum L.) 7g, Chinese Thorowax Root (Chaihu, Bupleurum falcatum L.) 12 g and Chinese Date (Dazao, Ziziphus jujuba Mill.) 5 pieces
Li et al. (2018)	modified DCHD	Chinese Thorowax Root (Chaihu, *Bupleurum falcatum* L.) 10 g, Immature Orange Fruit (Zhishi, *Citrus aurantium* L.) 15 g, Baical Skullcap Root (Huangqin, *Scutellaria baicalensis* Georgi) 15 g, Rhubarb (Dahuang, *Rheum palmatum* L.) 8 g, Golden thread (Huanglian, *Coptis chinensis* Franch.) 15 g, White Paeony Root (Baishao, *Paeonia lactiflora* Pall.) 15 g, Common Anemarrhena Rhizome (Zhimu, Anemarrhena asphodeloides Bunge) 15 g and Dried Ginger (Ganjiang, *Zingiber officinale* Roscoe) 3 g. If dry mouth, thirst or increased eating with rapid hungering were identified, Thomson Kudzuvine root (Gegen, *Pueraria montana* var. thomsonii (Benth.) M. R. Almeida) and Snakegourd Root (Tianhuafen, *Trichosanthes kirilowii* Maxim.) were added. If incomplete and irregular bowel movements, slimy yellow tongue fur or slippery pulse were identified, Snakegourd Fruit Kernels (Gualouzi, *Trichosanthes kirilowii* Maxim.) and Pinellia Tuber (Banxia, Pinellia ternata (Thunb.) Makino) were added
Zhang H. S. et al. (2019)	DCHD	Chinese Thorowax Root (Chaihu, *Bupleurum falcatum* L.) 12 g, Rhubarb (Dahuang, *Rheum palmatum* L.) 8 g, Baical Skullcap Root (Huangqin, *Scutellaria baicalensis* Georgi) 12 g, Pinellia Tuber [Banxia, *Pinellia ternata* (Thunb.) Makino] 9 g, Immature Orange Fruit (Zhishi, *Citrus aurantium* L.) 12 g, Red Peony Root (Chishao, *Paeonia lactiflora* Pall.) 12 g, Chinese Date (Dazao, *Ziziphus jujuba* Mill.) 10 g and Fresh Ginger (Shengjiang, *Zingiber officinale* Roscoe) 10 g
Gao (2019)	modified DCHD	Chinese Thorowax Root (Chaihu, *Bupleurum falcatum* L.) 20 g, White Paeony Root (Baishao, *Paeonia lactiflora* Pall.) 10 g, Baical Skullcap Root (Huangqin, *Scutellaria baicalensis* Georgi) 20 g, Pinellia Tuber (Banxia, *Pinellia ternata* (Thunb.) Makino) 10 g, Immature Orange Fruit (Zhishi, *Citrus aurantium* L.) 15 g, Rhubarb (Dahuang, *Rheum palmatum* L.) 10 g, Liquorice Root (Gancao, *Glycyrrhiza glabra* L.) 6 g and Turmeric Root Tuber (Yujin, *Curcuma longa* L.) 15 g
Li (2019)	modified DCHD	Chinese Thorowax Root (Chaihu, *Bupleurum falcatum* L.) 20 g, Baical Skullcap Root (Huangqin, *Scutellaria baicalensis* Georgi) 20 g, Golden thread (Huanglian, *Coptis chinensis* Franch.) 15 g, Immature Orange Fruit (Zhishi, Citrus aurantium L.) 15 g, Rhubarb (Dahuang, *Rheum palmatum* L.) 15 g, Snakegourd Fruit (Gualou, *Trichosanthes kirilowii* Maxim.) 15 g, Pinellia Tuber (Banxia, *Pinellia ternata* (Thunb.) Makino) 10 g, White Paeony Root (Baishao, *Paeonia lactiflora* Pall.) 10 g and Liquorice Root (Gancao, *Glycyrrhiza glabra* L.) 6 g
part1-Ji and Che (2020)	modified DCHD	Chinese Thorowax Root (Chaihu, *Bupleurum falcatum* L.) 10 g, White Paeony Root (Baishao, *Paeonia lactiflora* Pall.) 15 g, Baical Skullcap Root (Huangqin, *Scutellaria baicalensis* Georgi) 10 g, Pinellia Tuber (Banxia, *Pinellia ternata* (Thunb.) Makino) 10 g, Immature Orange Fruit (Zhishi, *Citrus aurantium* L.) 10 g, Rhubarb (Dahuang, *Rheum palmatum* L.) 6 g, sickle senna (Jueming, *Senna tora* (L.) Roxb.) 15 g, Rhizoma Atractylodis (Cangzhu, *Atractylodes lancea* (Thunb.) DC.) 15 g, Figwort Root (Xuanshen, *Scrophularia ningpoensis* Hemsl.) 20 g and Turmeric Root Tuber (Yujin, *Curcuma longa* L.) 15 g
part2-Ji and Che (2020)	modified DCHD	Chinese Thorowax Root (Chaihu, *Bupleurum falcatum* L.) 10 g, White Paeony Root (Baishao, *Paeonia lactiflora* Pall.) 15 g, Baical Skullcap Root (Huangqin, *Scutellaria baicalensis* Georgi) 10 g, Pinellia Tuber (Banxia, *Pinellia ternata* (Thunb.) Makino) 10 g, Immature Orange Fruit (Zhishi, *Citrus aurantium* L.) 10 g, Rhubarb (Dahuang, *Rheum palmatum* L.) 6 g, sickle senna (Jueming, *Senna tora* (L.) Roxb.) 15 g, Rhizoma Atractylodis (Cangzhu, *Atractylodes lancea* (Thunb.) DC.) 15 g, Figwort Root (Xuanshen, *Scrophularia ningpoensis* Hemsl.) 20 g and Turmeric Root Tuber (Yujin, *Curcuma longa* L.) 15 g
Wang (2020)	modified DCHD	Chinese Thorowax Root (Chaihu, *Bupleurum falcatum* L.) 20 g, Golden thread (Huanglian, *Coptis chinensis* Franch.) 15 g, Baical Skullcap Root (Huangqin, *Scutellaria baicalensis* Georgi) 20 g, Immature Orange Fruit (Zhishi, *Citrus aurantium* L.) 15 g, Rhubarb (Dahuang, *Rheum palmatum* L.) 15 g, Snakegourd Fruit (Gualou, *Trichosanthes kirilowii* Maxim.) 15 g, White Paeony Root (Baishao, *Paeonia lactiflora* Pall.) 10 g, Pinellia Tuber [Banxia, *Pinellia ternata* (Thunb.) Makino] 10 g and Liquorice Root (Gancao, *Glycyrrhiza glabra* L.) 6 g
Bao et al. (2020)	DCHD	Chinese Thorowax Root (Chaihu, *Bupleurum falcatum* L.) 15 g, White Paeony Root (Baishao, *Paeonia lactiflora* Pall.) 10 g, Baical Skullcap Root (Huangqin, *Scutellaria baicalensis* Georgi) 10 g, Pinellia Tuber [Banxia, *Pinellia ternata* (Thunb.) Makino] 10g, Immature Orange Fruit (Zhishi, *Citrus aurantium* L.) 10 g, Fresh Ginger (Shengjiang, *Zingiber officinale* Roscoe) 10 g, Rhubarb (Dahuang, *Rheum palmatum* L.) 6 g and Chinese Date (Dazao, *Ziziphus jujuba* Mill.) 4 pieces
Chang (2020)	modified DCHD	Chinese Thorowax Root (Chaihu, *Bupleurum falcatum* L.) 15 g, White Paeony Root (Baishao, *Paeonia lactiflora* Pall.) 9 g, Baical Skullcap Root (Huangqin, *Scutellaria baicalensis* Georgi) 9 g, Pinellia Tuber (Banxia, *Pinellia ternata* (Thunb.) Makino) 9 g, Immature Orange Fruit (Zhishi, *Citrus aurantium* L.) 9 g, Rhubarb (Dahuang, *Rheum palmatum* L.) 9 g and Liquorice Root (Gancao, *Glycyrrhiza glabra* L.) 6 g
Zou et al. (2021)	DCHD	Chinese Thorowax Root (Chaihu, *Bupleurum falcatum* L.) 15 g, Rhubarb (Dahuang, *Rheum palmatum* L.) 6 g, Immature Orange Fruit (Zhishi, *Citrus aurantium* L.) 9 g, Baical Skullcap Root (Huangqin, *Scutellaria baicalensis* Georgi) 9 g, Pinellia Tuber (Banxia, *Pinellia ternata* (Thunb.) Makino) 9 g, White Paeony Root (Baishao, *Paeonia lactiflora* Pall.) 9 g, Chinese Date (Dazao, *Ziziphus jujuba* Mill.) 4 pieces and Fresh Ginger (Shengjiang, *Zingiber officinale* Roscoe) 15 g
Duan (2021)	modified DCHD	Chinese Thorowax Root (Chaihu, *Bupleurum falcatum* L.) 10 g, Rhubarb (Dahuang, *Rheum palmatum* L.) 10 g, Pinellia Tuber [Banxia, *Pinellia ternata* (Thunb.) Makino] 15 g, Baical Skullcap Root (Huangqin, *Scutellaria baicalensis* Georgi) 15 g, White Paeony Root (Baishao, *Paeonia lactiflora* Pall.) 15 g, Immature Orange Fruit (Zhishi, *Citrus aurantium* L.) 15 g, Chinese Date (Dazao, *Ziziphus jujuba* Mill.) 10 g, Fresh Ginger (Shengjiang, *Zingiber officinale* Roscoe) 6 g, Thomson Kudzuvine root (Gegen, *Pueraria montana* var. thomsonii (Benth.) M.R.Almeida) 20g, Danshen Root (Danshen, *Salvia miltiorrhiza* Bunge) 30g, Peach Seed (Taoren, *Prunus persica* (L.) Batsch) 15g, Common Anemarrhena Rhizome (Zhimu, *Anemarrhena asphodeloides* Bunge) 15 g and Earthworm (Dilong, Pheretima) 15 g. If dry mouth and thirst were identified, Figwort Root (Xuanshen, *Scrophularia ningpoensis* Hemsl.) 15 g and Snakegourd Root (Tianhuafen, *Trichosanthes kirilowii* Maxim.) 30 g were added. If shortness of breath and lack of strength were identified, Milkvetch Root (Huangqi, *Astragalus mongholicus* Bunge) 20 g and Tangshen (Dangshen, *Codonopsis pilosula* (Franch.) Nannf.) 15 g were added. If hypertension was identified, Chrysanthemum Flower (Juhua, *Chrysanthemum indicum* L.) 15 g, Oyster Shell (Shengmuli, *Ostreae Concha*) 30 g and Abalone Shell (Shijueming, Haliotidis Concha) 15 g were added. If greasy coating and damp-heat were identified, Largehead Atractylodes Rh (Baizhu, *Atractylodes macrocephala* Koidz.) 15 g and Chinese Gentian (Longdancao, *Gentiana scabra* Bunge) 10 g were added
Zhang Q. J. et al., 2021	modified DCHD	Chinese Thorowax Root (Chaihu, *Bupleurum falcatum* L.) 10 g, Baical Skullcap Root (Huangqin, *Scutellaria baicalensis* Georgi) 10 g, Immature Orange Fruit (Zhishi, *Citrus aurantium* L.) 10 g, Pinellia Tuber (Banxia, *Pinellia ternata* (Thunb.) Makino) 9 g, White Paeony Root (Baishao, *Paeonia lactiflora* Pall.) 20 g, Rhizoma Atractylodis (Cangzhu, *Atractylodes lancea* (Thunb.) DC.) 15 g, Chinese Cork-tree (Huangbo, *Phellodendron amurense* Rupr.) 15 g, Coix Seed (Yiyiren, Coix lacryma-jobi L.) 30 g, Glabrous Greenbrier Rhizome (Tufuling, *Smilax glabra* Roxb.) 30 g, Silkworm Excrement (Cansha, Faeces Bombycis) 30 g, Appendiculate Cremastra Pseudobulb (Shancigu, *Cremastra appendiculata* (D.Don) Makino) 10 g and Cyathulae Radix (Chuanniuxi, *Cyathula officinalis* K.C.Kuan) 15 g

DCHD, dachaihu decoction.

**TABLE 2:** The characteristics of the included studies.

First author (year)	Liu (2002)	Shen and Wu (2007)	Cui and Chen (2015)	Zhao et al. (2016)	Zhang et al. (2018)	Zhang et al. (2018)
Study design	RCT	RCT	RCT	RCT	RCT	RCT
Diagnostic criteria	1998 ADA	1999 WHO	1999 WHO	1999 WHO	2013 CDS	ADA
Sample size (randomized/analyzed) (E/C)	110/87; 52/35	49/44; 22/22	120/120; 60/60	120/120; 60/60	86/86; 43/43	120/120; 60/60
Gender (M/F) (E/C)	25/27; 16/19	13/12; 12/12	36/24; 20/40	32/28; 29/31	28/15; 27/16	30/30; 35/25
Average age (years) (E/C)	55.81 ± 10.54; 52.13 ± 11.29	41.5 (32–61)	42.5 ± 11.4; 40.3 ± 12.5	49.84 ± 4.28; 53.26 ± 4.15	50.5 ± 5.1; 50.1 ± 4.8	51.1 ± 6.2; 46.1 ± 7.2
Course of disease (years) (E/C)	7.69 ± 8.24; 6.52 ± 7.65	1.6 months (4 days-3 months)	6.2 ± 1.2; 5.8 ± 1.4	4.52 ± 0.46; 4.25 ± 0.48	7.2 ± 1.5; 7.0 ± 1.3	3.1 ± 1.4; 3.2 ± 2.1
Treatment duration	6 weeks	1 year	8 weeks	12 weeks	3 months	3 months
Co-intervention	Maintain the original treatment + Dietary intervention	Diabetes health education + Diet and exercise intervention	Diabetes health education + Diet and exercise intervention + Metformin, 0.5g, tid	Maintain the original treatment	NR	Diabetes health education + Diet and exercise intervention
Treatment group interventions	Modified DCHD, 6 g, tid	Modified DCHD, 100 ml/per, bid/tid + CG	Modified DCHD, 1 dose/per day, bid	Modified DCHD, 1 dose/per day	Modified DCHD, 1 dose/per day,bid + CG	DCHD, 400 ml, bid + CG
Control group interventions	Metformin, 0.5 g, bid	Intensive insulin therapy for 2 weeks: Novoline R subcutaneous injection before meals + Novoline N subcutaneous injection before bedtime; After 2 weeks: Novoline 30R or 50R subcutaneous injection 20–30 min before breakfast and dinner	Placebo, 6 pills, tid	Metformin, 0.5 g, tid	Liraglutide, 0.6 mg, qd, subcutaneous injection; If FBG>7.8 and 2hPG>11.8 mmol/L, liraglutide was increased to 1.2 mg qd. The dose is evaluated every 2 weeks, but the daily dose should not exceed 1.8 mg	Hypoglycemic agents
Outcome index	②③④⑤⑥⑦⑧	①②③	①②③	①②③⑤⑥⑦⑧⑨	①②③④⑨⑩	②③④
Baseline difference	NSD	NSD	NSD	NSD	NSD	NSD
Comorbidity	NR	NR	NR	Hyperlipemia	NR	NR
Adverse events	NR	NR	NR	No significant change in liver function	NR	NR
Country	CHINA	CHINA	CHINA	CHINA	CHINA	CHINA
Funding	NR	NR	NR	NR	NR	NR

First author (year)	Li et al. (2018)	Zhang H. S. et al. (2019)	Gao (2019)	Li (2019)	part1- Ji and Che (2020)	part2- Ji and Che (2020)
Study design	RCT	RCT	RCT	RCT	RCT	RCT
Diagnostic criteria	1999 WHO	2013 WHO	2013 CDS	CDS	1999 WHO	1999 WHO
Sample size (randomized/analyzed) (E/C)	102/102; 51/51	90/90; 45/45	76/76; 38/38	88/88; 44/44	40/40; 20/20	40/40; 20/20
Gender (M/F) (E/C)	31/20; 33/18	25/20; 30/15	21/17; 20/18	24/20; 23/21	11/9; 11/9	10/10; 11/9
Average age (years) (E/C)	52.8; 43.6	53.16 ± 9.37; 52.84 ± 10.26	50.97 ± 6.17; 51.64 ± 6.39	51.6 ± 2.5; 52.1 ± 2.3	55.33 ± 9.78; 56.50 ± 10.25	55.25 ± 9.93; 56.50 ± 10.25
Course of disease (years) (E/C)	0.3–5; 0.5–5	6.46 ± 3.73; 5.98 ± 4.09 (months)	NR	7.3 ± 2.3; 7.2 ± 2.1	5.54 ± 4.31; 5.35 ± 4.18	5.62 ± 4.37; 5.35 ± 4.18
Treatment duration	12 weeks	2 weeks	3 months	3 months	1 month	2 months
Co-intervention	Diet and exercise intervention + Blood glucose monitoring	Diabetes health education + Diet and exercise intervention + Blood glucose monitoring	NR	NR	NR	NR
Treatment group interventions	Modified DCHD, 1 dose/per day, bid	DCHD, 1 dose/per day, 100 ml/per, bid + CG	Modified DCHD, 1 dose/per day,bid + CG	Modified DCHD, 1 dose/per day, bid + CG	Modified DCHD, 1 dose/per day, 200 ml/per, bid	Modified DCHD, 1 dose/per day, 200 ml/per, bid + CG
Control group interventions	Metformin, 0.25g, tid	Gansulin R, 0.2 U/(kg·d), tid, subcutaneous injection 30 min before meals + Insulin glargine, 0.2U/(kg·d), qd, subcutaneous injection at 22PM + Metformin, 0.5g, bid	Liraglutide, initial dose 0.6 mg, qd, subcutaneous injection. The dose is increased to 1.2 mg within 1 week. The maximum daily dose is no more than 1.8 mg	Liraglutide, 0.6 mg, qd, subcutaneous injection. The maximum daily dose is no more than 1.8 mg	Metformin, 0.5 g, tid	Metformin, 0.5 g, tid
Outcome index	①②③④	②③	①②③⑨⑩	⑨⑩	①②③	①②③
Baseline difference	NSD	NSD	NSD	NSD	NSD	NSD
Comorbidity	NR	NR	NR	NR	NR	NR
Adverse events	NR	Incidence of hypoglycemia: 3 cases (6.64%) in experimental group; 11 cases (24.44%) in control group	NR	NR	NR	NR
Country	CHINA	CHINA	CHINA	CHINA	CHINA	CHINA
Funding	Hebei Provincial Administration of TCM Li Liping Inheritance Studio Project (No. Hebei TCM 2018-9]	NR	NR	NR	State Administration of TCM National Famous Chinese Medicine Experts Inheritance Studio Construction Project [Department of Human Resources and Education (2016) No. 42]

First author (year)	Wang (2020)	Bao et al. (2020)	Chang (2020)	Zou et al. (2021)	Duan (2021)	Zhang Q. J. et al. (2021)
Study design	RCT	RCT	RCT	RCT	RCT	RCT
Diagnostic criteria	2017 CDS	1999 WHO	NR	2017 CDS	WHO	1999 WHO
Sample size (randomized/analyzed) (E/C)	104/104; 52/52	60/60; 30/30	100/100; 50/50	60/60; 30/30	92/92; 46/46	100/96; 48/48
Gender (M/F) (E/C)	32/20; 31/21	19/11; 18/12	22/28; 24/26	18/12; 19/11	27/19; 29/17	41/9; 39/11
Average age (years) (E/C)	52.12 ± 6.47; 52.44 ± 6.58	44.23 ± 12.80; 40.18 ± 11.79	49.87 ± 7.21; 50.21 ± 8.90	55.51 ± 1.31; 55.47 ± 1.28	62.7 ± 1.4; 63.2 ± 1.8	47.2 ± 9.95; 46.86 ± 10.10
Course of disease (years) (E/C)	5.48 ± 1.77; 5.51 ± 1.82	10.00 ± 14.08; 8.90 ± 14.29 (months)	6.10 ± 1.38; 6.23 ± 1.41	NR	6.2 ± 0.4; 6.3 ± 0.7	7.36 ± 4.98; 7.46 ± 4.86
Treatment duration	12 weeks	12 weeks	2 months	14 days	NR	12 weeks
Co-intervention	Diabetes health education + Diet and exercise intervention	Diet and exercise intervention	Conventional treatment	NR	Diet and exercise intervention + Blood glucose monitoring	Diet intervention
Treatment group interventions	Modified DCHD, 1 dose/per day, 300 ml, bid + CG	DCHD, 1 dose/per day, 400 ml, bid + CG	Modified DCHD, 1 dose/per day, bid + CG	DCHD, 1 dose/per day, 150 ml/per, bid + CG	Modified DCHD, 1 dose/per day,tid + CG	Modified DCHD, 1 dose/per day, bid + CG
Control group interventions	Metformin, 0.25 g, tid	Exenatide, 5 μg, bid, subcutaneous injection; Exenatide, 10 μg, bid, subcutaneous injection (Four weeks later)	Insulin glargine, 0.2 IU/(kg·d), qn,subcutaneous injection. Adjust dose according to blood sugar level	Liraglutide, 0.6 mg, qd, subcutaneous injection + Insulin glargine, 0.2 IU/(kg·d), qd, subcutaneous injection	Conventional hypoglycemic drugs	Conventional hypoglycemic drugs
Outcome index	①②③④⑨⑩	①④⑤⑥⑨	①②③	①②③④⑤⑥⑧	①②③④	①②③④⑤⑥⑧⑨
Baseline difference	NSD	NSD	NSD	NSD	NSD	NSD
Comorbidity	NR	Non-alcoholic fatty liver disease	NR	NR	NR	Hyperuricemia
Adverse events	NR	NR	NR	NR	NR	Treatment group: 4 Loose stool; Control group: 5 abdominal distension; 2 nausea. No special treatment was given
Country	CHINA	CHINA	CHINA	CHINA	CHINA	CHINA
Funding	Key Science and Technology Project of Henan Province, No.092102310010	NR	NR	NR	NR	National Natural Science Foundation of China, No.82004345; Scientific Research Foundation of Guang ‘anmen Hospital, No. Y2018-07

Abbreviations: RCT, randomized controlled trial; ADA, american diabetes association; WHO, world health organization; CDS, chinese diabetes society; NR, not reported; DCHD, dachaihu decoction; CG, control group interventions; NSD, no significant difference; TCM, traditional Chinese medicine; Outcome index: ①: HbA1c; ②FBG; ③2hPG; ④BMI; ⑤TC; ⑥TG; ⑦HDL-C; ⑧LDL-C; ⑨HOMA-IR; ⑩HOMA-β.

### Risk of bias assessment

**FIGURE 2:**
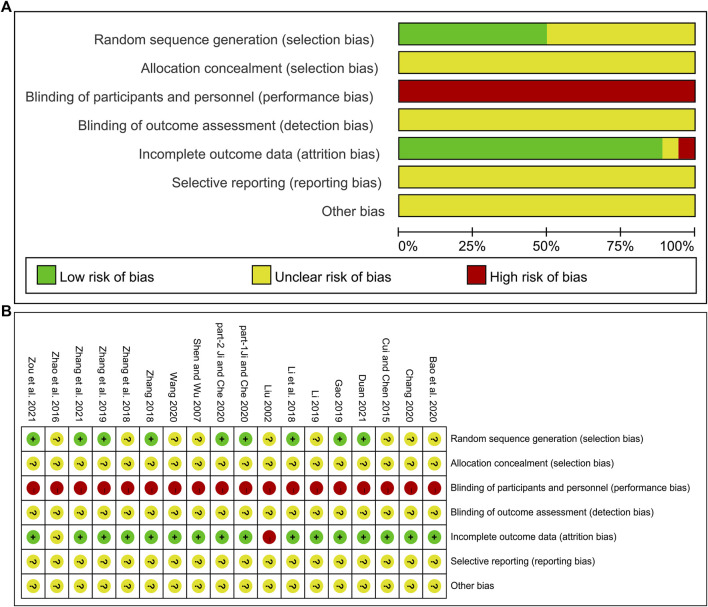
Risk of bias assessment for included studies: **(A)** Risk of bias graph. **(B)** Risk of bias summary.

The risk of bias was assessed using the Cochrane Risk of Bias tool. Of the included studies, eight (Li et al., 2018; Zhang, 2018; Gao, 2019; Zhang H. S. et al., 2019; part 1- Ji and Che, 2020; part 2- Ji and Che, 2020; Zhang Q. J. et al., 2021; Zou et al., 2021) used a random number table and one (Duan, 2021) used a computer-generated random sequence, and these studies were marked as low risk. Other studies claimed to have performed randomization but did not report the specific methods used in the random sequence generation, and these studies were marked as unclear risk. One study (Liu, 2002) used random assignment cards to conceal the random sequence. However, the concealment tightness of this method was not adequately described, and none of the other studies mentioned the assignment concealment method, so all studies were marked as unclear risk. In most studies, the control group was treated with conventional treatment and the experimental group was treated with DCHD alone or in combination with conventional treatment. One study (Cui and Chen, 2015) was placebo-controlled. The frequency and dosage form of the medication varied between experimental group and control group, so participants and researchers were not blinded, and these studies were rated as high risk. All studies did not state whether outcome assessors were blinded and were therefore marked as unclear risk. One study (Liu, 2002) was rated as high risk in outcome data completeness due to an imbalance in the number and reasons on missing patients between groups. One study (Zhao et al., 2016) did not report the number of patients in the two groups at the end of the study, so data integrity could not be judged, and the risk was assessed as unclear. The other studies had no incomplete data. Concerning selective reporting, we were unable to make judgments about the risk because none of the included studies were registered and no study protocols were available. None of the studies had sufficient information to determine whether there was another significant bias risk and were therefore assessed as unclear risk. In general, the methodological quality of the included literature was not high. The risk of bias assessment results for included studies is shown in Figure 2.

## Primary outcomes

### HbA1c

#### DCHD combined with conventional treatment vs. conventional treatment

**FIGURE 3:**
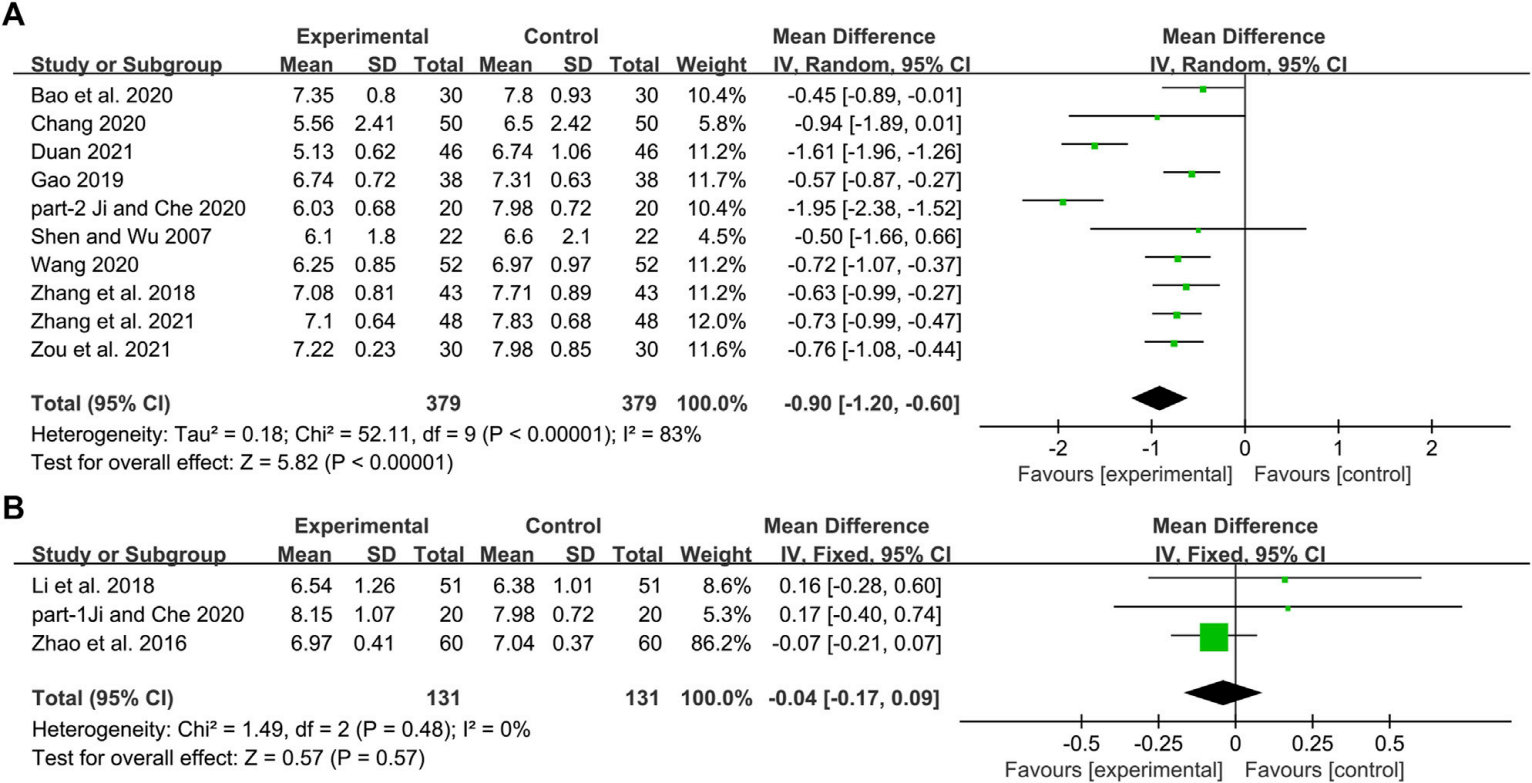
Forest plot of the HbA1c: **(A)** DCHD combined with conventional treatment vs. conventional treatment; **(B)** DCHD vs. conventional treatment.

**FIGURE 4:**
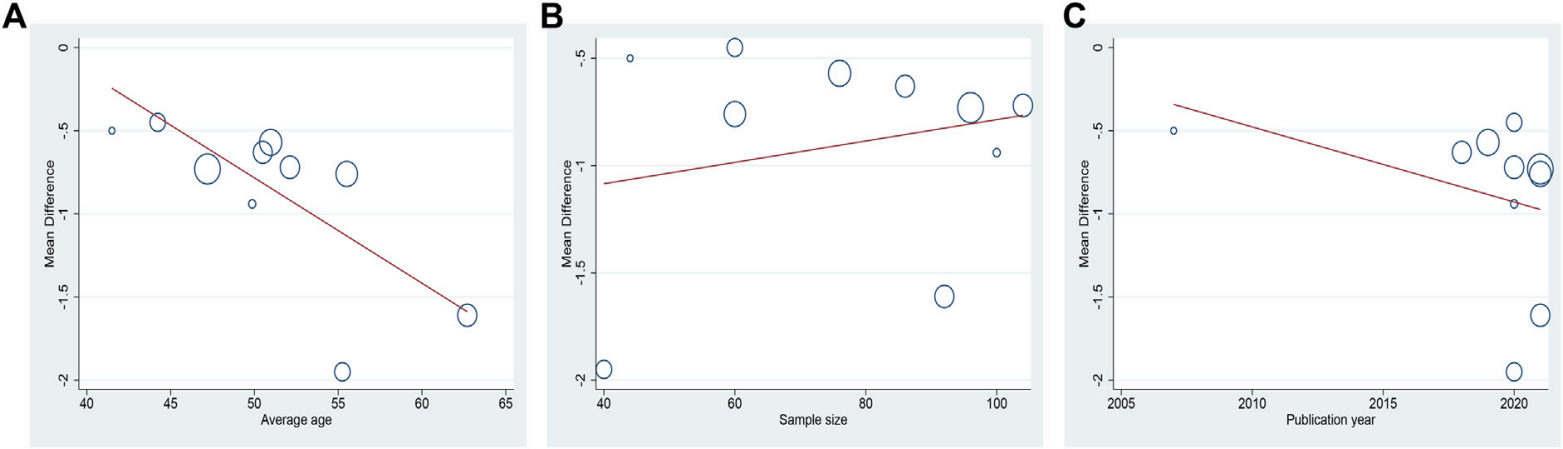
Meta-regression of the HbA1c for DCHD combined with conventional treatment vs. conventional treatment: **(A)** Average age; **(B)** Sample size; **(C)** Publication year.

**FIGURE 5:**
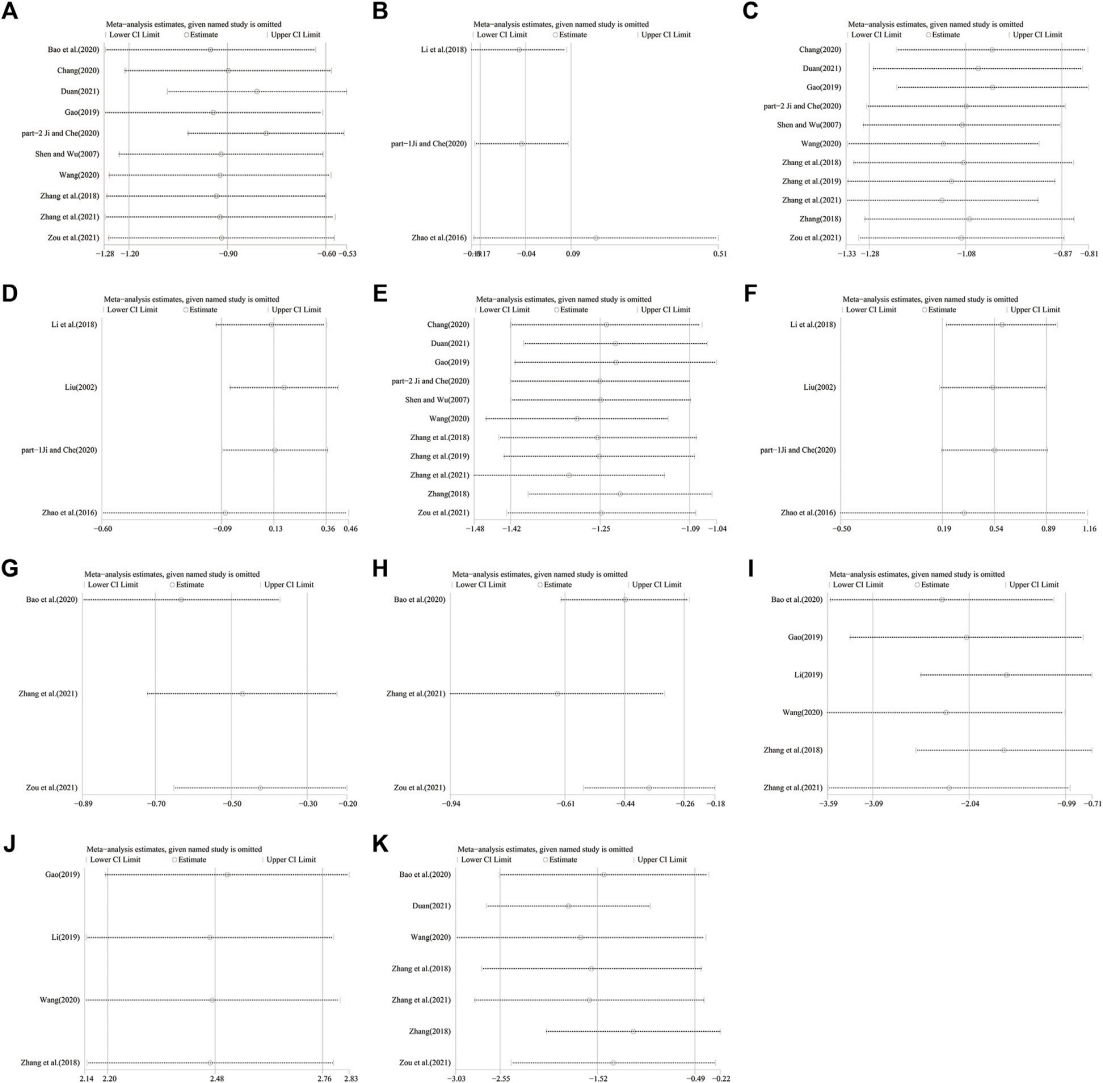
Sensitivity analysis: DCHD combined with conventional treatment vs. Conventional treatment: **(A)** HbA1c; **(C)** FBG; **(E)** 2hPG; **(G)** TC; **(H)** TG; **(I)** HOMA-IR; **(J)** HOMA-β; **(K)** BMI. DCHD vs. Conventional treatment: **(B)** HbA1c; **(D)** FBG; **(F)** 2hPG.

Ten studies including 758 patients reported the efficacy of DCHD combined with conventional treatment vs. conventional treatment alone on HbA1c (Shen and Wu, 2007; Zhang et al., 2018; Gao, 2019; Bao et al., 2020; Chang, 2020; Ji and Che, 2020; Wang, 2020; Duan, 2021; Zhang Q. J. et al., 2021; Zou et al., 2021). According to the heterogeneity test (*p* < 0.01, I^2^ = 83%), a random effect model was selected for statistical analysis. The pooled result showed that compared with conventional treatment, the combination with DCHD could reduce the HbA1c level, and the difference was statistically significant (MD = −0.90%, 95%CI: −1.20 to −0.60, *p* < 0.01) (Figure 3A). We performed meta-regression on average age, sample size and publication year to identify possible sources of heterogeneity. According to the meta-regression of age, the scatters distribution showed a linear regularity, and the Tau^2^ decreased from 0.18 to 0.10, which suggested that age may be the source of heterogeneity and could explain 53.55% of the variation between studies (*p* = 0.023, Adj R^2^ = 53.55%) (Figure 4A; Supplementary Material S5.1). We further analyzed from the regression diagram that the decrease in HbA1c gradually increased with age. In addition, sample size (*p* = 0.548, Adj R^2^ = -5.76%) and publication year (*p* = 0.442, Adj R^2^ = -0.33%) showed no significant difference on HbA1c (Figure 4B, C; Supplementary Material S5.2, S5.3). Study characteristics such as course of disease, treatment duration and baseline BMI may also contribute to heterogeneity. However, some studies incompletely reported these baseline characteristics, so we could not perform meta-regression on these factors and subgroup analysis was finally used. Subgroup analysis showed that the heterogeneity within each subgroup was not entirely reduced, so these factors cannot be considered as the source of heterogeneity at present (Supplementary Material S6). Sensitivity analysis was also performed, deleting one study at a time, and other studies were analyzed to estimate whether the results might have been significantly affected by a single study. Sensitivity analysis showed that the pooled effect sizes were similar and the result was robust (Figure 5A; Supplementary Material S7.1).

#### DCHD vs. conventional treatment

Three studies including 262 patients reported the efficacy of DCHD alone on HBA1c compared with conventional treatment (Zhao et al., 2016; Li et al., 2018; Ji and Che, 2020). According to the heterogeneity test (*p* = 0.48, I^2^ = 0%), a fixed effect model was selected for statistical analysis. The result showed that DCHD alone had the same reduction in HBA1c as conventional treatment, and the difference was not statistically significant (MD = -0.04%, 95%CI: −0.17 to 0.09, *p* = 0.57) (Figure 3B). Subgroup analysis showed that DCHD can reduce HBA1c in diabetic patients with different course of disease, treatment duration, age and baseline BMI (Supplementary Material S8). After excluding Zhao et al., 2016, the mean difference changed from MD = -0.04%, 95%CI: 0.17 to 0.09 to MD = 0.16%, 95%CI: −0.18 to 0.51. It showed that this study had a great influence on the result due to its relatively large sample size and small standard deviation. However, there was still no heterogeneity, and the result was still not statistically different, indicating that the result was relatively stable (Figure 5B, Supplementary Material S7.2 and S7.3).

#### DCHD vs. placebo

One study including 120 patients reported that DCHD could reduce the HbA1c level compared with placebo after 8 weeks treatment (MD = −0.35%, 95%CI: −0.68 to −0.02, *p* = 0.04) (Cui and Chen, 2015).

### FBG

#### DCHD combined with conventional treatment vs. conventional treatment

**FIGURE 6:**
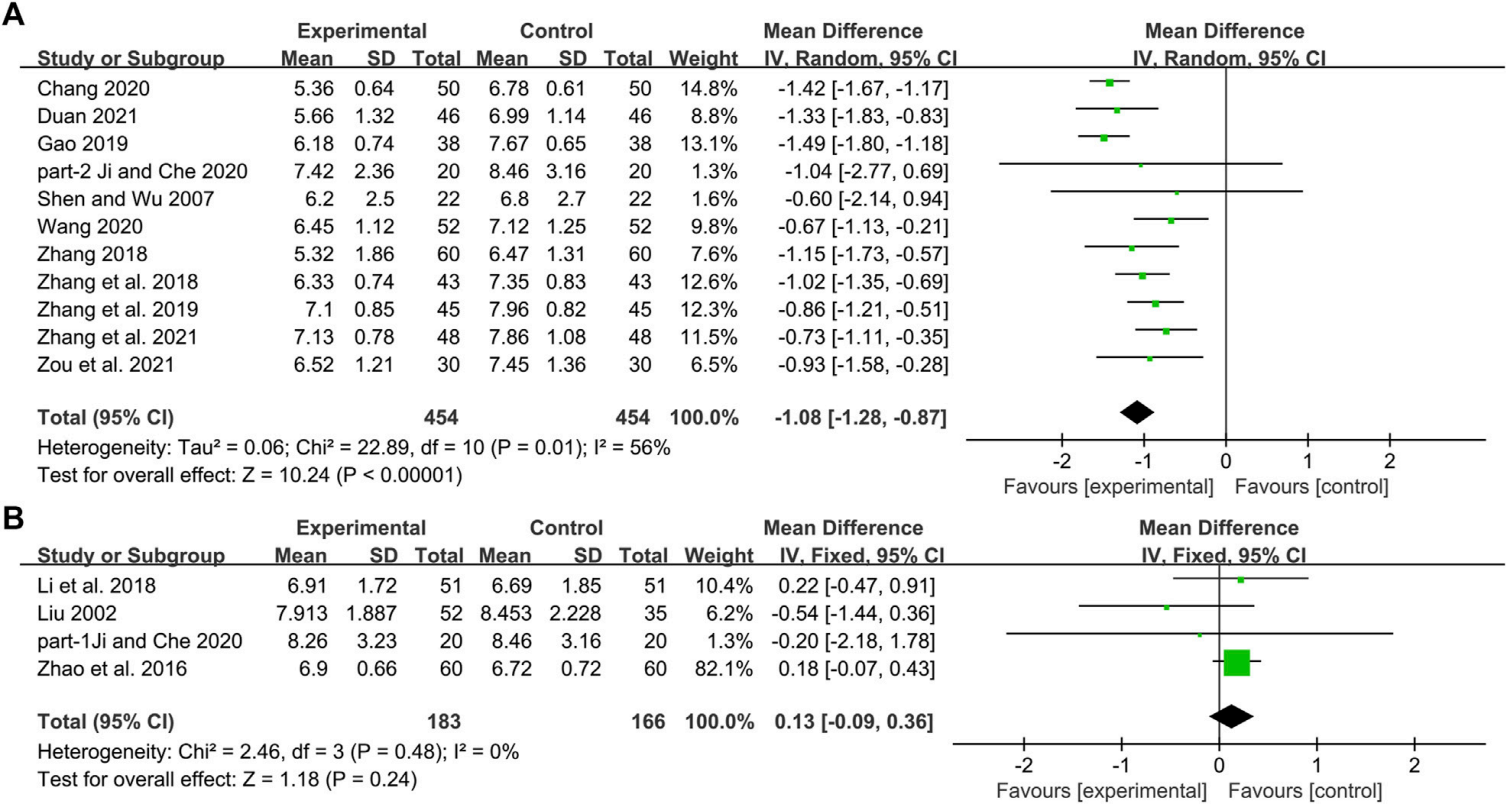
Forest plot of the FBG: **(A)** DCHD combined with conventional treatment vs. conventional treatment; **(B)** DCHD vs. conventional treatment.

**FIGURE 7:**
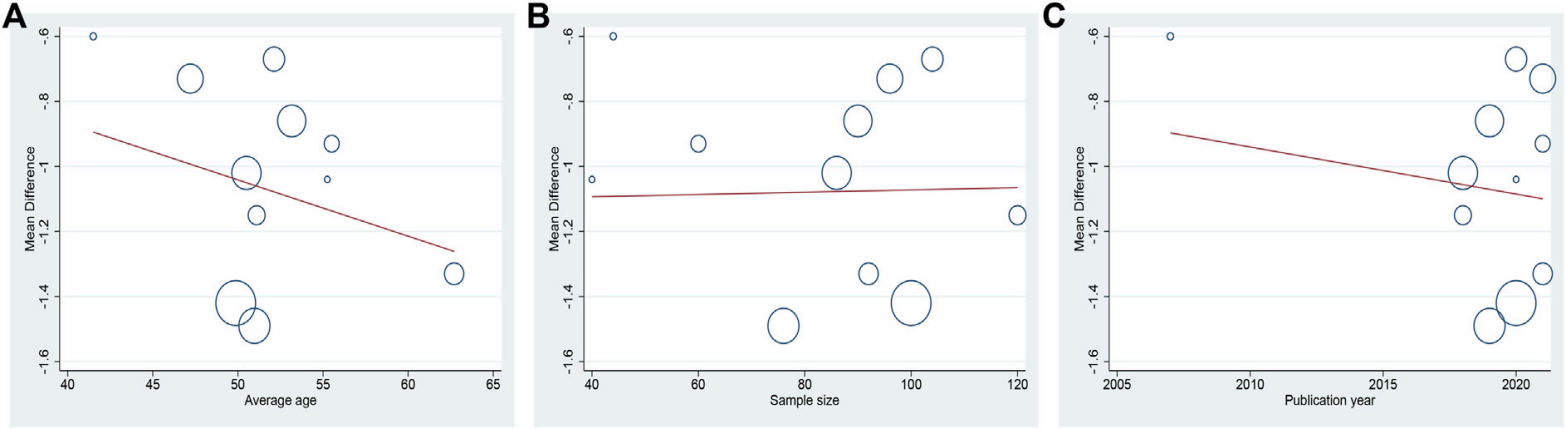
Meta-regression of the FBG for DCHD combined with conventional treatment vs. conventional treatment: **(A)** Average age; **(B)** Sample size; **(C)** Publication year.

Eleven studies including 908 patients reported the efficacy of DCHD combined with conventional treatment vs. conventional treatment alone on FBG (Shen and Wu, 2007; Zhang, 2018; Zhang et al., 2018; Gao, 2019; Zhang H. S. et al., 2019; Chang, 2020; Ji and Che, 2020; Wang, 2020; Duan, 2021; Zhang Q. J. et al., 2021; Zou et al., 2021). According to the heterogeneity test (*p* = 0.01, I^2^ = 56%), a random effect model was selected for statistical analysis. The result showed that compared with conventional treatment, combined with DCHD could reduce the FBG level, and the difference was statistically significant (MD = −1.08 mmol/L, 95%CI: −1.28 to −0.87, *p* < 0.01) (Figure 6A). Meta-regression was performed to identify possible sources of heterogeneity. Meta-regression according to average age (*p* = 0.532, Adj R^2^ = −12.30%), sample size (*p* = 0.959, Adj R^2^ = −10.99%) and publication year (*p* = 0.799, Adj R^2^ = −8.55%) showed no significant difference on FBG (Figure 7, Supplementary Material S5.4–S5.6). Due to limited information, we performed subgroup analyses on course of disease, treatment duration and baseline BMI to explore possible heterogeneity sources. Subgroup analyses according to course of disease and treatment duration showed that the heterogeneity within these two subgroups was not reduced, so they cannot be considered the source of heterogeneity (Supplementary Material S9A, S9B). The subgroup analysis according to different baseline BMI (≤24 or >24) showed significant subgroup difference (*p* < 0.01) and the heterogeneity within each group was reduced (I^2^ = 0% and 0%, respectively), which means the baseline BMI may be one of the reasons for the heterogeneity (Supplementary Material S9C). The result showed that compared with conventional treatment, the combination treatment had no statistical difference in reducing FBG in diabetic patients with BMI ≤ 24, but there was a significant statistical difference in patients with BMI > 24. Sensitivity analysis showed that the pooled statistics were similar and the result was robust (Figure 5C, Supplementary Material S7.4).

#### DCHD vs. conventional treatment

Four studies including 349 patients reported the efficacy of DCHD alone on FBG compared with conventional treatment (Liu, 2002; Zhao et al., 2016; Li et al., 2018; Ji and Che, 2020). According to the heterogeneity test (*p* = 0.48, I^2^ = 0%), the fixed effect model was applied. The pooled result did not find the difference between DCHD alone and conventional treatment in FBG (MD = 0.13 mmol/L, 95%CI: −0.09 to 0.36, *p* = 0.24) (Figure 6B), which means DCHD alone had the same reduction in FBG as conventional treatment. Subgroup analysis showed that DCHD can reduce FBG in diabetic patients with different age, course of disease, treatment duration and baseline BMI (Supplementary Material S10). After excluding Zhao et al., 2016, the mean difference changed from MD = 0.13 mmol/L, 95%CI: −0.09 to 0.36 to MD = −0.07 mmol/L, 95%CI: −0.60 to 0.46. It showed that this study had a great influence on the result. However, there was still no heterogeneity, and the result was still not statistically different, indicating that the result was relatively stable. (Figure 5D, Supplementary Material S7.5 and S7.6).

#### DCHD vs. placebo

One study including 120 patients reported that DCHD might result in a decrease in FBG compared with placebo after 8 weeks treatment (MD = −0.91 mmol/L, 95%CI: −1.29 to −0.53, *p* < 0.01) (Cui and Chen, 2015).

### 2hPG

#### DCHD combined with conventional treatment vs. conventional treatment

**FIGURE 8:**
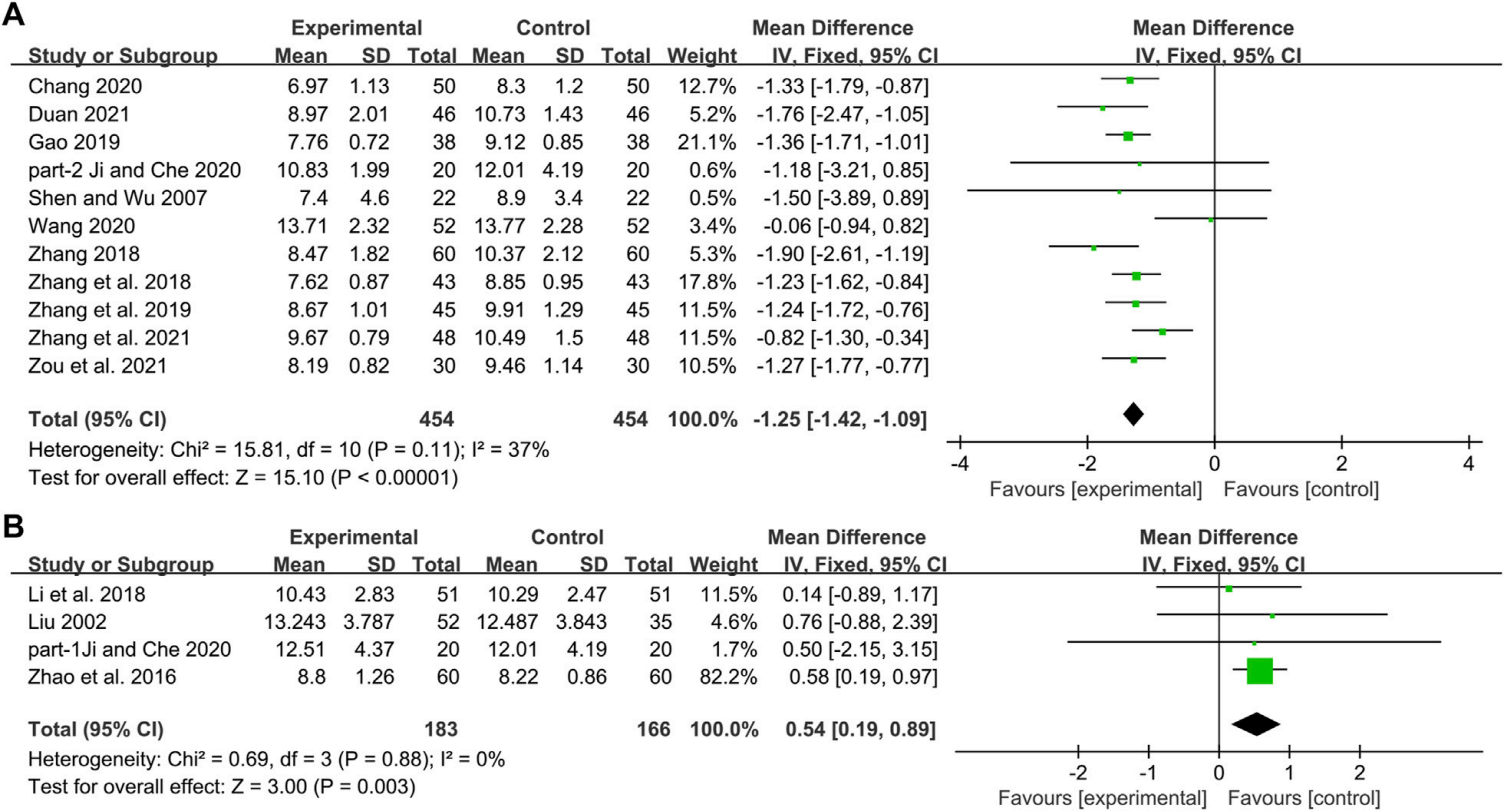
Forest plot of the 2hPG: **(A)** DCHD combined with conventional treatment vs. conventional treatment; **(B)** DCHD vs. conventional treatment.

Eleven studies including 908 patients reported the efficacy of DCHD combined with conventional treatment vs. conventional treatment alone on 2hPG (Shen and Wu, 2007; Zhang, 2018; Zhang et al., 2018; Gao, 2019; Zhang H. S. et al., 2019; Chang, 2020; Ji and Che, 2020; Wang, 2020; Duan, 2021; Zhang Q. J. et al., 2021; Zou et al., 2021). According to the heterogeneity test (*p* = 0.11, I^2^ = 37%), a fixed effect model was selected for statistical analysis. The result showed that compared with conventional treatment, combined with DCHD could significantly reduce the 2hPG level (MD = −1.25 mmol/L, 95%CI: −1.42 to −1.09, *p* < 0.01) (Figure 8A). Subgroup analysis was used to analyze the effect of the main study characteristics on 2hPG. Within each subgroup of age, course of disease and treatment duration, the effect sizes were statistically significant (*p* < 0.01), which means that DCHD may have a lowering effect on 2hPG in patients with different ages, course of disease and treatment duration (Supplementary material S11A-C). The subgroup analysis according to different baseline BMI (≤24 or >24) showed that compared with conventional treatment, the combination treatment had no statistical difference in reducing 2hPG in diabetic patients with BMI ≤ 24, but there was a significant statistical difference in patients with BMI > 24 (Supplementary Material S11D). Sensitivity analysis indicated that the results were stable (Figure 5E, Supplementary Material S7.7).

#### DCHD vs. conventional treatment

Four studies including 349 patients reported the efficacy of DCHD alone on 2hPG compared with conventional treatment (Liu, 2002; Zhao et al., 2016; Li et al., 2018; Ji and Che, 2020). According to the heterogeneity test (*p* = 0.88, I^2^ = 0%), the fixed effect model was applied. The pooled result found statistical significance between DCHD alone and conventional treatment in 2hPG (MD = 0.54 mmol/L, 95%CI: 0.19 to 0.89, *p* < 0.01) (Figure 8B). Subgroup analyses according to age, course of disease, treatment duration and baseline BMI were performed to explore the effect of these factors on the result (Supplementary Material S12). However, due to the small number of studies within each subgroup, the effect of different intervention levels and different patient baseline characteristics on the result cannot be clearly defined. Sensitivity analysis showed that Zhao et al., 2016 was highly sensitive. After excluding it, the pooled result was reversed and not statistically different (MD = 0.33 mmol/L, 95%CI: 0.50 to 1.16, *p* = 0.43) (Figure 5F, Supplementary Material S7.8, S7.9), indicating that the result is not robust. Zhao et al., 2016 has a larger weight in the pooled result due to its relatively large sample size, narrow confidence interval, and small standard deviation. Therefore, we are more convinced of the pooled effect size involved in this study, considering that the improvement on 2hPG by DCHD alone is not as good as that of conventional treatment. However, this still needs more high-quality research to verify.

#### DCHD vs. placebo

One study including 120 patients reported that there was no statistically significant difference in 2hPG between DCHD alone and placebo after 8 weeks treatment (MD = −0.53 mmol/L, 95%CI: −1.11 to 0.05, *p* = 0.07) (Cui and Chen, 2015).

## Secondary outcomes

### TC

#### DCHD combined with conventional treatment vs. conventional treatment

**FIGURE 9:**
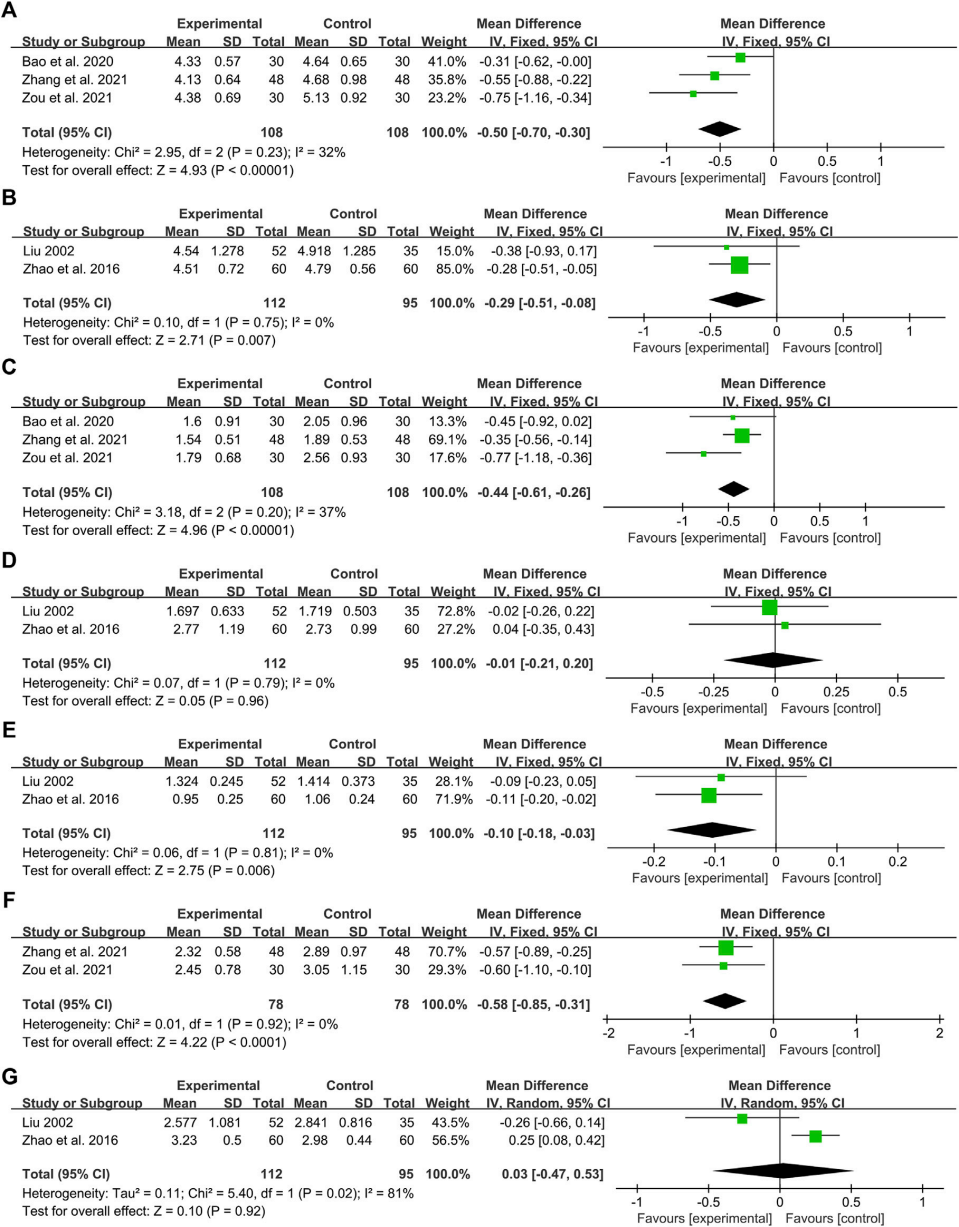
Forest plot of the lipid metabolism index: **(A)** TC: DCHD combined with conventional treatment vs. conventional treatment; **(B)** TC: DCHD vs. conventional treatment; **(C)** TG: DCHD combined with conventional treatment vs. conventional treatment; **(D)** TG: DCHD vs. conventional treatment; **(E)** HDL-C: DCHD vs. conventional treatment; **(F)** LDL-C: DCHD combined with conventional treatment vs. conventional treatment; **(G)** LDL-C: DCHD vs. conventional treatment.

Three studies including 216 patients reported the efficacy of DCHD combined with conventional treatment vs. conventional treatment alone on TC (Bao et al., 2020; Zhang Q. J. et al., 2021; Zou et al., 2021). According to the heterogeneity test (*p* = 0.23, I^2^ = 32%), a fixed effect model was used for statistical analysis. The pooled result illustrated that the combination with DCHD was remarkable for lowering TC compared with conventional treatment alone (MD = −0.50 mmol/L, 95%CI: −0.70 to −0.30, *p* < 0.01) (Figure 9A). Subgroup analyses according to age, course of disease and treatment duration were performed to explore the effect of these factors on the result (Supplementary Material S13). However, due to the small number of studies in each subgroup, the effect of different ages, course of disease and treatment duration on TC cannot be judged yet. Sensitivity analysis showed the result was robust (Figure 5G, Supplementary Material S7.10).

#### DCHD vs. conventional treatment

Two studies including 207 patients reported the efficacy of DCHD alone on TC compared with conventional treatment (Liu, 2002; Zhao et al., 2016). According to the heterogeneity test (*p* = 0.75, I^2^ = 0%), the fixed effect model was applied. The pooled effect suggested a greater impact of DCHD than conventional treatment (MD = −0.29 mmol/L, 95%CI: −0.51 to −0.08, *p* < 0.01) (Figure 9B). Switching to a random effect model did not change the significance of the result, suggesting that the result was robust (Supplementary Material S7.11).

#### DCHD vs. placebo

No study compared the effect of DCHD with placebo on TC.

### TG

#### DCHD combined with conventional treatment vs. conventional treatment

Three studies including 216 patients reported the efficacy of DCHD combined with conventional treatment vs. conventional treatment alone on TG (Bao et al., 2020; Zhang Q. J. et al., 2021; Zou et al., 2021). According to the heterogeneity test (*p* = 0.20, I^2^ = 37%), a fixed effect model was used for statistical analysis. The pooled result showed that the TG level of the combination treatment group was significantly lower than that of the conventional treatment group (MD = −0.44 mmol/L, 95%CI: −0.61 to −0.26, *p* < 0.01) (Figure 9C). Subgroup analyses according to age, course of disease and treatment duration were performed to explore the effect of these factors on the result (Supplementary Material S14). However, due to the small number of studies in each subgroup, the effect of different ages, course of disease and treatment duration on TG cannot be judged yet. Sensitivity analysis showed the results were robust (Figure 5H, Supplementary Material S7.12).

#### DCHD vs. conventional treatment

Two studies including 207 patients reported the efficacy of DCHD alone on TG compared with conventional treatment (Liu, 2002; Zhao et al., 2016). According to the heterogeneity test (*p* = 0.79, I^2^ = 0%), the fixed effect model was applied. The pooled effect indicated that there was no significant difference between the DCHD group and the conventional treatment group (MD = −0.01 mmol/L, 95%CI: −0.21 to 0.20, *p* = 0.96) (Figure 9D). Switching to a random effect model did not change the result, suggesting that the result was robust (Supplementary Material S7.13).

#### DCHD vs. placebo

No study compared the effect of DCHD with placebo on TG.

### HDL-C

#### DCHD combined with conventional treatment vs. conventional treatment

No study compared the effect of combination treatment with conventional treatment on HDL-C.

#### DCHD vs. conventional treatment

Two studies including 207 patients reported the efficacy of DCHD alone on HDL-C compared with conventional treatment (Liu, 2002; Zhao et al., 2016). According to the heterogeneity test (*p* = 0.81, I^2^ = 0%), the fixed effect model was used. The pooled effect indicated that the HDL-C level in the DCHD group was much lower (MD = −0.10 mmol/L, 95%CI: −0.18 to −0.03, *p* < 0.01) (Figure 9E). Switching to a random effect model did not change the significance of the result, suggesting that the result was robust (Supplementary Material S7.14).

#### DCHD vs. placebo

No study compared the effect of DCHD with placebo on HDL-C.

### LDL-C

#### DCHD combined with conventional treatment vs. conventional treatment

Two studies including 156 patients reported the efficacy of DCHD combined with conventional treatment vs. conventional treatment alone on LDL-C (Zhang Q. J. et al., 2021; Zou et al., 2021). According to the heterogeneity test (*p* = 0.92, I^2^ = 0%), the fixed effect model was used. The pooled effect indicated that the LDL-C level in the DCHD group was much lower (MD = −0.58 mmol/L, 95%CI: −0.85 to −0.31, *p* < 0.01) (Figure 9F). Switching to a random effect model did not change the significance of the result, suggesting that the result was robust (Supplementary Material S7.15).

#### DCHD vs. conventional treatment

Two studies including 207 patients reported the efficacy of DCHD alone on LDL-C compared with conventional treatment (Liu, 2002; Zhao et al., 2016). According to the heterogeneity test (*p* = 0.02, I^2^ = 81%), the random effect model was used. The pooled effect indicated that there was no significant difference between the DCHD group and the conventional treatment group on LDL-C (MD = 0.03 mmol/L, 95%CI: −0.47 to 0.53, *p* = 0.92) (Figure 9G). Switching to a fixed effect model made the result statistically different, suggesting that the result is not robust (Supplementary Material S7.16). Due to the small number of included studies and the large differences in results between studies, we are not yet able to determine the efficacy of DCHD alone on LDL-C.

#### DCHD vs. placebo

No study compared the effect of DCHD with placebo on LDL-C.

### HOMA-IR

#### DCHD combined with conventional treatment vs. conventional treatment

**FIGURE 10:**
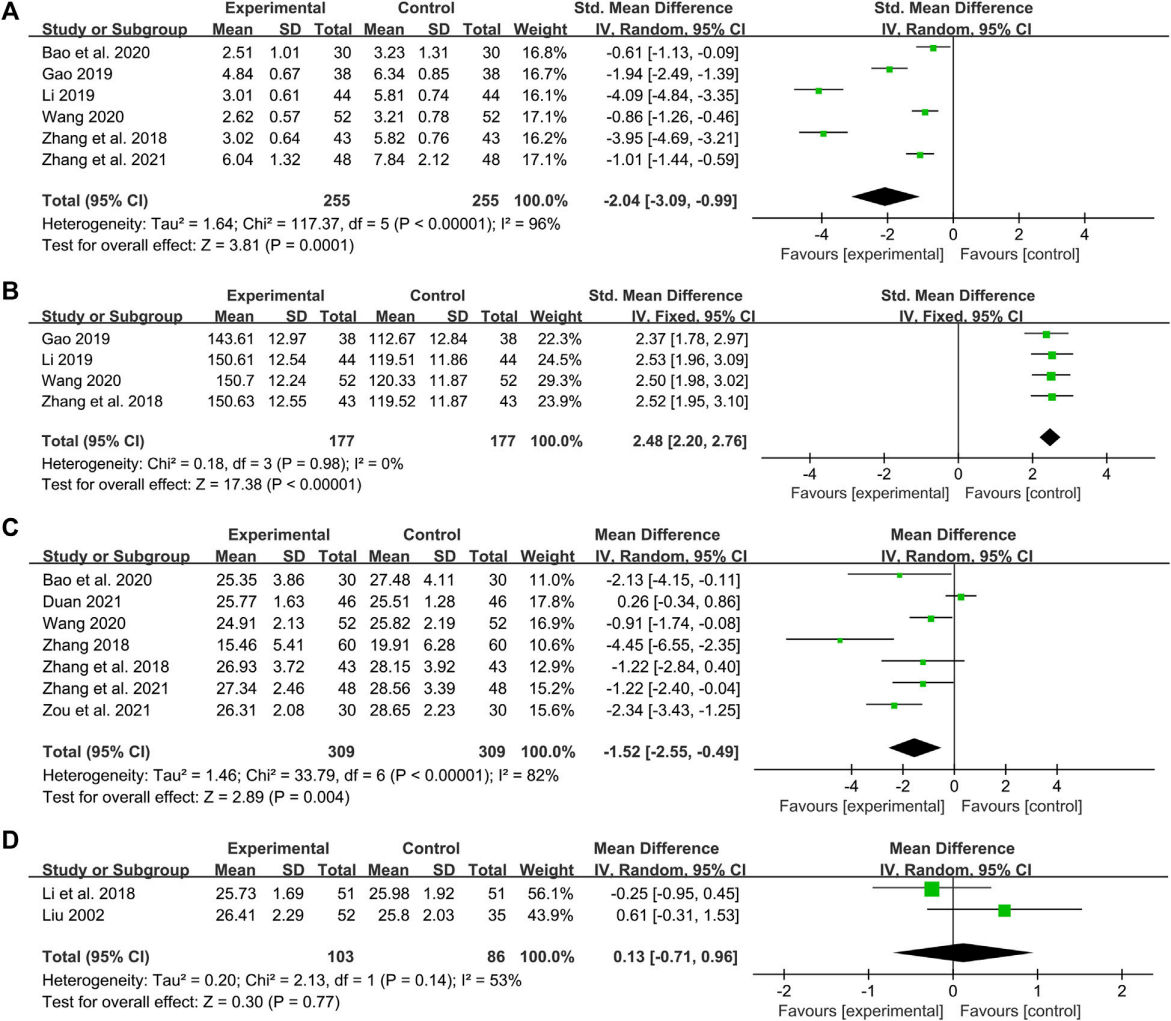
Forest plot of the HOMA-IR, HOMA-β and BMI: **(A)** HOMA-IR: DCHD combined with conventional treatment vs. conventional treatment; **(B)** HOMA-β: DCHD combined with conventional treatment vs. conventional treatment; **(C)** BMI: DCHD combined with conventional treatment vs. conventional treatment; **(D)** BMI: DCHD vs. conventional treatment.

Six studies including 510 patients reported the efficacy of DCHD combined with conventional treatment vs. conventional treatment alone on HOMA-IR (Zhang et al., 2018; Gao, 2019; Li, 2019; Bao et al., 2020; Wang, 2020; Zhang Q. J. et al., 2021). According to the heterogeneity test (*p* < 0.01, I^2^ = 96%), a random effect model was used. The pooled result showed that HOMA-IR in the combination treatment group was significantly lower than that in the conventional treatment group (SMD = −2.04, 95%CI: −3.09 to −0.99, *p* < 0.01) (Figure 10A). Subgroup analyses according to age and course of disease showed that the heterogeneity within these subgroups was not reduced, so they cannot be considered the sources of heterogeneity at present (Supplementary Material S15). We speculated that the heterogeneity might be related to the large individual differences in HOMA-IR, leading to the large differences in baseline HOMA-IR among study points. It may also be related to the measurement bias caused by the different insulin detection methods. Sensitivity analysis showed that the pooled statistics were similar and the result was robust (Figure 5I, Supplementary Material S7.17).

#### DCHD vs. conventional treatment

One study including 120 patients reported that there was no significant difference in improving HOMA-IR between DCHD alone and conventional treatment after 12 weeks treatment (MD = −0.20, 95%CI: −0.75 to 0.35, *p* = 0.47) (Zhao et al., 2016).

#### DCHD vs. placebo

No study compared the effect of DCHD with placebo on HOMA-IR.

### HOMA-β

#### DCHD combined with conventional treatment vs. conventional treatment

Four studies including 354 patients reported the efficacy of DCHD combined with conventional treatment vs. conventional treatment alone on HOMA-β (Zhang et al., 2018; Gao, 2019; Li, 2019; Wang, 2020). According to the heterogeneity test (*p* = 0.98, I^2^ = 0%), a fixed effect model was used. The result revealed a significant increase in HOMA-β with combination treatment than with conventional treatment alone (SMD = 2.48, 95%CI: 2.20 to 2.76, *p* < 0.01) (Figure 10B). Subgroup analyses were not performed because the included studies could not be grouped by prespecified factors. Sensitivity analysis showed that the pooled statistics were similar and the result was robust (Figure 5J, Supplementary Material S7.18).

#### DCHD vs. conventional treatment

No study compared the effect of DCHD with conventional treatment on HOMA-β.

#### DCHD vs. placebo

No study compared the effect of DCHD with placebo on HOMA-β.

### BMI

#### DCHD combined with conventional treatment vs. conventional treatment

Seven studies including 618 patients reported the efficacy of DCHD combined with conventional treatment vs. conventional treatment alone on BMI (Zhang, 2018; Zhang et al., 2018; Bao et al., 2020; Wang, 2020; Duan, 2021; Zhang Q. J. et al., 2021; Zou et al., 2021). According to the heterogeneity test (*p* < 0.01, I^2^ = 82%), a random effect model was used for statistical analysis. The pooled result illustrated that the combination treatment was remarkable for lowering BMI in contrast with conventional treatment alone (MD = −1.52 kg/m^2^, 95%CI: −2.55 to −0.49, *p* < 0.01) (Figure 10C). Subgroup analysis according to different course of disease showed reduced heterogeneity between subgroups (I^2^ = 59% and 66%, respectively), which means the course of disease may be a source of heterogeneity (Supplementary Material S16A). It also indicated that DCHD might reduce BMI in patients with course of disease ≤5 years, but not in patients with course of disease >5 years. Subgroup analysis according to age showed no reduction in heterogeneity, so age cannot yet be considered a source of heterogeneity (Supplementary Material S16B). Subgroup analysis according to treatment duration was also performed. However, due to limited information, we could not judge whether the treatment duration was the source of heterogeneity (Supplementary Material S16C). Sensitivity analysis indicated that the result was stable (Figure 5K, Supplementary Material S7.19).

#### DCHD vs. conventional treatment

Two studies including 189 patients reported the efficacy of DCHD alone on BMI compared with conventional treatment (Liu, 2002; Li et al., 2018). According to the heterogeneity test (*p* = 0.14, I^2^ = 53%), the random effect model was applied. The pooled effect showed no significant difference between DCHD alone and conventional treatment (MD = 0.13 kg/m^2^, 95%CI: −0.71 to 0.96, *p* = 0.77) (Figure 10D), which means that DCHD alone may be as effective as conventional treatment in reducing BMI. Switching to a fixed effect model did not change the result, suggesting that the result was robust (Supplementary Material S7.20).

#### DCHD vs. placebo

No study compared the effect of DCHD with placebo on BMI.

### Adverse events

**FIGURE 11:**
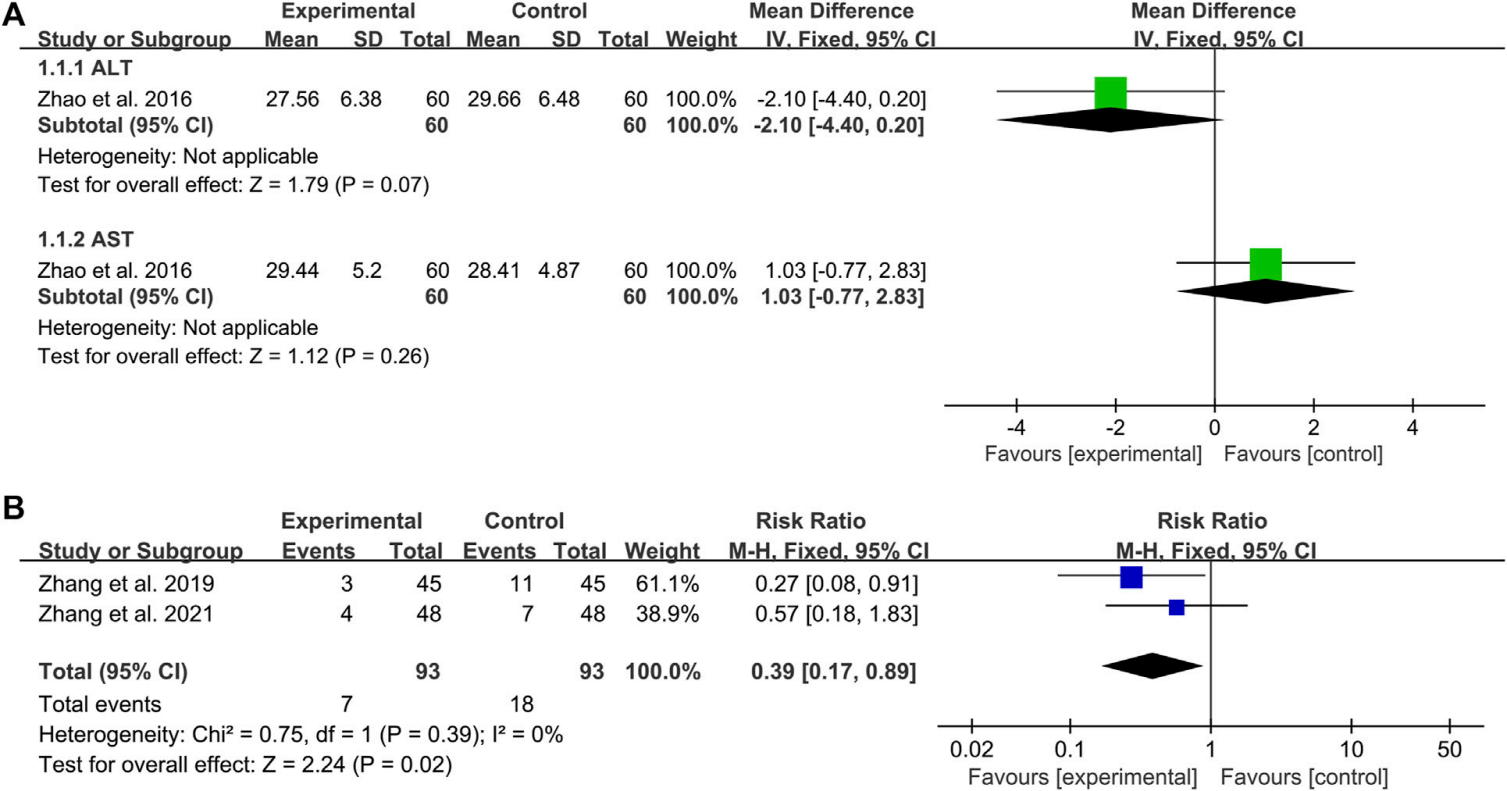
Forest plot of the adverse events: **(A)** liver function; **(B)** incidence of adverse events.

Of the 17 included studies, only three studies reported adverse events. Zhao et al., 2016 reported that DCHD alone had no significant effect on liver function compared with conventional treatment (ALT: MD = −2.10 U/L, 95%CI: −4.40 to 0.20, *p* = 0.07; AST: MD = 1.03 U/L, 95%CI: −0.77 to 2.83, *p* = 0.26) (Figure 11A). Zhang H. S. et al. (2019) reported that there were three cases of hypoglycemia (6.67%) in the combination treatment group, and 11 cases of hypoglycemia (24.44%) in the conventional treatment group. Compared with the conventional treatment, the combination treatment could reduce the incidence of hypoglycemia, and the difference was statistically significant. Zhang Q. J. et al. (2021) reported four cases of loose stool (8.33%) in the combination treatment group, five cases of abdominal distention and two cases of nausea (14.58%) in the conventional treatment group, all of which were gradually relieved without special treatment. The pooled result showed that the incidence of adverse events in the combination treatment group was significantly lower than that in the conventional treatment group (RR = 0.39, 95%CI: 0.17 to 0.89, *p* = 0.02) (Figure 11B). None of the other 14 studies mentioned adverse events. The safety indicators assessed in these three studies included liver function, incidence of hypoglycemia and gastrointestinal adverse reactions, and no serious adverse events were observed. The results indicated that DCHD is relatively safe. However, due to the small sample size, more studies are still needed to further confirm its safety.

### Publication bias

**FIGURE 12:**
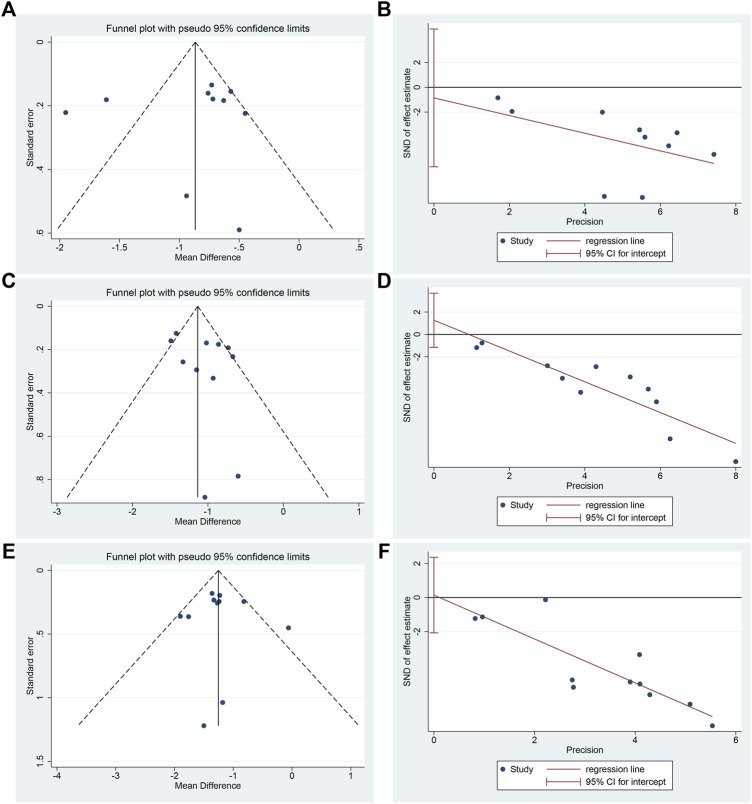
Publication bias of HBA1c, FBG and 2hPG: **(A)** Funnel plot of HBA1c; **(B)** Egger’s test of HBA1c; **(C)** Funnel plot of FBG; **(D)** Egger’s test of FBG; **(E)** Funnel plot of 2hPG; **(F)** Egger’s test of 2hPG.

Funnel plot and Egger’s test were used to assess publication bias on HBA1c, FBG and 2hPG. The funnel plot of HBA1c showed an asymmetric left-right distribution among study points, and two studies deviated far from the combined effect size, suggesting that there may be a large heterogeneity among studies (Figure 12A). Egger’s test showed no statistical difference (*p* = 0.732) (Figure 12B, Supplementary Material S17.1), indicating that there was no obvious publication bias in the studies of HBA1c. The funnel plots of FBG and 2hPG showed roughly symmetrical distribution, which was consistent with Egger’s test results (*p* = 0.266 and 0.886, respectively) (Figure 12C–F, Supplementary Material S17.2, S17.3), suggesting that there was no obvious publication bias in the studies of FBG and 2hPG.

### Assessment of evidence quality

The evidence quality was assessed by the GRADE method. The overall evidence quality for each outcome was moderate to very low by assessment. The evidence quality declined mainly due to the high risk of bias, inconsistency between studies and imprecision in results. A summary of the overall evidence for each outcome is presented in Supplementary Material S18.

## Discussion

### Main results of this research

T2DM is an endocrine disease caused by defective insulin secretion and decreased function in regulating glucose metabolism, manifesting as chronic hyperglycemia and nutrient metabolism disorder (Scheen, 2003). During its development, glucose and lipid metabolism abnormality and insulin resistance form a vicious circle, which leads to the progressive decline of islet β-cell function, and finally promotes the occurrence and development of diabetes and its complications (LeRoith, 2002). DCHD is a classical formula derived from TCM, and several clinical studies have found that its application in the T2DM treatment may achieve better results.

In this study, we analyzed the efficacy and safety of DCHD in the T2DM treatment for the first time, providing the latest systematic evidence for the application of DCHD. We conducted a comprehensive search on both Chinese and English databases and performed a detailed analysis on the outcome indicators from the perspective of glucose and lipid metabolism, insulin resistance, islet function and BMI. Sources of heterogeneity were also explored by using meta-regression and subgroup analysis, and the evidence quality was assessed by GRADE.

Both original DCHD and modified DCHD were included. In TCM, each classical formula is a fixed whole with a definite herbal composition and efficacy. DCHD is no exception. It can be considered for T2DM patients with heat stagnation in liver and stomach syndrome. However, patients’ symptoms are varied clinically, so it is necessary to add or subtract herbs based on the original DCHD according to the accompanying symptoms and individual differences. In this way, the formula can be better adapted to the patient’s situation and thus exert a better effect. This is syndrome differentiation and treatment, the most important feature of TCM in disease treatment. These modified formulas have similar composition and treatment concepts as the original one and are better adapted to different patient conditions, so they can be considered the same category.

Through comprehensive analysis, some findings were obtained. A total of 627 articles were retrieved and 17 were finally included for meta-analysis. Risk of bias assessment revealed that the methodological quality of the included studies was not high, which was mainly related to the lack of detailed reporting on the specific methods of random sequence generation and allocation concealment, as well as the inadequate implementation of blinding.

Our main finding was that DCHD, in combination or alone, resulted in a statistically significant reduction in blood sugar. Compared with conventional treatment alone, the combination with DCHD can significantly improve FBG (MD = −1.08 mmol/L, 95%CI: −1.28 to −0.87, *p* < 0.01), 2hPG (MD = −1.25 mmol/L, 95%CI: −1.42 to −1.09, *p* < 0.01) and HbA1c (MD = −0.90%, 95%CI: −1.20 to −0.60, *p* < 0.01); When used alone, compared with conventional treatment, DCHD had the similar effect in improving FBG (MD = 0.13 mmol/L, 95%CI: −0.09 to 0.36, *p* = 0.24) and HbA1c (MD = −0.04%, 95%CI: −0.17 to 0.09, *p* = 0.57), and can also reduce 2hPG, even if not as effective as conventional treatment (MD = 0.54 mmol/L, 95%CI: 0.19 to 0.89, *p* < 0.01). It can be seen that combined treatment can achieve a better double hypoglycemic effect, which fully reflects the advantages of integrated traditional Chinese and Western medicine. The improvement of HbA1c by the combined DCHD treatment benefited from the decrease of both FBG and 2hPG. HbA1c reflects the average blood glucose level in the past 2–3 months and is an important criterion for assessing blood glucose control. In 2010, the diabetes management guidelines promulgated by the ADA had included HbA1c ≥ 6.5% as one of the diagnostic criteria for T2DM (ADA, 2010). In 2011, WHO also recommended HbA1c as one of the criteria for T2DM diagnosis in areas where conditions are well established (WHO, 2011). As the standardization and consistency of HbA1c testing improved in recent years, HbA1c has also been used as a supplementary diagnostic standard in China (CDS, 2021). The United Kingdom Prospective Diabetes Study (UKPDS) showed that the risk of various complications in T2DM patients was closely related to glycemic control. Each 1% reduction in HbA1c reduced the risk of all diabetes-related endpoints by 21%, the risk of diabetes-related death by 21%, the risk of myocardial infarction by 14%, and the risk of microvascular complications by 37% (Stratton et al., 2000). According to our findings, combining DCHD with conventional treatment could be a beneficial complementary therapy for diabetic patients. In addition, for the combination treatment, we found large heterogeneity in the HbA1c and FBG results, and performed meta-regression and subgroup analysis to explore the source of heterogeneity. The results revealed that age may be one of the reasons affecting the efficacy of DCHD on HbA1c (*p* = 0.023, Adj R^2^ = 53.55%), and DCHD may be more effective in lowering HbA1c in middle-aged and elderly patients. For FBG, different BMI levels may be a source of heterogeneity. DCHD may have a better effect on FBG in obese patients with a BMI greater than 24. Subgroup analysis of 2hPG also found that BMI was one of the factors affecting the efficacy.

In terms of lipid metabolism, the combination treatment had a certain improvement on TC (MD = −0.50 mmol/L, 95%CI: −0.70 to −0.30, *p* < 0.01), TG (MD = −0.44 mmol/L, 95%CI: −0.61 to −0.26, *p* < 0.01) and LDL-C (MD = −0.58 mmol/L, 95%CI: −0.85 to −0.31, *p* < 0.01). This suggested that combined DCHD treatment may be suitable for T2DM patients with abnormal lipid metabolism. Due to the small number of included studies, it is unclear whether DCHD used alone has an improving effect on lipid metabolism.

We also found that the combined DCHD treatment was significantly better than conventional treatment in decreasing HOMA-IR (SMD = −2.04, 95%CI: 3.09 to −0.99, *p* < 0.01), improving HOMA-β (SMD = 2.48, 95%CI: 2.20 to 2.76, *p* < 0.01) and reducing BMI (MD = −1.52 kg/m^2^, 95%CI: −2.55 to −0.49, *p* < 0.01). We found large heterogeneity in HOMA-IR and BMI. Subgroup analysis of HOMA-IR did not reveal a source of heterogeneity. We speculated that the heterogeneity may be related to the large individual differences in HOMA-IR, resulting in the baseline level and improvement degree on HOMA-IR varying widely among study points. It may also be related to measurement bias caused by different insulin detection methods. In addition, the methodological inadequacies of the included studies, such as the lack of blinding and allocation concealment, may also contribute to the heterogeneity. Subgroup analysis of BMI found that different course of disease may be a reason affecting the efficacy of DCHD (total heterogeneity: I^2^ = 82%; subgroup analysis: I^2^ = 59% and 66%, respectively), and DCHD may be more effective in reducing BMI for patients with a course of disease less than 5 years. No significant heterogeneity was found in HOMA-β, suggesting that the results were relatively consistent between individual studies. Due to the small number of included studies, it is unclear whether DCHD used alone has an improving effect on HOMA-IR, HOMA-β and BMI, which remains to be further explored in future research.

Only one study (Cui and Chen 2015) claimed to have performed a placebo control. However, the dosage form and frequency of medications in the experimental and control groups were different, which is likely to break the blindness, so the evidence for DCHD alone vs. placebo is insufficient.

Among the 17 included studies, three studies evaluated the adverse events. One study (Zhao et al., 2016) reported that DCHD had no effect on liver function. One study (Zhang H. S. et al., 2019) reported that combination with DCHD could reduce the incidence of hypoglycemia. One study (Zhang Q. J. et al., 2021) reported four cases of loose stool in the combination treatment group, five cases of abdominal distension and two cases of nausea in the conventional treatment group. The adverse effects were mainly concentrated on the gastrointestinal tract. These adverse effects resolved spontaneously, and no special treatment was given. No serious adverse events were observed. The results showed that, when used correctly, DCHD is relatively safe. The remaining 14 studies did not report adverse events, suggesting that researchers did not pay enough attention to adverse events. Meanwhile, drug safety needs to be evaluated by multiple indicators, such as blood routine, urine routine, stool routine, liver and kidney function, incidence of hypoglycemia and patient self-reported discomfort, so as to fully reflect the drug’s impact on human safety. However, the literature included in this study only reported liver function, the incidence of hypoglycemia and gastrointestinal adverse effects, so the indicators involved were not comprehensive enough. Therefore, more high-quality studies and more comprehensive indicators are needed to further confirm its safety in the future.

It is worth noting that although the literature included in this study showed DCHD did not increase the occurrence of adverse effects, the clinical application of DCHD still needs to be considered comprehensively. DCHD may have potential adverse effects or toxicity if improperly applied. DCHD has the functions of soothing liver and relieving depression, clearing stomach and purging heat, and is mainly used to treat the syndrome of heat stagnation in liver and stomach. In DCHD, Chinese Thorowax Root (*Chaihu*, Bupleurum falcatum L.), Baical Skullcap Root (*Huangqin*, Scutellaria baicalensis Georgi) and Rhubarb (*Dahuang*, Rheum palmatum L.) are cold in nature. If the dose is too large or applied to patients with weak constitution, it may injure the yang qi of spleen and stomach, causing abdominal distension, diarrhea, loose stool or epigastric discomfort. Therefore, the clinical application of DCHD cannot be separated from the principle of syndrome differentiation and treatment, and it should be selected reasonably according to the pathogenesis and constitution. Meanwhile, the dosage of each herb should be adjusted according to the condition to reduce the occurrence of adverse effects. At present, there is no complete report on the toxicity of DCHD. Pharmacological studies have shown that Pinellia Tuber (*Banxia*, Pinellia ternata (Thunb.) Makino) is a poisonous herb because of its alkaloids, lectins and toxic raphides of calcium oxalate (Ji et al., 2014; Yu H. L. et al., 2015; Mao, 2018). These components may cause mucosal irritation, liver and kidney toxicity, and pregnancy toxicity (Xu et al., 2013; Xie et al., 2016; Xu et al., 2017; Xu et al., 2018; Jin et al., 2019). Therefore, in order to ensure safety, Pinellia Tuber, which has been processed strictly, should be used in DCHD. Through processing, the structure of calcium oxalate raphides can be destroyed, and the lectin protein will also be denatured and inactivated, thus achieving the effect of detoxification (Yu H. et al., 2015; Jin et al., 2019; Jiang and Li, 2021). Studies have also found that the use of processed Pinellia Tuber could greatly reduce the occurrence of poisoning events (Chen et al., 2020). Due to different processing excipients, Pinellia Tuber has different tendencies in efficacy and clinical application (Chen et al., 2020; Jiang and Li, 2021). Clinically, the dose of Pinellia Tuber should be reasonably determined based on the condition and the results of toxicology studies, and attention should be paid to herb concerted application, so as to exert the therapeutic effect and reduce the toxicity. The toxicity of DCHD still needs further pharmacological and toxicological studies to explore.

Funnel plot and Egger’s test were performed on HBA1c, FBG and 2hPG, and no publication bias was detected, indicating that the results have certain reliability.

### Study on the internal possible mechanism

TCM formulas have played a unique role in preventing and treating T2DM with their multi-component and multi-target advantages. Pharmacological studies have shown that DCHD can improve glucose and lipid metabolism, increase antioxidant enzymes activity, reduce reactive oxygen species (ROS), superoxide dismutase (SOD) and malondialdehyde (MDA) level, and up-regulate the expression of pancreatic duodenal homeobox-1 (PDX-1) and MaFA mRNA in pancreatic tissue (Cui Y. R. et al., 2020). That is, it can protect pancreatic β cells by inhibiting oxidative stress. It can also increase glucose transport and improve insulin resistance by modulating the activity of the insulin receptor substrate-1/phosphor inositide-3-kinase/protein kinase B (IRS-1/PI3K/Akt) pathway in liver tissue (Hou et al., 2020). Another study found that DCHD can significantly reduce blood sugar and cholesterol levels, increase HDL-C levels, improve glucose homeostasis and insulin resistance, reduce hepatic fat deposition and decrease total fat content. The underlying mechanism may be related to regulating the expression of adiponectin and leptin genes in adipose tissue, inhibiting the proliferation and differentiation of adipose tissue, and balancing intestinal flora (Hussain et al., 2016). Using network pharmacology to explore the mechanism of DCHD in preventing and treating T2DM, it was found that DCHD may be closely related to tumor necrosis factor (TNF) signaling pathway, PI3K/Akt signaling pathway, p53 signaling pathway and apoptotic signaling pathway, and then play a role in regulating inflammatory response, improving insulin resistance and inhibiting pancreatic β-cell apoptosis (Ren et al., 2020; Zhao and Xu, 2020; Zhang S. W. et al., 2021). In conclusion, the internal reason for DCHD to regulate glucose and lipid metabolism, reduce insulin resistance and improve islet cell function may be related to inhibiting oxidative stress, regulating inflammatory response, inhibiting islet cell apoptosis, modulating insulin signal transduction, regulating adiponectin and leptin gene expression, and balancing intestinal flora.

### Limitation of this study

Although we have tried our best to use standard analytical methods, this study still has some limitations. Firstly, the methodological quality of the included studies was not high, and most studies had unclear randomization methods, lacked blinding, and did not report allocation concealment. Only one study used a placebo control, but there was a risk of unblinding. All of these may lead to a certain risk of bias. Secondly, the included studies were all single-center and small-sample, which may lack representativeness. Thirdly, some studies did not fully report research characteristics such as course of disease, treatment duration and BMI, so the selection of analysis methods for heterogeneity and the exploration of the dominant population were limited. The presence or absence of comorbidities was not reported in most studies, so the subgroup analysis on comorbidities could not be performed. Meanwhile, most of the studies were poorly standardized in reporting dropped cases. Fourthly, the included studies are all Chinese literature, which may have ethnic and regional limitations. None of the included studies were registered and no study protocol was obtained. For positive results in China are more likely to be published, there may be potential publication bias. Finally, adverse events were not reported in most studies, making it difficult to evaluate safety objectively. Therefore, there is still uncertainty about the efficacy of DCHD in the T2DM treatment.

### Implications for clinical practice and future research

Based on the above findings and limitations, the following suggestions are provided for future research and practice: Firstly, improve study protocol rigor and strengthen quality control, with particular attention to the correct implementation of center randomization, allocation concealment and blinding. Placebo control should be used reasonably to eliminate the influence of psychological factors, so as to evaluate the true efficacy and adverse effects of the experimental drug. Secondly, carry out multi-center studies and calculate the sample size reasonably to make the study results more reliable and representative. Thirdly, the report of RCTs should be conducted strictly following the Consolidated Standards of Reporting Trials (CONSORT) statement, with particular emphasis on reporting the age, course of disease, treatment duration, and presence or absence of comorbidities, so as to explore the source of heterogeneity through statistical analysis and further analyze the dominant population. Fourthly, clinical trial registration should be carried out before starting, and both positive and negative results should be reported truthfully to ensure transparency of information and reduce publication bias. Finally, pay attention to observing and monitoring adverse events and establish strict adverse events handling and reporting procedures.

## Conclusion

In summary, we found that the combination with DCHD in the T2DM treatment has more advantages than conventional treatment alone, which can further regulate the glucose and lipid metabolism, reduce insulin resistance, improve islet function and lower BMI. DCHD used alone can also play a certain role in regulating blood glucose, but the current evidence is insufficient to clarify the effect of DCHD alone on lipid metabolism, insulin resistance, islet function and BMI. Meanwhile, DCHD is relatively safe. This indicates that DCHD may have a positive effect on T2DM. However, given the limited number of included studies, small sample size and poor methodological quality, the evidence of this study remain uncertain and the results should be interpreted and applied with caution. In the T2DM treatment, clinical decisions still need to be made by considering the patient’s overall situation. In the future, more high-quality, large-sample, multi-center, randomized, double-blind and placebo-controlled studies are still needed to provide more reliable evidence for the clinical application of DCHD.

## Data Availability

The original contributions presented in the study are included in the article/Supplementary Material, further inquiries can be directed to the corresponding authors.

## Glossary

2hPG: 2-h postprandial glucose
ADA: American Diabetes Association
BMI: body mass index
CDS: Chinese Diabetes Society
CG: control group interventions
CHiCTR: Chinese Clinical Trial Registry
CI: confidence interval
CNKI: China National Knowledge Infrastructure
CONSORT: Consolidated Standards of Reporting Trials
DCHD: dachaihu decoction
FBG: fasting blood glucose
GRADEGrading of Recommendations Assessment: Grading of Recommendations AssessmentDevelopment and Evaluation
HbA1c: glycated hemoglobin
HDL-C: high-density lipoprotein cholesterol
HOMA-IR: homeostasis model assessment of insulin resistance
HOMA-β: homeostasis model assessment of beta-cell function
IRS-1/PI3K/Akt: insulin receptor substrate-1/phosphor inositide-3-kinase/protein kinase B
LDL-C: low-density lipoprotein cholesterol
MD: mean difference
MDA: malondialdehyde
NR: not reported
NSD: no significant difference
PDX-1: pancreatic duodenal homeobox-1
PRISMA: the Preferred Reporting Items for Systematic Review and Meta-Analysis
PROSPERO: International Prospective Register of Systematic Reviews
RCTs: randomized controlled trials
ROS: reactive oxygen species
RR: relative risk
SMD: standardized mean difference
SOD: superoxide dismutase
T2DM: type 2 diabetes mellitus
TCM: traditional Chinese medicine
TC: total cholesterol
TG: triglyceride
TNF: tumor necrosis factor
UKPDS: United Kingdom Prospective Diabetes Study
VIP: China Science and Technology Journal Database
WHO: World Health Organization.
