# Supplementary material for: The efficacy and safety of dachaihu decoction in the treatment of type 2 diabetes mellitus: A systematic review and meta-analysis
Source: Front Pharmacol. 2022 Aug 8;13:918681. doi: 10.3389/fphar.2022.918681 (PMC9393237; doi:10.3389/fphar.2022.918681)
Supplement: Supplementary file 2 [file Table2.DOCX]

**1. More details about the included formulations**

| **Study** | **Formulation** | **Source** | **Species, concentration** | **Quality control reported? (Y/****N)** | **Chemical analysis reported? (Y/N)** |
| --- | --- | --- | --- | --- | --- |
| Liu (2002) | Metformin | China Resources Double-Crane Pharmaceutical Co.,Ltd. | Not reported | Y- Product Lot Number: 990825 | N |
|  | modified Dachaihu Decoction | Prepared by Department of Pharmacy, China-Japan Friendship Hospital (2002) | 1. Chaihu: root of Bupleurum falcatum L.;  2. Huangqin: root of Scutellaria baicalensis Georgi;  3. Huanglian: rhizome of Coptis chinensis Franch.;  4. Banxia: tuber of Pinellia ternata (Thunb.) Makino;  5. Zhishi: fruit of Citrus aurantium L.;  6. Dahuang: rhizome and root of Rheum palmatum L.;  7. Wumei: fruit of Prunus mume (Siebold) Siebold & Zucc.). | N- Granules to be taken with water. | N |
| Shen and Wu (2007) | Novoline | Novo Nordisk (China) Pharmaceuticals Co.,Ltd. | Not reported | N | N |
|  | modified Dachaihu Decoction | Prepared by Shen and Wu (2007) | Not reported | N- Decoct with water, divide into 3 bags, 100ml each. | N |
| Cui and Chen (2015) | Placebo | Prepared by Preparation room, Luanping Hospital of Traditional Chinese Medicine, Hebei Province (2015) | Ingredients: Starch, Caramel Color | N | N |
|  | modified Dachaihu Decoction | Prepared by Beijing Kangrentang Pharmaceutical Co., Ltd. (2007) | 1. Chaihu: root of Bupleurum falcatum L., 15g;  2. Huangqin: root of Scutellaria baicalensis Georgi, 15g;  3. Huanglian: rhizome of Coptis chinensis Franch., 10g;  4. Banxia: tuber of Pinellia ternata (Thunb.) Makino, 10g;  5. Baishao: root of Paeonia lactiflora Pall., 25g;  6. Dahuang: rhizome and root of Rheum palmatum L., 10g;  7. Zhishi: fruit of Citrus aurantium L., 15g;  8. Gegen: root of Pueraria montana var. thomsonii (Benth.) M.R.Almeida, 20g;  9. Shigao: ore of Fibrosum Gypsum, 30g;  10. Dilong: dry worm of Pheretima, 15g;  11. Taoren: seed of Prunus persica (L.) Batsch, 15g;  12. Baihuasheshecao: whole plant of Scleromitrion diffusum (Willd.) R.J.Wang, 15g;  13. Zhimu: rhizome of Anemarrhena asphodeloides Bunge, 15g;  14. Danshen: root and rhizome of Salvia miltiorrhiza Bunge, 30g;  15. Xuanshen: root of Scrophularia ningpoensis Hemsl., 15g;  16. Tianhuafen: root of Trichosanthes kirilowii Maxim., 30g;  17. Huangqi: root of Astragalus mongholicus Bunge, 20g;  18. Dangshen: root of Codonopsis pilosula (Franch.) Nannf., 15g;  19. Juhua: capitulum of Chrysanthemum indicum L., 15g;  20. Shengmuli: shell of Ostreae Concha, 30g;  21. Shijueming: shell of Haliotidis Concha, 15g;  22. Baizhu: rhizome of Atractylodes macrocephala Koidz., 15g;  23. Longdancao: root and rhizome of Gentiana scabra Bunge, 10g. | N- Granules to be taken with water. | N |
| Zhao et al. (2016) | Metformin | Sino-American Shanghai Squibb Pharmaceuticals Ltd. | Not reported | N | N |
|  | modified Dachaihu Decoction | Prepared by Zhao et al. (2016) | 1. Chaihu: root of Bupleurum falcatum L.;  2. Huangqin: root of Scutellaria baicalensis Georgi;  3. Dahuang: rhizome and root of Rheum palmatum L.;  4. Zhishi: fruit of Citrus aurantium L.;  5. Banxia: tuber of Pinellia ternata (Thunb.) Makino;  6. Baishao: root of Paeonia lactiflora Pall.;  7. Longdancao: root and rhizome of Gentiana scabra Bunge;  8. Shanzha: fruit of Crataegus pinnatifida Bunge;  9. Yinchen: aerial parts of Artemisia capillaris Thunb.;  10. Zexie: bulb of Alisma plantago-aquatica L.;  11. Danshen: root and rhizome of Salvia miltiorrhiza Bunge. | N- Decoct with water. | N |
| Zhang et al. (2018) | Liraglutide | Novo Nordisk (China) Pharmaceuticals Co.,Ltd. | Specification: 3ml, 18mg | Y- National medicine permission number: J20160037. | N |
|  | modified Dachaihu Decoction | Prepared by Zhang et al. (2018) | 1. Chaihu: root of Bupleurum falcatum L., 20g;  2. Huangqin: root of Scutellaria baicalensis Georgi, 20g;  3. Dahuang: rhizome and root of Rheum palmatum L., 15g;  4. Huanglian: rhizome of Coptis chinensis Franch., 15g;  5. Zhishi: fruit of Citrus aurantium L., 15g;  6. Gualou: root of Trichosanthes kirilowii Maxim., 15g;  7. Banxia: tuber of Pinellia ternata (Thunb.) Makino, 10g;  8. Baishao: root of Paeonia lactiflora Pall., 10g;  9. Gancao: root and rhizome of Glycyrrhiza glabra L., 6g. | N- Decoct with water and take the supernatant. | N |
| Zhang (2018) | Hypoglycemic agents | Not reported | Not reported | N | N |
|  | Dachaihu Decoction | Prepared by Zhang (2018) | 1. Huangqin: root of Scutellaria baicalensis Georgi, 9g;  2. Banxia: tuber of Pinellia ternata (Thunb.) Makino, 8g;  3. Baishao: root of Paeonia lactiflora Pall., 9g;  4. Zhishi: fruit of Citrus aurantium L., 9g;  5. Shengjiang: rhizome of Zingiber officinale Roscoe, 16g;  6. Dahuang: rhizome and root of Rheum palmatum L., 7g;  7. Chaihu: root of Bupleurum falcatum L., 12g;  8. Dazao: fruit of Ziziphus jujuba Mill., 5 pieces. | N- Decoct with 400ml water, boil on high heat, then decoct with low heat for 20 minutes, take the supernatant. | N |
| Li et al. (2018) | Metformin | Beijing Shengyong Pharmaceutical Co.,Ltd. | Specification: 0.25g | Y- Product Lot Number: 13070001, 14030106, 15030107 | N |
|  | modified Dachaihu Decoction | Prepared by Li et al. (2018) | 1. Chaihu: root of Bupleurum falcatum L., 10g;  2. Zhishi: fruit of Citrus aurantium L., 15g;  3. Huangqin: root of Scutellaria baicalensis Georgi, 15g;  4. Dahuang: rhizome and root of Rheum palmatum L., 8g;  5. Huanglian: rhizome of Coptis chinensis Franch., 15g;  6. Baishao: root of Paeonia lactiflora Pall., 15g;  7. Zhimu: rhizome of Anemarrhena asphodeloides Bunge, 15g;  8. Ganjiang: rhizome of Zingiber officinale Roscoe, 3g;  9. Gegen: root of Pueraria montana var. thomsonii (Benth.) M.R.Almeida;  10. Tianhuafen: root of Trichosanthes kirilowii Maxim.;  11. Gualouzi: seed of Trichosanthes kirilowii Maxim.;  12. Banxia: tuber of Pinellia ternata (Thunb.) Makino. | N- Decoct with water. | N |
| Zhang et al. (2019) | Gansulin R | Tonghua Dongbao Pharmaceutical Co.,Ltd. | Not reported | Y- National medicine permission number: S20020092. | N |
|  | Insulin glargine | Sanofi (Beijing) Pharmaceuticals Co.,Ltd. | Not reported | Y- National medicine permission number: J20140052. | N |
|  | Metformin | Beijing Lilinghengtai medicine limited company | Not reported | Y- National medicine permission number: H11021560. | N |
|  | Dachaihu Decoction | Prepared by Zhang et al. (2019) | 1. Chaihu: root of Bupleurum falcatum L., 12g;  2. Dahuang: rhizome and root of Rheum palmatum L., 8g;  3. Huangqin: root of Scutellaria baicalensis Georgi, 12g;  4. Banxia: tuber of Pinellia ternata (Thunb.) Makino, 9g;  5. Zhishi: fruit of Citrus aurantium L., 12g;  6. Chishao: root of Paeonia lactiflora Pall., 12g;  7. Dazao: fruit of Ziziphus jujuba Mill.,10g;  8. Shengjiang: rhizome of Zingiber officinale Roscoe, 10g. | N- Firstly, add 300mL water, soak for 30min, boil on high heat and simmer with low heat for 30min, take 100mL supernatant. Secondly, add 200mL water, boil on high heat, decoct with water for 20 minutes, take 100mL supernatant. Mix the two supernatants. | N |
| Gao (2019) | Liraglutide | Novo Nordisk (China) Pharmaceuticals Co.,Ltd. | Not reported | Y- National medicine permission number: J20160037. | N |
|  | modified Dachaihu Decoction | Prepared by Gao (2019) | 1. Chaihu: root of Bupleurum falcatum L., 20g;  2. Baishao: root of Paeonia lactiflora Pall., 10g;  3. Huangqin: root of Scutellaria baicalensis Georgi, 20g;  4. Banxia: tuber of Pinellia ternata (Thunb.) Makino, 10g;  5. Zhishi: fruit of Citrus aurantium L., 15g;  6. Dahuang: rhizome and root of Rheum palmatum L., 10g;  7. Gancao: root and rhizome of Glycyrrhiza glabra L., 6g;  8. Yujin: root tuber of Curcuma longa L., 15g. | N- Decoct with water. | N |
| Li (2019) | Liraglutide | Novo Nordisk (China) Pharmaceuticals Co.,Ltd. | Not reported | N | N |
|  | modified Dachaihu Decoction | Prepared by Li (2019) | 1. Chaihu: root of Bupleurum falcatum L., 20g;  2. Huangqin: root of Scutellaria baicalensis Georgi, 20g;  3. Huanglian: rhizome of Coptis chinensis Franch., 15g;  4. Zhishi: fruit of Citrus aurantium L., 15g;  5. Dahuang: rhizome and root of Rheum palmatum L., 15g;  6. Gualou: root of Trichosanthes kirilowii Maxim., 15g;  7. Banxia: tuber of Pinellia ternata (Thunb.) Makino, 10g;  8. Baishao: root of Paeonia lactiflora Pall., 10g;  9. Gancao: root and rhizome of Glycyrrhiza glabra L., 6g. | N- Decoct with water. | N |
| part-1 Ji and Che (2020) | Metformin | Sino-American Shanghai Squibb Pharmaceuticals Ltd. | Specification: 0.5g | Y- National medicine permission number: H20023370. | N |
|  | modified Dachaihu Decoction | Prepared by Ji and Che (2020) | 1. Chaihu: root of Bupleurum falcatum L., 10g;  2. Baishao: root of Paeonia lactiflora Pall., 15g;  3. Huangqin: root of Scutellaria baicalensis Georgi, 10g;  4. Banxia: tuber of Pinellia ternata (Thunb.) Makino, 10g;  5. Zhishi: fruit of Citrus aurantium L., 10g;  6. Dahuang: rhizome and root of Rheum palmatum L., 6g;  7. Jueming: seed of Senna tora (L.) Roxb., 15g;  8. Cangzhu: rhizome of Atractylodes lancea (Thunb.) DC., 15g;  9. Xuanshen: root of Scrophularia ningpoensis Hemsl., 20g;  10. Yujin: root tuber of Curcuma longa L., 15g. | N- Decoct with water to 400 mL. | N |
| part-2 Ji and Che (2020) | Metformin | Sino-American Shanghai Squibb Pharmaceuticals Ltd. | Specification: 0.5g | Y- National medicine permission number: H20023370. | N |
|  | modified Dachaihu Decoction | Prepared by Ji and Che (2020) | 1. Chaihu: root of Bupleurum falcatum L., 10g;  2. Baishao: root of Paeonia lactiflora Pall., 15g;  3. Huangqin: root of Scutellaria baicalensis Georgi, 10g;  4. Banxia: tuber of Pinellia ternata (Thunb.) Makino, 10g;  5. Zhishi: fruit of Citrus aurantium L., 10g;  6. Dahuang: rhizome and root of Rheum palmatum L., 6g;  7. Jueming: seed of Senna tora (L.) Roxb., 15g;  8. Cangzhu: rhizome of Atractylodes lancea (Thunb.) DC., 15g;  9. Xuanshen: root of Scrophularia ningpoensis Hemsl., 20g;  10. Yujin: root tuber of Curcuma longa L., 15g. | N- Decoct with water to 400 mL. | N |
| Wang (2020) | Metformin | Not reported | Not reported | N | N |
|  | modified Dachaihu Decoction | Prepared by Wang (2020) | 1. Chaihu: root of Bupleurum falcatum L., 20g;  2. Huanglian: rhizome of Coptis chinensis Franch., 15g;  3. Huangqin: root of Scutellaria baicalensis Georgi, 20g;  4. Zhishi: fruit of Citrus aurantium L., 15g;  5. Dahuang: rhizome and root of Rheum palmatum L., 15g;  6. Gualou: root of Trichosanthes kirilowii Maxim., 15g;  7. Baishao: root of Paeonia lactiflora Pall., 10g;  8. Banxia: tuber of Pinellia ternata (Thunb.) Makino, 10g;  9. Gancao: root and rhizome of Glycyrrhiza glabra L., 6g. | N- Decoct twice with water, taking 150 mL supernatant each time. Mix the two supernatants. | N |
| Bao et al. (2020) | Exenatide | Not reported | Not reported | N | N |
|  | Dachaihu Decoction | Prepared by Bao et al. (2020) | 1. Chaihu: root of Bupleurum falcatum L., 15g;  2. Baishao: root of Paeonia lactiflora Pall., 10g;  3. Huangqin: root of Scutellaria baicalensis Georgi, 10g;  4. Banxia: tuber of Pinellia ternata (Thunb.) Makino, 10g;  5. Zhishi: fruit of Citrus aurantium L., 10g;  6. Shengjiang: rhizome of Zingiber officinale Roscoe, 10g;  7. Dahuang: rhizome and root of Rheum palmatum L., 6g;  8. Dazao: fruit of Ziziphus jujuba Mill., 4 pieces. | N- Decoct with water, take 400 mL supernatant. | N |
| Chang (2020) | Insulin glargine | Not reported | Not reported | N | N |
|  | modified Dachaihu Decoction | Prepared by Chang (2020) | 1. Chaihu: root of Bupleurum falcatum L., 15g;  2. Baishao: root of Paeonia lactiflora Pall., 9g;  3. Huangqin: root of Scutellaria baicalensis Georgi, 9g;  4. Banxia: tuber of Pinellia ternata (Thunb.) Makino, 9g;  5. Zhishi: fruit of Citrus aurantium L., 9g;  6. Dahuang: rhizome and root of Rheum palmatum L., 9g;  7. Gancao: root and rhizome of Glycyrrhiza glabra L., 6g. | N- Decoct with water. | N |
| Zou et al. (2021) | Liraglutide | Novo Nordisk (China) Pharmaceuticals Co.,Ltd. | Specification: 3ml, 18mg | Y- National medicine permission number: J20160037. | N |
|  | Insulin glargine | Zhuhai United Laboratories Co.,Ltd. | Specification: 3ml, 300 U | Y- National medicine permission number: S20173001. | N |
|  | Dachaihu Decoction | Prepared by Zou et al. (2021) | 1. Chaihu: root of Bupleurum falcatum L., 15g;  2. Dahuang: rhizome and root of Rheum palmatum L., 6g;  3. Zhishi: fruit of Citrus aurantium L., 9g;  4. Huangqin: root of Scutellaria baicalensis Georgi, 9g;  5. Banxia: tuber of Pinellia ternata (Thunb.) Makino, 9g;  6. Baishao: root of Paeonia lactiflora Pall., 9g;  7. Dazao: fruit of Ziziphus jujuba Mill., 4 pieces  8. Shengjiang: rhizome of Zingiber officinale Roscoe, 15g. | N- Decoct with water to 300 mL. | N |
| Duan (2021) | Conventional hypoglycemic drugs | Not reported | Not reported | N | N |
|  | modified Dachaihu Decoction | Prepared by Duan (2021) | 1. Chaihu: root of Bupleurum falcatum L., 10g;  2. Dahuang: rhizome and root of Rheum palmatum L., 10g;  3. Banxia: tuber of Pinellia ternata (Thunb.) Makino, 15g;  4. Huangqin: root of Scutellaria baicalensis Georgi, 15g;  5. Baishao: root of Paeonia lactiflora Pall., 15g;  6. Zhishi: fruit of Citrus aurantium L., 15g;  7. Dazao: fruit of Ziziphus jujuba Mill., 10g;  8. Shengjiang: rhizome of Zingiber officinale Roscoe, 6g;  9. Gegen: root of Pueraria montana var. thomsonii (Benth.) M.R.Almeida, 20g;  10. Danshen: root and rhizome of Salvia miltiorrhiza Bunge, 30g;  11. Taoren: seed of Prunus persica (L.) Batsch, 15g;  12. Zhimu: rhizome of Anemarrhena asphodeloides Bunge, 15g;  13. Dilong: dry worm of Pheretima, 15g;  14. Xuanshen: root of Scrophularia ningpoensis Hemsl., 15g;  15. Tianhuafen: root of Trichosanthes kirilowii Maxim., 30g  16. Huangqi: root of Astragalus mongholicus Bunge, 20g;  17. Dangshen: root of Codonopsis pilosula (Franch.) Nannf., 15g;  18. Juhua: capitulum of Chrysanthemum indicum L., 15g;  19. Shengmuli: shell of Ostreae Concha, 30g;  20. Shijueming: shell of Haliotidis Concha, 15g;  21. Baizhu: rhizome of Atractylodes macrocephala Koidz., 15g;  22. Longdancao: root and rhizome of Gentiana scabra Bunge, 10g. | N- Decoct with water and take the supernatant. | N |
| Zhang et al. (2021) | Conventional hypoglycemic drugs | Not reported | Not reported | N | N |
|  | modified Dachaihu Decoction | Prepared by Pharmacy of Guang'anmen Hospital, China Academy Of Chinese Medical Sciences(2021) | 1. Chaihu: root of Bupleurum falcatum L., 10g;  2. Huangqin: root of Scutellaria baicalensis Georgi, 10g;  3. Zhishi: fruit of Citrus aurantium L., 10g;  4. Banxia: tuber of Pinellia ternata (Thunb.) Makino, 9g;  5. Baishao: root of Paeonia lactiflora Pall., 20g;  6. Cangzhu: rhizome of Atractylodes lancea (Thunb.) DC., 15g;  7. Huangbo: bark of Phellodendron amurense Rupr., 15g;  8. Yiyiren: seed of Coix lacryma-jobi L., 30g;  9. Tufuling: rhizome of Smilax glabra Roxb., 30g;  10. Cansha: faeces of Bombycis, 30g;  11. Shancigu: pseudobulb of Cremastra appendiculata (D.Don) Makino, 10g;  12. Chuanniuxi: root of Cyathula officinalis K.C.Kuan, 15g. | N- Granules to be taken with water. | N |

**2.** **Chemical characterisation of Dachaihu Decoction**

The main active components of DCHD measured by high performance liquid chromatography include paeoniflorin, naringin, hesperidin, neohesperidin, baicalin, baicalein and saikosaponin A (Li et al., 2006; Liu, 2014).

Li, C.Y., Chiu, C.H., Huang, H.S., Lin, C.H., and Wu, T.S. (2006). High-Performance Liquid Chromatographic Method for Simultaneous Quantification of Eight Major Biologically Active Ingredients in 'Da-Chai-Hu-Tang' Preparation. Biomed Chromatogr. 20(4), 305-308. doi: 10.1002/bmc.572.

Liu, X.Q. (2014). Studies on HPLC Fingerprints of Da Chaihu Decoction and Guifu Dihuang Prescription. [Master's thesis]. Beijing: Academy of Military Sciences.
