# Supplementary material for: The efficacy and safety of dachaihu decoction in the treatment of type 2 diabetes mellitus: A systematic review and meta-analysis
Source: Front Pharmacol. 2022 Aug 8;13:918681. doi: 10.3389/fphar.2022.918681 (PMC9393237; doi:10.3389/fphar.2022.918681)
Supplement: Supplementary file 3 [file DataSheet1.docx]

**Contents Page**

[Supplementary Material S1. PRISMA 2020 checklist 1](#_Toc100159944)

[Supplementary Material S2. Database and Search Strategies 4](#_Toc100159945)

[Supplementary Material S3. The procedure for preliminary screening of the literature 14](#_Toc100159946)

[Supplementary Material S4. Literature excluded after reading the full text and reasons 15](#_Toc100159947)

[Supplementary Material S5. Meta-regression analysis of HbA1c and FBG 20](#_Toc100159948)

[Supplementary Material S6. Subgroup analysis of HbA1c for DCHD combined with conventional treatment vs. conventional treatment 22](#_Toc100159949)

[Supplementary Material S7. Sensitivity analysis 23](#_Toc100159950)

[Supplementary Material S8. Subgroup analysis of HbA1c for DCHD vs. conventional treatment 27](#_Toc100159951)

[Supplementary Material S9. Subgroup analysis of FBG for DCHD combined with conventional treatment vs. conventional treatment 28](#_Toc100159952)

[Supplementary Material S10. Subgroup analysis of FBG for DCHD vs. conventional treatment 29](#_Toc100159953)

[Supplementary Material S11. Subgroup analysis of 2hPG for DCHD combined with conventional treatment vs. conventional treatment 30](#_Toc100159954)

[Supplementary Material S12. Subgroup analysis of 2hPG for DCHD vs. conventional treatment 31](#_Toc100159955)

[Supplementary Material S13. Subgroup analysis of TC for DCHD combined with conventional treatment vs. conventional treatment 32](#_Toc100159956)

[Supplementary Material S14. Subgroup analysis of TG for DCHD combined with conventional treatment vs. conventional treatment 33](#_Toc100159957)

[Supplementary Material S15. Subgroup analysis of HOMA-IR for DCHD combined with conventional treatment vs. conventional treatment 34](#_Toc100159958)

[Supplementary Material S16. Subgroup analysis of BMI for DCHD combined with conventional treatment vs. conventional treatment 35](#_Toc100159959)

[Supplementary Material S17. Egger’s test of HBA1c, FBG and 2hPG 36](#_Toc100159960)

[Supplementary Material S18. Assessment of evidence quality for each outcome 37](#_Toc100159961)

# Supplementary Material S1. PRISMA 2020 checklist

| **Section and Topic** | **Item #** | **Checklist item** | **Location where item is reported** |
| --- | --- | --- | --- |
| **TITLE** | | |  |
| Title | 1 | Identify the report as a systematic review. | P1 |
| **ABSTRACT** | | |  |
| Abstract | 2 | See the PRISMA 2020 for Abstracts checklist. | P1-2 |
| **INTRODUCTION** | | |  |
| Rationale | 3 | Describe the rationale for the review in the context of existing knowledge. | P2-3 |
| Objectives | 4 | Provide an explicit statement of the objective(s) or question(s) the review addresses. | P3 |
| **METHODS** | | |  |
| Eligibility criteria | 5 | Specify the inclusion and exclusion criteria for the review and how studies were grouped for the syntheses. | P3-4 |
| Information sources | 6 | Specify all databases, registers, websites, organisations, reference lists and other sources searched or consulted to identify studies. Specify the date when each source was last searched or consulted. | P3 |
| Search strategy | 7 | Present the full search strategies for all databases, registers and websites, including any filters and limits used. | Supplementary material 2 |
| Selection process | 8 | Specify the methods used to decide whether a study met the inclusion criteria of the review, including how many reviewers screened each record and each report retrieved, whether they worked independently, and if applicable, details of automation tools used in the process. | P4 |
| Data collection process | 9 | Specify the methods used to collect data from reports, including how many reviewers collected data from each report, whether they worked independently, any processes for obtaining or confirming data from study investigators, and if applicable, details of automation tools used in the process. | P4 |
| Data items | 10a | List and define all outcomes for which data were sought. Specify whether all results that were compatible with each outcome domain in each study were sought (e.g. for all measures, time points, analyses), and if not, the methods used to decide which results to collect. | P4 |
|  | 10b | List and define all other variables for which data were sought (e.g. participant and intervention characteristics, funding sources). Describe any assumptions made about any missing or unclear information. | P3-4 |
| Study risk of bias assessment | 11 | Specify the methods used to assess risk of bias in the included studies, including details of the tool(s) used, how many reviewers assessed each study and whether they worked independently, and if applicable, details of automation tools used in the process. | P4-5 |
| Effect measures | 12 | Specify for each outcome the effect measure(s) (e.g. risk ratio, mean difference) used in the synthesis or presentation of results. | P5 |
| Synthesis methods | 13a | Describe the processes used to decide which studies were eligible for each synthesis (e.g. tabulating the study intervention characteristics and comparing against the planned groups for each synthesis (item #5)). | P5 |
|  | 13b | Describe any methods required to prepare the data for presentation or synthesis, such as handling of missing summary statistics, or data conversions. | P5 |
|  | 13c | Describe any methods used to tabulate or visually display results of individual studies and syntheses. | P5 |
|  | 13d | Describe any methods used to synthesize results and provide a rationale for the choice(s). If meta-analysis was performed, describe the model(s), method(s) to identify the presence and extent of statistical heterogeneity, and software package(s) used. | P5 |
|  | 13e | Describe any methods used to explore possible causes of heterogeneity among study results (e.g. subgroup analysis, meta-regression). | P5 |
|  | 13f | Describe any sensitivity analyses conducted to assess robustness of the synthesized results. | P5 |
| Reporting bias assessment | 14 | Describe any methods used to assess risk of bias due to missing results in a synthesis (arising from reporting biases). | P5 |
| Certainty assessment | 15 | Describe any methods used to assess certainty (or confidence) in the body of evidence for an outcome. | P5 |
| **RESULTS** | | |  |
| Study selection | 16a | Describe the results of the search and selection process, from the number of records identified in the search to the number of studies included in the review, ideally using a flow diagram. | P5, figure 1 |
|  | 16b | Cite studies that might appear to meet the inclusion criteria, but which were excluded, and explain why they were excluded. | Supplementary material 4 |
| Study characteristics | 17 | Cite each included study and present its characteristics. | P5-6, table 1, table 2 |
| Risk of bias in studies | 18 | Present assessments of risk of bias for each included study. | P6, figure 2 |
| Results of individual studies | 19 | For all outcomes, present, for each study: (a) summary statistics for each group (where appropriate) and (b) an effect estimate and its precision (e.g. confidence/credible interval), ideally using structured tables or plots. | P12-20 |
| Results of syntheses | 20a | For each synthesis, briefly summarise the characteristics and risk of bias among contributing studies. | P12-20 |
|  | 20b | Present results of all statistical syntheses conducted. If meta-analysis was done, present for each the summary estimate and its precision (e.g. confidence/credible interval) and measures of statistical heterogeneity. If comparing groups, describe the direction of the effect. | P12-20 |
|  | 20c | Present results of all investigations of possible causes of heterogeneity among study results. | P12-20 |
|  | 20d | Present results of all sensitivity analyses conducted to assess the robustness of the synthesized results. | P12-20 |
| Reporting biases | 21 | Present assessments of risk of bias due to missing results (arising from reporting biases) for each synthesis assessed. | P21-22 |
| Certainty of evidence | 22 | Present assessments of certainty (or confidence) in the body of evidence for each outcome assessed. | P22 |
| **DISCUSSION** | | |  |
| Discussion | 23a | Provide a general interpretation of the results in the context of other evidence. | P22-25 |
|  | 23b | Discuss any limitations of the evidence included in the review. | P25 |
|  | 23c | Discuss any limitations of the review processes used. | P25 |
|  | 23d | Discuss implications of the results for practice, policy, and future research. | P25-26 |
| **OTHER INFORMATION** | | |  |
| Registration and protocol | 24a | Provide registration information for the review, including register name and registration number, or state that the review was not registered. | P3,CRD42021296718 |
|  | 24b | Indicate where the review protocol can be accessed, or state that a protocol was not prepared. | CRD42021296718 |
|  | 24c | Describe and explain any amendments to information provided at registration or in the protocol. | - |
| Support | 25 | Describe sources of financial or non-financial support for the review, and the role of the funders or sponsors in the review. | P26 |
| Competing interests | 26 | Declare any competing interests of review authors. | P26 |
| Availability of data, code and other materials | 27 | Report which of the following are publicly available and where they can be found: template data collection forms; data extracted from included studies; data used for all analyses; analytic code; any other materials used in the review. | P26 |

*From:* Page MJ, McKenzie JE, Bossuyt PM, Boutron I, Hoffmann TC, Mulrow CD, et al. The PRISMA 2020 statement: an updated guideline for reporting systematic reviews. BMJ 2021;372:n71. doi: 10.1136/bmj.n71 For more information, visit: http://www.prisma-statement.org/

# Supplementary Material S2. Database and Search Strategies

PubMed

The retrieval of the PubMed database was conducted on November 14, 2021, and a total of 4 records were retrieved.

| Search | Query | Results | Time |
| --- | --- | --- | --- |
| #5 | Search: (("Diabetes Mellitus, Type 2"[Mesh]) OR (((((((((((((((((((((((((((((((Diabetes Mellitus, Noninsulin-Dependent[Title/Abstract]) OR (Diabetes Mellitus, Ketosis-Resistant[Title/Abstract])) OR (Diabetes Mellitus, Ketosis Resistant[Title/Abstract])) OR (Ketosis-Resistant Diabetes Mellitus[Title/Abstract])) OR (Diabetes Mellitus, Non Insulin Dependent[Title/Abstract])) OR (Diabetes Mellitus, Non-Insulin-Dependent[Title/Abstract])) OR (Non-Insulin-Dependent Diabetes Mellitus[Title/Abstract])) OR (Diabetes Mellitus, Stable[Title/Abstract])) OR (Stable Diabetes Mellitus[Title/Abstract])) OR (Diabetes Mellitus, Type II[Title/Abstract])) OR (NIDDM[Title/Abstract])) OR (Diabetes Mellitus, Noninsulin Dependent[Title/Abstract])) OR (Diabetes Mellitus, Maturity-Onset[Title/Abstract])) OR (Diabetes Mellitus, Maturity Onset[Title/Abstract])) OR (Maturity-Onset Diabetes Mellitus[Title/Abstract])) OR (Maturity Onset Diabetes Mellitus[Title/Abstract])) OR (MODY[Title/Abstract])) OR (Diabetes Mellitus, Slow-Onset[Title/Abstract])) OR (Diabetes Mellitus, Slow Onset[Title/Abstract])) OR (Slow-Onset Diabetes Mellitus[Title/Abstract])) OR (Type 2 Diabetes Mellitus[Title/Abstract])) OR (Noninsulin-Dependent Diabetes Mellitus[Title/Abstract])) OR (Noninsulin Dependent Diabetes Mellitus[Title/Abstract])) OR (Maturity-Onset Diabetes[Title/Abstract])) OR (Diabetes, Maturity-Onset[Title/Abstract])) OR (Maturity Onset Diabetes[Title/Abstract])) OR (Type 2 Diabetes[Title/Abstract])) OR (Diabetes, Type 2[Title/Abstract])) OR (Diabetes Mellitus, Adult-Onset[Title/Abstract])) OR (Adult-Onset Diabetes Mellitus[Title/Abstract])) OR (Diabetes Mellitus, Adult Onset[Title/Abstract]))) AND (((((((((Dachaihu[Title/Abstract]) OR (Dachaihu Decoction[Title/Abstract])) OR (Dachaihu Tang[Title/Abstract])) OR (Da Chaihu[Title/Abstract])) OR (Da Chaihu Tang[Title/Abstract])) OR (Da Chaihu Decoction[Title/Abstract])) OR (Major Bupleurum Decoction[Title/Abstract])) OR (Major Bupleurum Tang[Title/Abstract])) OR (daisaikoto[Title/Abstract])) | 4 | 07:30:21 |
| #4 | Search: ((((((((Dachaihu[Title/Abstract]) OR (Dachaihu Decoction[Title/Abstract])) OR (Dachaihu Tang[Title/Abstract])) OR (Da Chaihu[Title/Abstract])) OR (Da Chaihu Tang[Title/Abstract])) OR (Da Chaihu Decoction[Title/Abstract])) OR (Major Bupleurum Decoction[Title/Abstract])) OR (Major Bupleurum Tang[Title/Abstract])) OR (daisaikoto[Title/Abstract]) | 76 | 07:29:41 |
| #3 | Search: ("Diabetes Mellitus, Type 2"[Mesh]) OR (((((((((((((((((((((((((((((((Diabetes Mellitus, Noninsulin-Dependent[Title/Abstract]) OR (Diabetes Mellitus, Ketosis-Resistant[Title/Abstract])) OR (Diabetes Mellitus, Ketosis Resistant[Title/Abstract])) OR (Ketosis-Resistant Diabetes Mellitus[Title/Abstract])) OR (Diabetes Mellitus, Non Insulin Dependent[Title/Abstract])) OR (Diabetes Mellitus, Non-Insulin-Dependent[Title/Abstract])) OR (Non-Insulin-Dependent Diabetes Mellitus[Title/Abstract])) OR (Diabetes Mellitus, Stable[Title/Abstract])) OR (Stable Diabetes Mellitus[Title/Abstract])) OR (Diabetes Mellitus, Type II[Title/Abstract])) OR (NIDDM[Title/Abstract])) OR (Diabetes Mellitus, Noninsulin Dependent[Title/Abstract])) OR (Diabetes Mellitus, Maturity-Onset[Title/Abstract])) OR (Diabetes Mellitus, Maturity Onset[Title/Abstract])) OR (Maturity-Onset Diabetes Mellitus[Title/Abstract])) OR (Maturity Onset Diabetes Mellitus[Title/Abstract])) OR (MODY[Title/Abstract])) OR (Diabetes Mellitus, Slow-Onset[Title/Abstract])) OR (Diabetes Mellitus, Slow Onset[Title/Abstract])) OR (Slow-Onset Diabetes Mellitus[Title/Abstract])) OR (Type 2 Diabetes Mellitus[Title/Abstract])) OR (Noninsulin-Dependent Diabetes Mellitus[Title/Abstract])) OR (Noninsulin Dependent Diabetes Mellitus[Title/Abstract])) OR (Maturity-Onset Diabetes[Title/Abstract])) OR (Diabetes, Maturity-Onset[Title/Abstract])) OR (Maturity Onset Diabetes[Title/Abstract])) OR (Type 2 Diabetes[Title/Abstract])) OR (Diabetes, Type 2[Title/Abstract])) OR (Diabetes Mellitus, Adult-Onset[Title/Abstract])) OR (Adult-Onset Diabetes Mellitus[Title/Abstract])) OR (Diabetes Mellitus, Adult Onset[Title/Abstract])) | 206,151 | 04:52:31 |
| #2 | Search: ((((((((((((((((((((((((((((((Diabetes Mellitus, Noninsulin-Dependent[Title/Abstract]) OR (Diabetes Mellitus, Ketosis-Resistant[Title/Abstract])) OR (Diabetes Mellitus, Ketosis Resistant[Title/Abstract])) OR (Ketosis-Resistant Diabetes Mellitus[Title/Abstract])) OR (Diabetes Mellitus, Non Insulin Dependent[Title/Abstract])) OR (Diabetes Mellitus, Non-Insulin-Dependent[Title/Abstract])) OR (Non-Insulin-Dependent Diabetes Mellitus[Title/Abstract])) OR (Diabetes Mellitus, Stable[Title/Abstract])) OR (Stable Diabetes Mellitus[Title/Abstract])) OR (Diabetes Mellitus, Type II[Title/Abstract])) OR (NIDDM[Title/Abstract])) OR (Diabetes Mellitus, Noninsulin Dependent[Title/Abstract])) OR (Diabetes Mellitus, Maturity-Onset[Title/Abstract])) OR (Diabetes Mellitus, Maturity Onset[Title/Abstract])) OR (Maturity-Onset Diabetes Mellitus[Title/Abstract])) OR (Maturity Onset Diabetes Mellitus[Title/Abstract])) OR (MODY[Title/Abstract])) OR (Diabetes Mellitus, Slow-Onset[Title/Abstract])) OR (Diabetes Mellitus, Slow Onset[Title/Abstract])) OR (Slow-Onset Diabetes Mellitus[Title/Abstract])) OR (Type 2 Diabetes Mellitus[Title/Abstract])) OR (Noninsulin-Dependent Diabetes Mellitus[Title/Abstract])) OR (Noninsulin Dependent Diabetes Mellitus[Title/Abstract])) OR (Maturity-Onset Diabetes[Title/Abstract])) OR (Diabetes, Maturity-Onset[Title/Abstract])) OR (Maturity Onset Diabetes[Title/Abstract])) OR (Type 2 Diabetes[Title/Abstract])) OR (Diabetes, Type 2[Title/Abstract])) OR (Diabetes Mellitus, Adult-Onset[Title/Abstract])) OR (Adult-Onset Diabetes Mellitus[Title/Abstract])) OR (Diabetes Mellitus, Adult Onset[Title/Abstract]) | 157,519 | 04:51:47 |
| #1 | Search: "Diabetes Mellitus, Type 2"[Mesh] Sort by: Most Recent | 148,845 | 04:46:02 |

Embase

The retrieval of the Embase database was conducted on November 14, 2021, and a total of 7 records were retrieved.

| History |  | Results |
| --- | --- | --- |
| #6 | #4 AND #5 | 7 |
| #5 | dachaihu:ab,ti OR 'dachaihu decoction':ab,ti OR 'dachaihu tang':ab,ti OR 'da chaihu':ab,ti OR 'da chaihu tang':ab,ti OR 'da chaihu decoction':ab,ti OR 'major bupleurum decoction':ab,ti OR 'major bupleurum tang':ab,ti | 34 |
| #4 | 'daisaikoto'/exp | 120 |
| #3 | #1 AND #2 | 192,731 |
| #2 | 'diabetes mellitus, type 2':ab,ti OR 'diabetes mellitus, noninsulin-dependent':ab,ti OR 'diabetes mellitus, ketosis-resistant':ab,ti OR 'diabetes mellitus, ketosis resistant':ab,ti OR 'ketosis-resistant diabetes mellitus':ab,ti OR 'diabetes mellitus, non insulin dependent':ab,ti OR 'diabetes mellitus, non-insulin-dependent':ab,ti OR 'non-insulin-dependent diabetes mellitus':ab,ti OR 'diabetes mellitus, stable':ab,ti OR 'stable diabetes mellitus':ab,ti OR 'diabetes mellitus, type ii':ab,ti OR niddm:ab,ti OR 'diabetes mellitus, noninsulin dependent':ab,ti OR 'diabetes mellitus, maturity-onset':ab,ti OR 'diabetes mellitus, maturity onset':ab,ti OR 'maturity-onset diabetes mellitus':ab,ti OR 'maturity onset diabetes mellitus':ab,ti OR mody:ab,ti OR 'diabetes mellitus, slow-onset':ab,ti OR 'diabetes mellitus, slow onset':ab,ti OR 'slow-onset diabetes mellitus':ab,ti OR 'type 2 diabetes mellitus':ab,ti OR 'noninsulin-dependent diabetes mellitus':ab,ti OR 'noninsulin dependent diabetes mellitus':ab,ti OR 'maturity-onset diabetes':ab,ti OR 'diabetes, maturity-onset':ab,ti OR 'maturity onset diabetes':ab,ti OR 'type 2 diabetes':ab,ti OR 'diabetes, type 2':ab,ti OR 'diabetes mellitus, adult-onset':ab,ti OR 'adult-onset diabetes mellitus':ab,ti OR 'diabetes mellitus, adult onset':ab,ti | 235,036 |
| #1 | 'non insulin dependent diabetes mellitus'/exp | 290,021 |


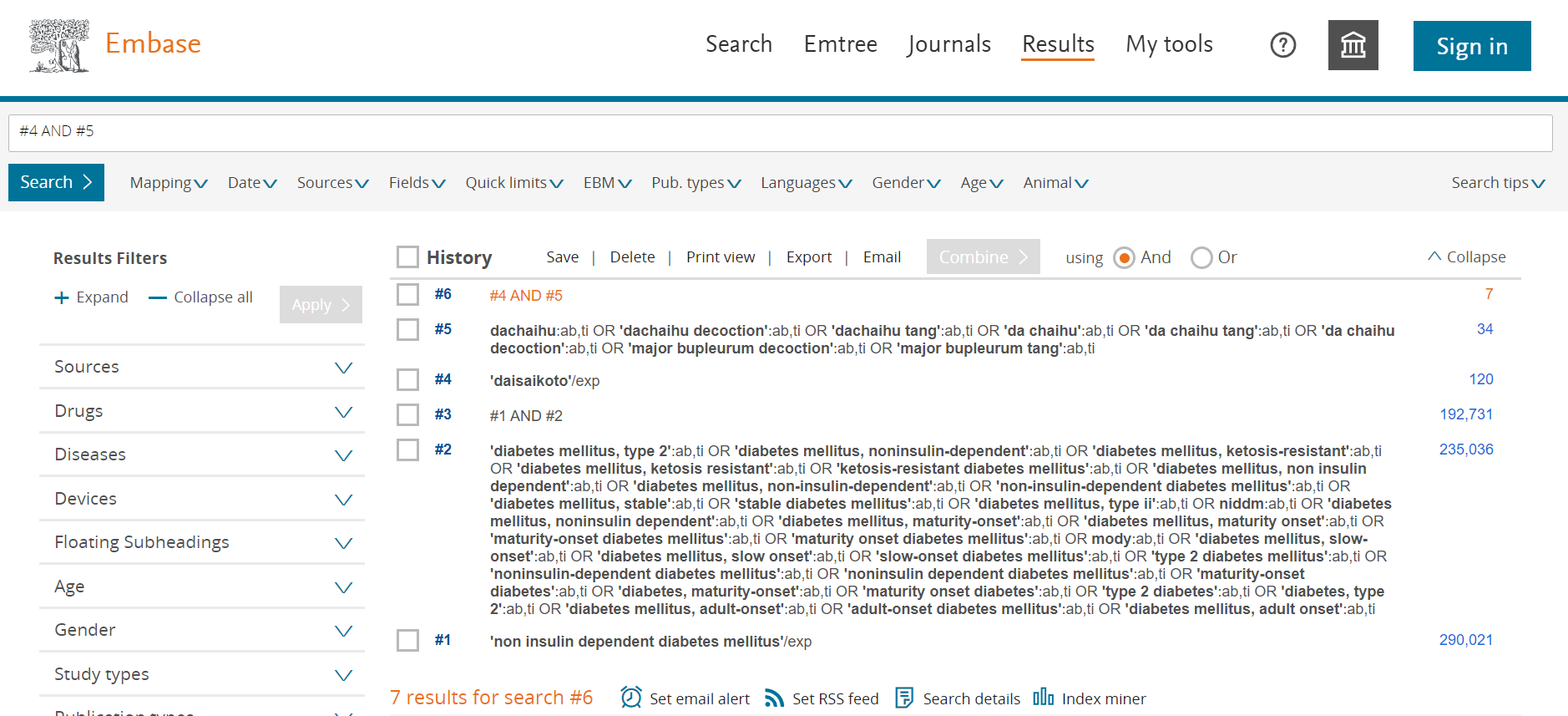


Cochrane

The retrieval of the Cochrane Library was conducted on November 14, 2021, and no records were retrieved.


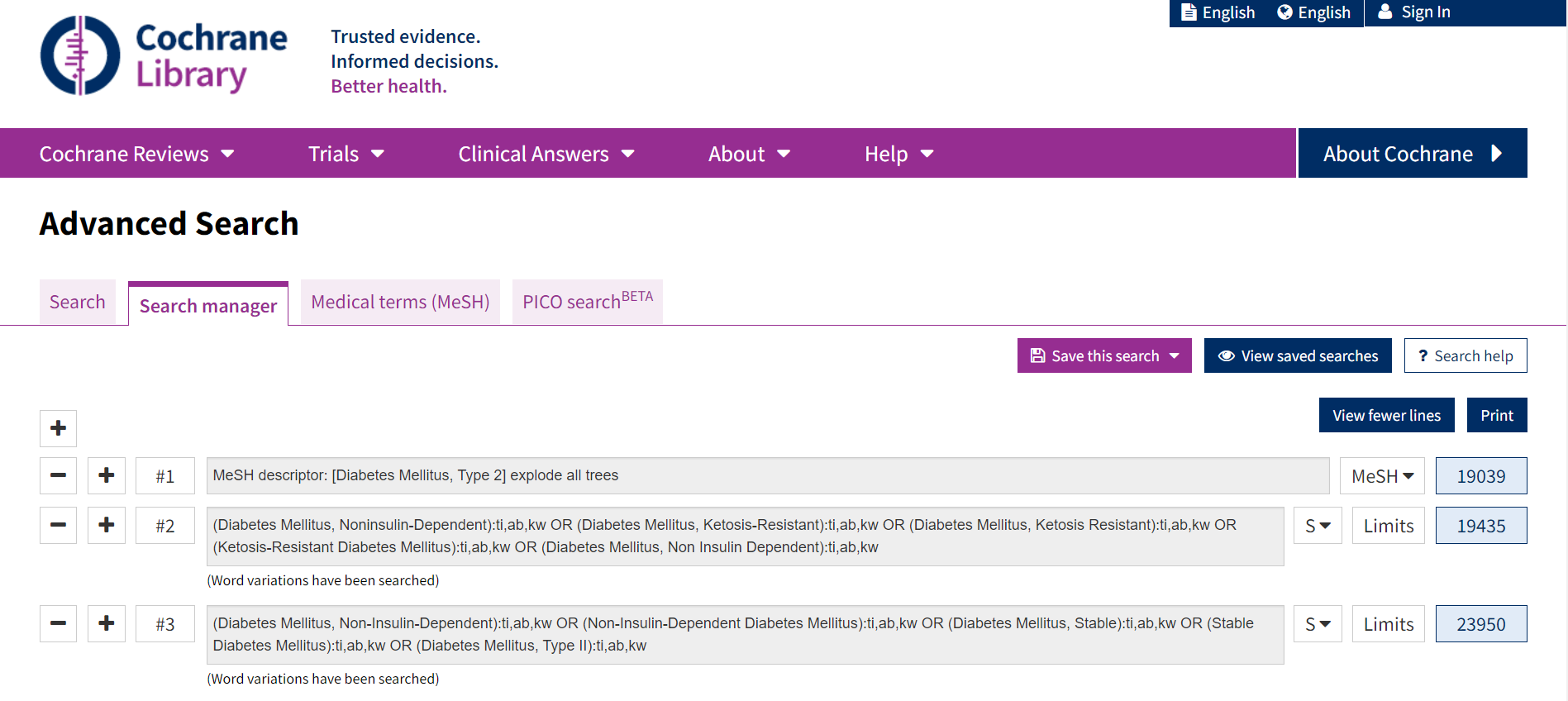


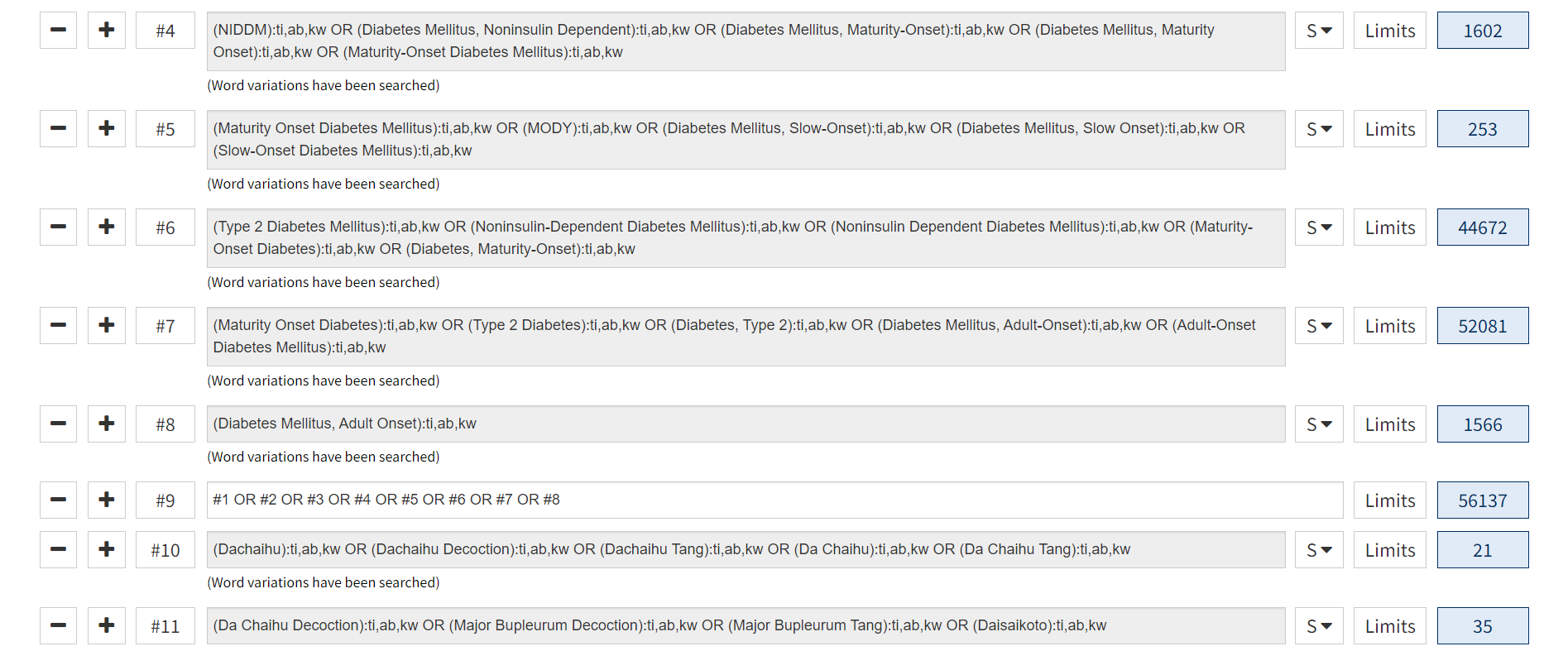


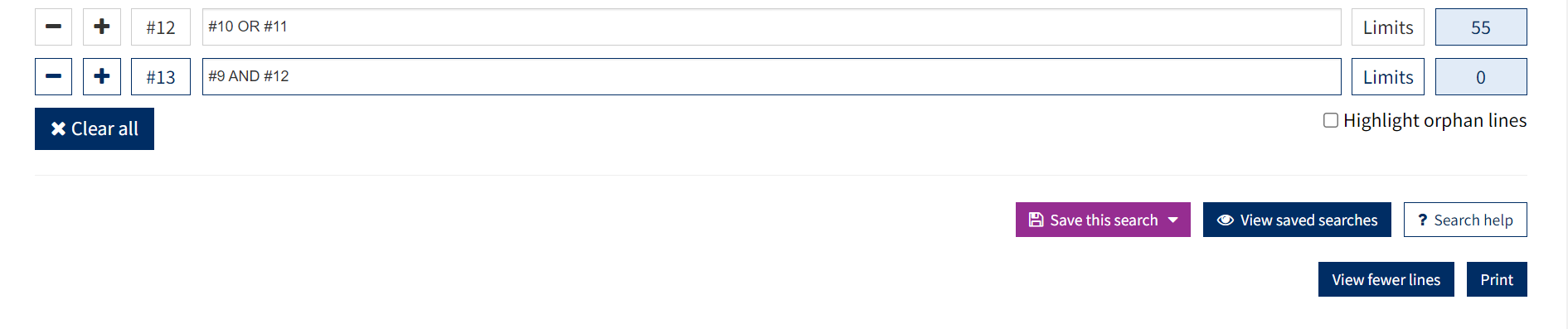


CNKI

The retrieval of the CNKI database was conducted on November 14, 2021, and a total of 215 records were retrieved.


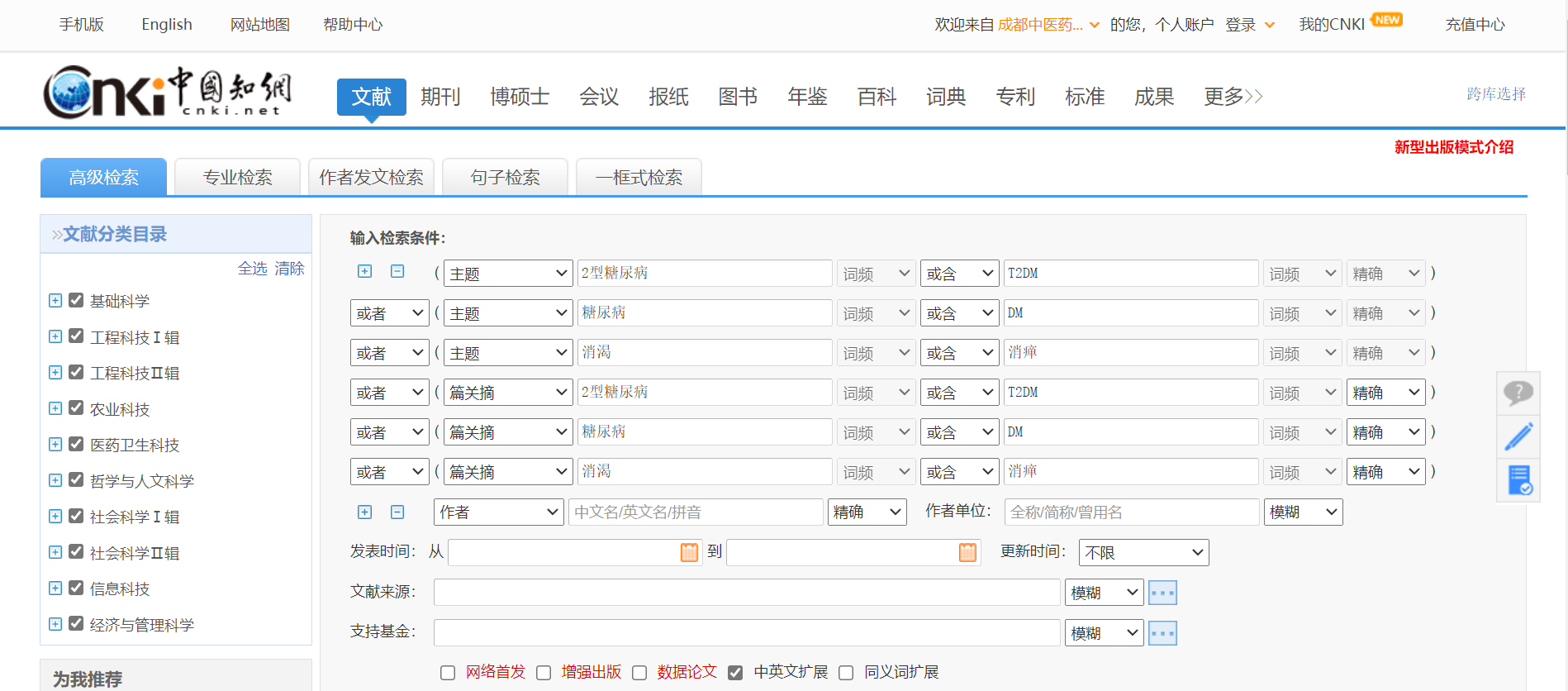


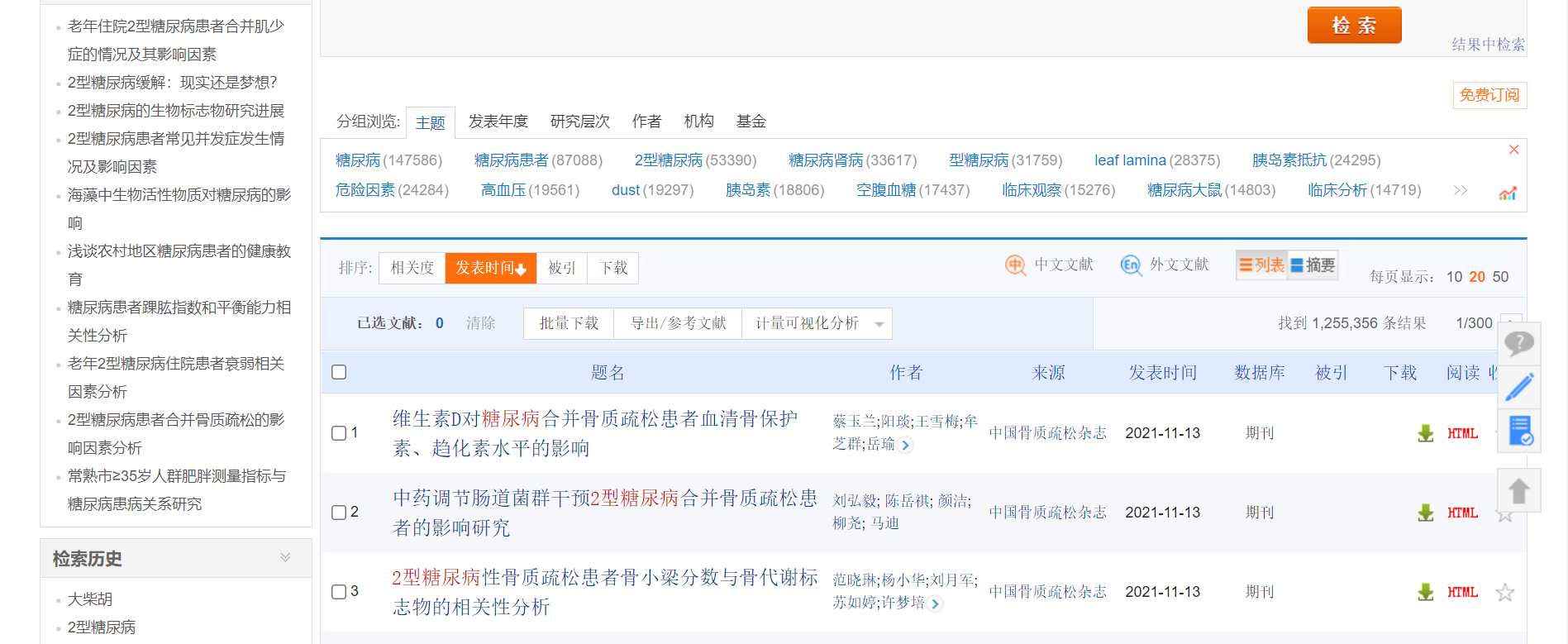


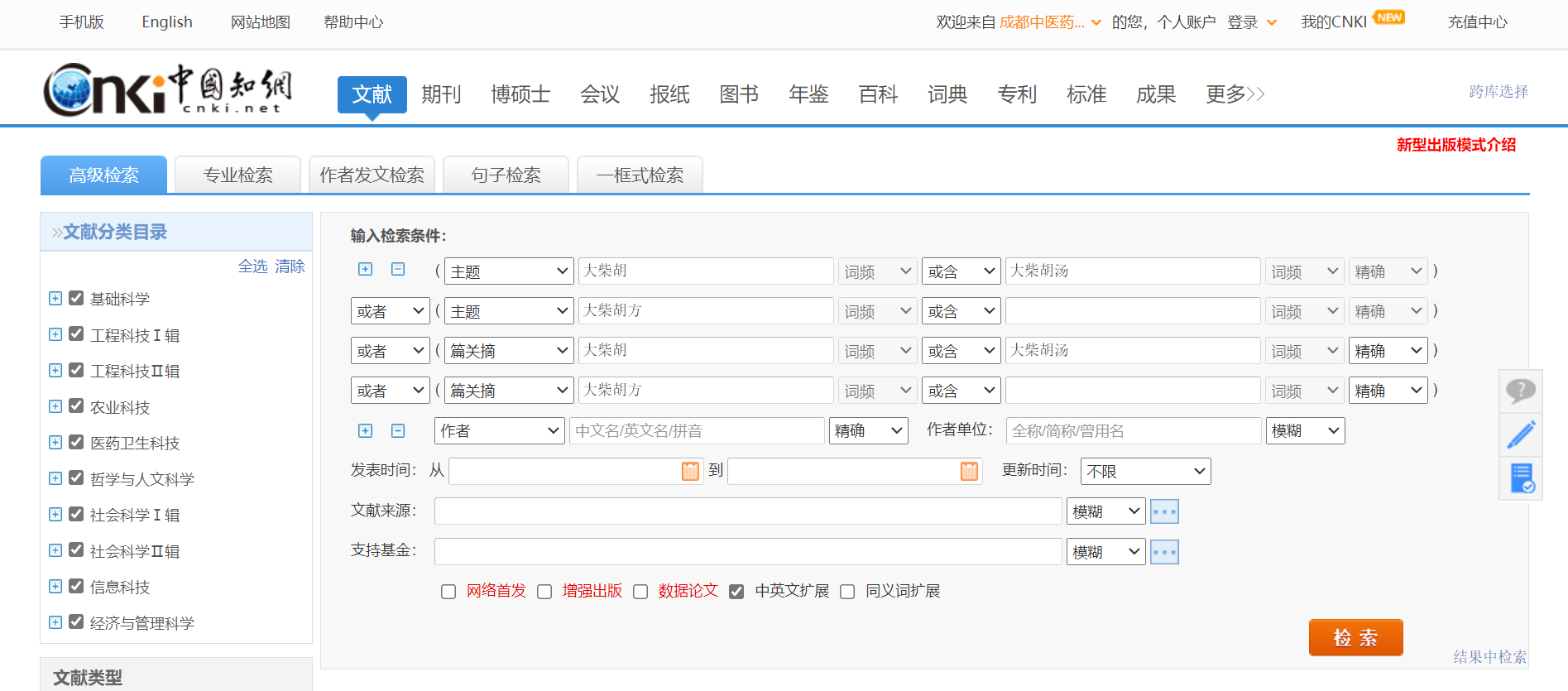


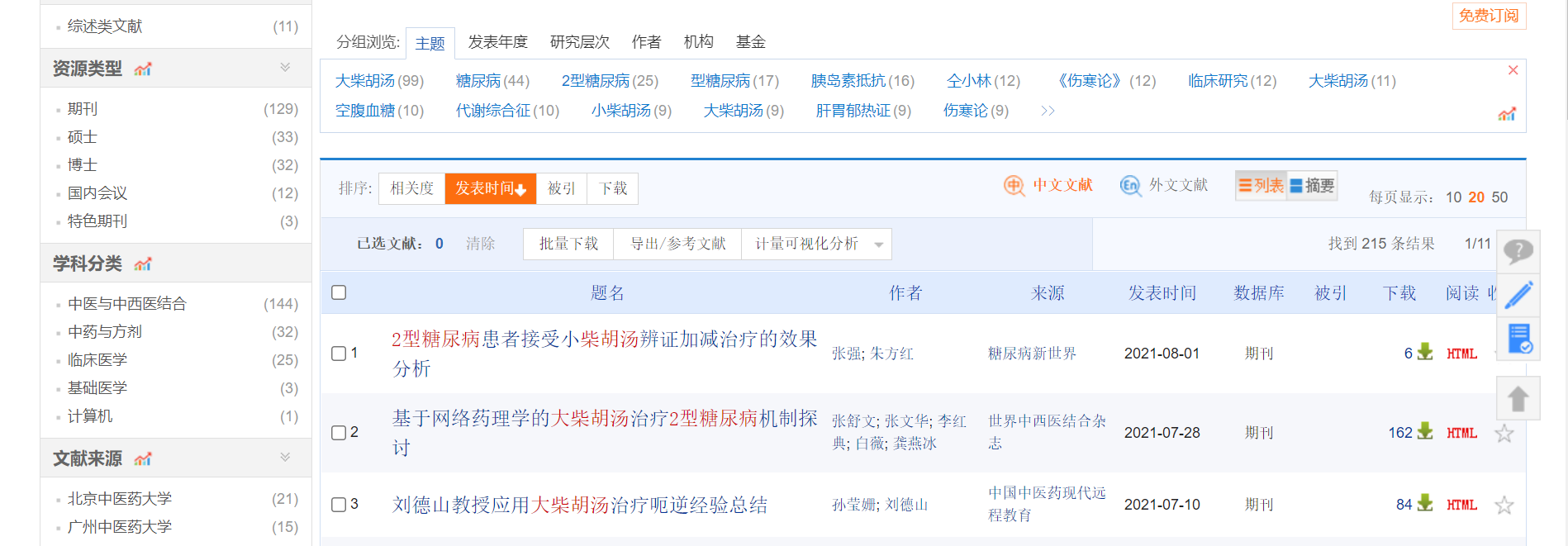


VIP

The retrieval of the VIP database was conducted on November 14, 2021, and a total of 77 records were retrieved.

((((((M=2型糖尿病 OR M=T2DM) OR M=糖尿病) OR M=DM) OR M=消渴) OR M=消瘅) OR (((((R=2型糖尿病 OR R=T2DM) OR R=糖尿病) OR R=DM) OR R=消渴) OR R=消瘅)) AND ((((((M=大柴胡 OR M=大柴胡汤) OR M=大柴胡方) OR R=大柴胡) OR R=大柴胡汤) OR R=大柴胡方))


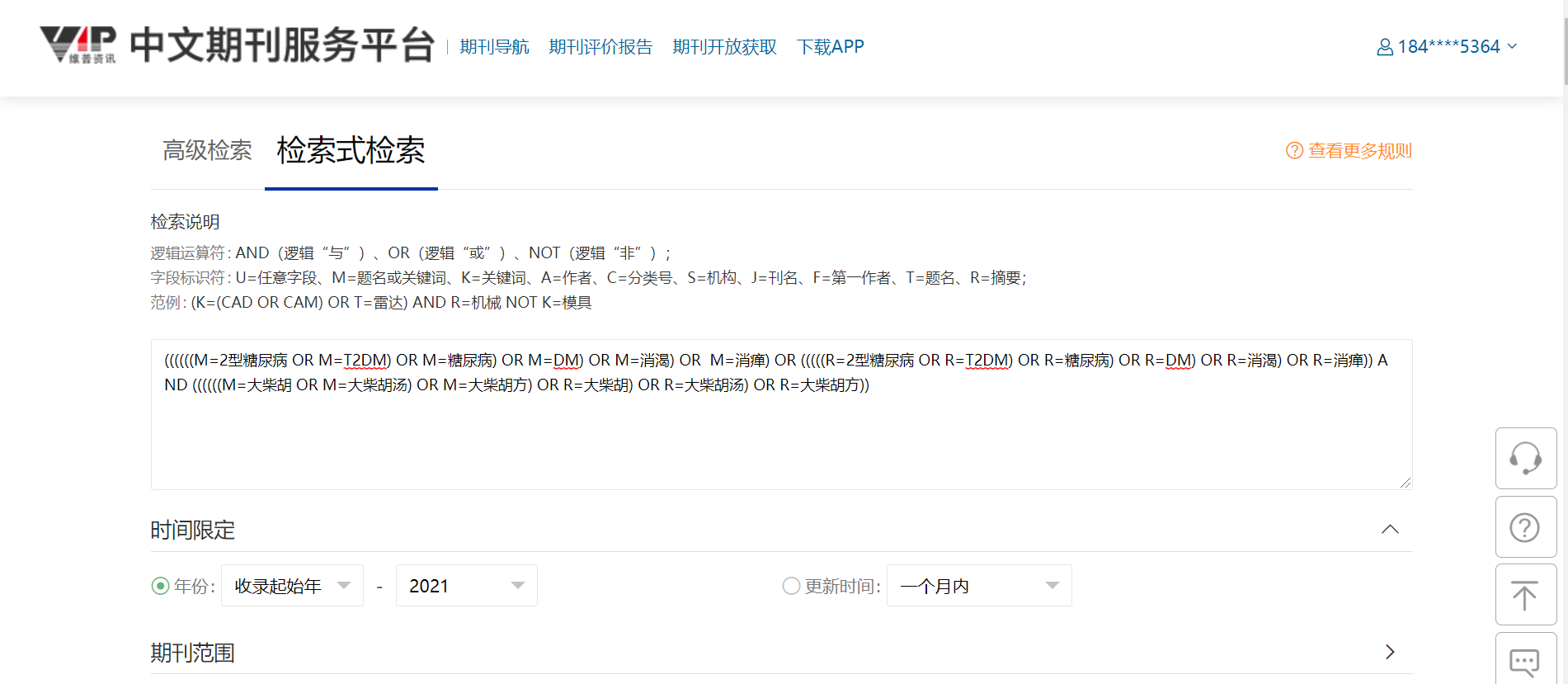


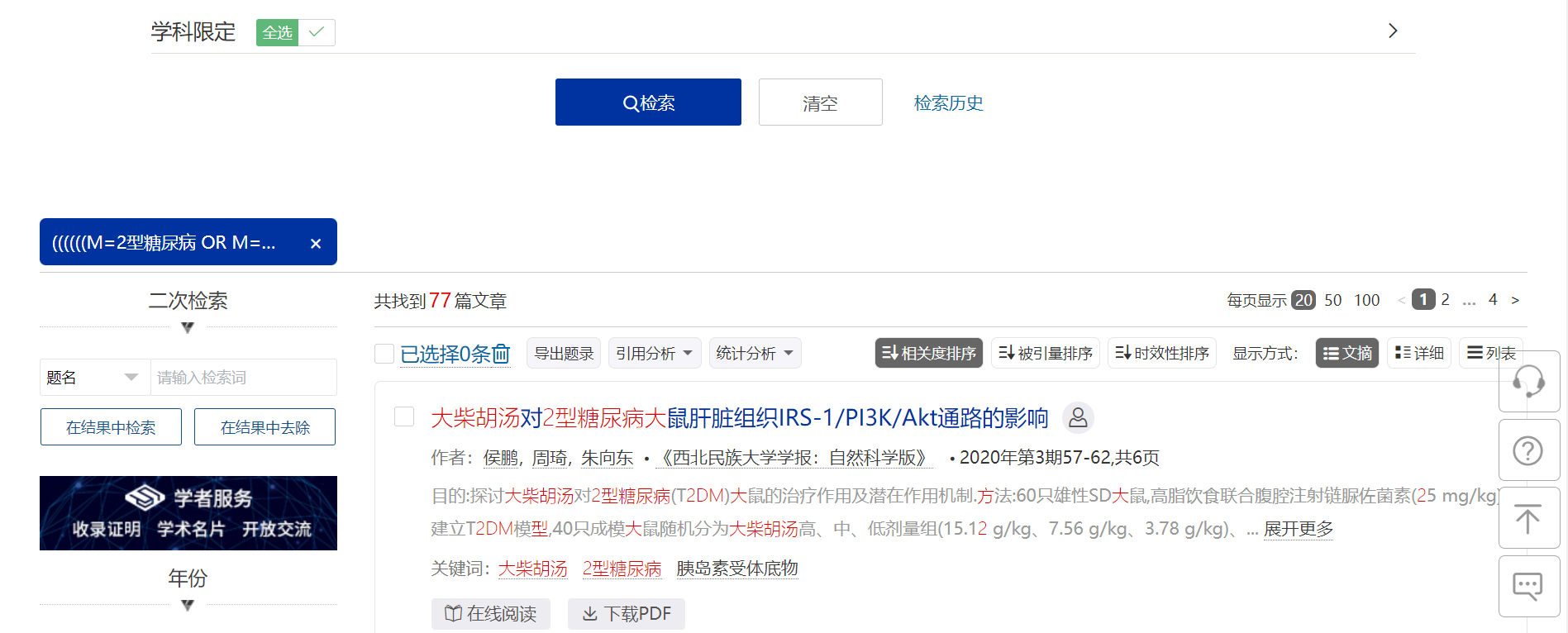


Wan Fang

The retrieval of the Wan Fang database was conducted on November 14, 2021, and a total of 324 records were retrieved.

(主题:(2型糖尿病) or 主题:(T2DM) or 主题:(糖尿病) or 主题:(DM) or 主题:(消渴) or 主题:(消瘅) or 题名或关键词:(2型糖尿病) or 题名或关键词:(T2DM) or 题名或关键词:(糖尿病) or 题名或关键词:(DM) or 题名或关键词:(消渴) or 题名或关键词:(消瘅)) AND (主题:(大柴胡) or 主题:(大柴胡汤) or 主题:(大柴胡方) or 题名或关键词:(大柴胡) or 题名或关键词:(大柴胡汤) or 题名或关键词:(大柴胡方))


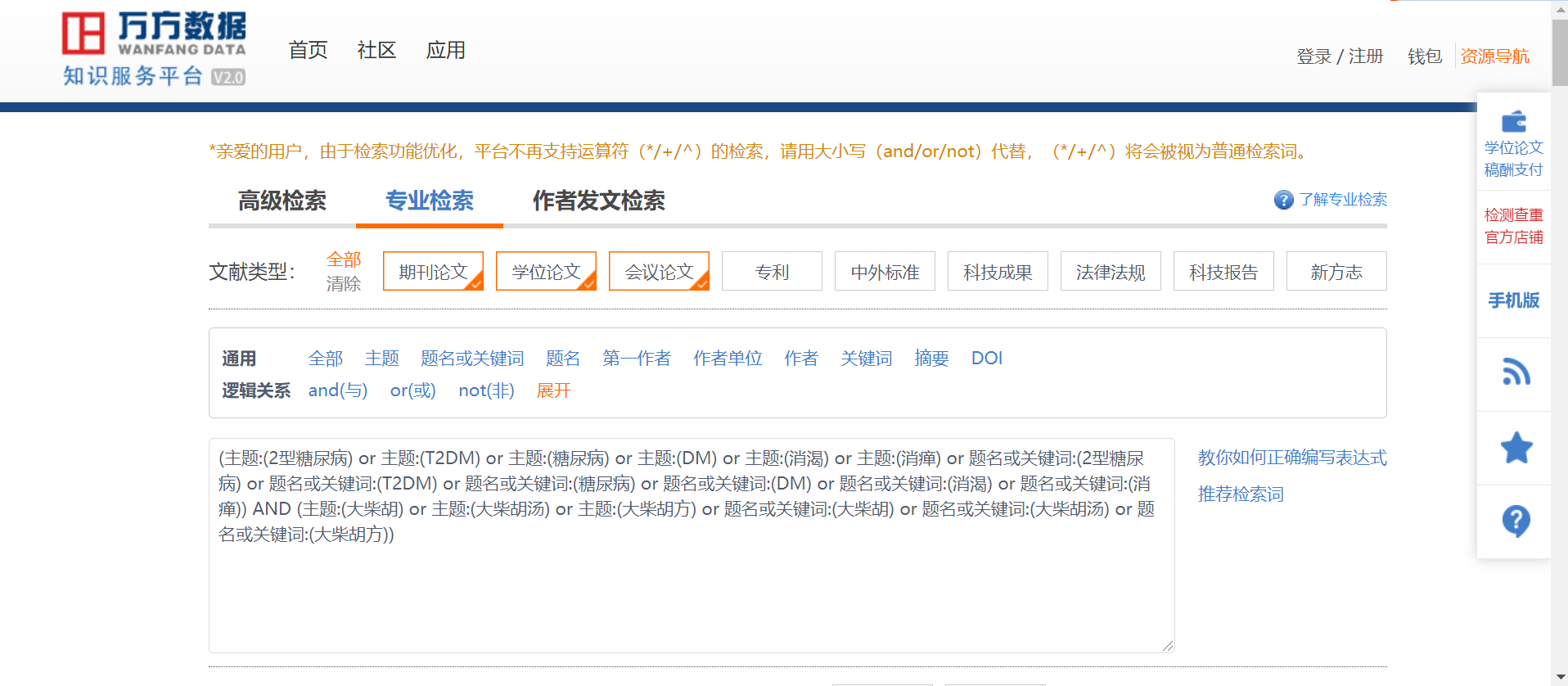


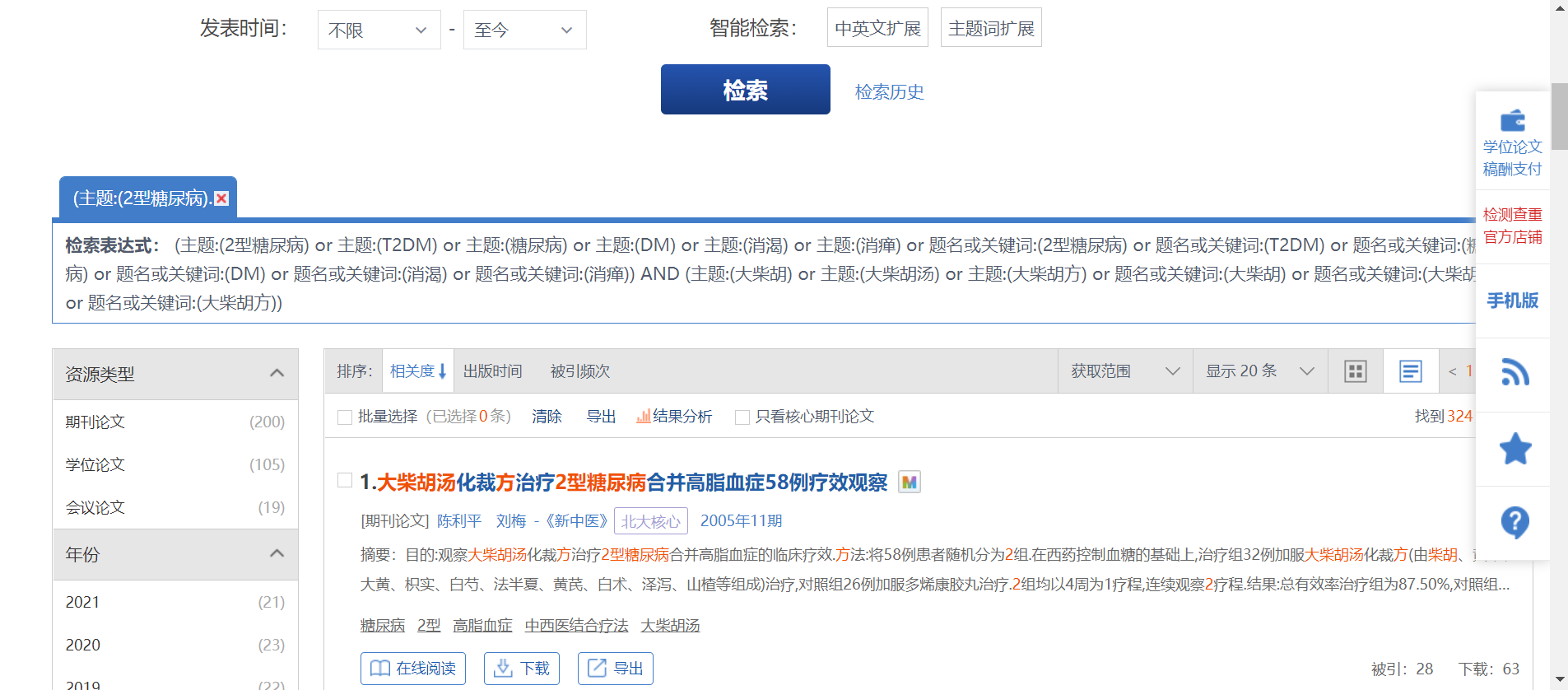


ClinicalTrials.gov

The retrieval of the ClinicalTrials.gov database was conducted on November 14, 2021, and no records were retrieved.


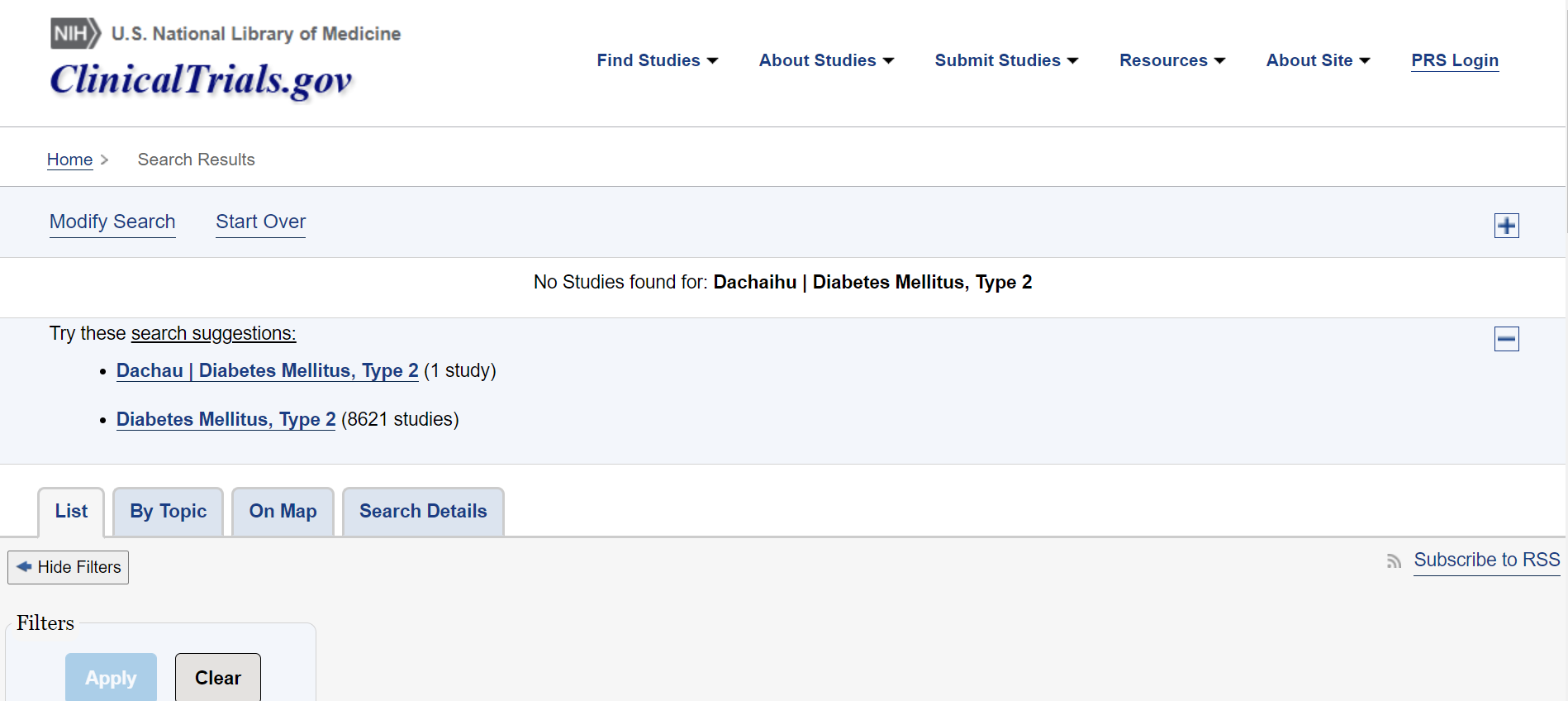


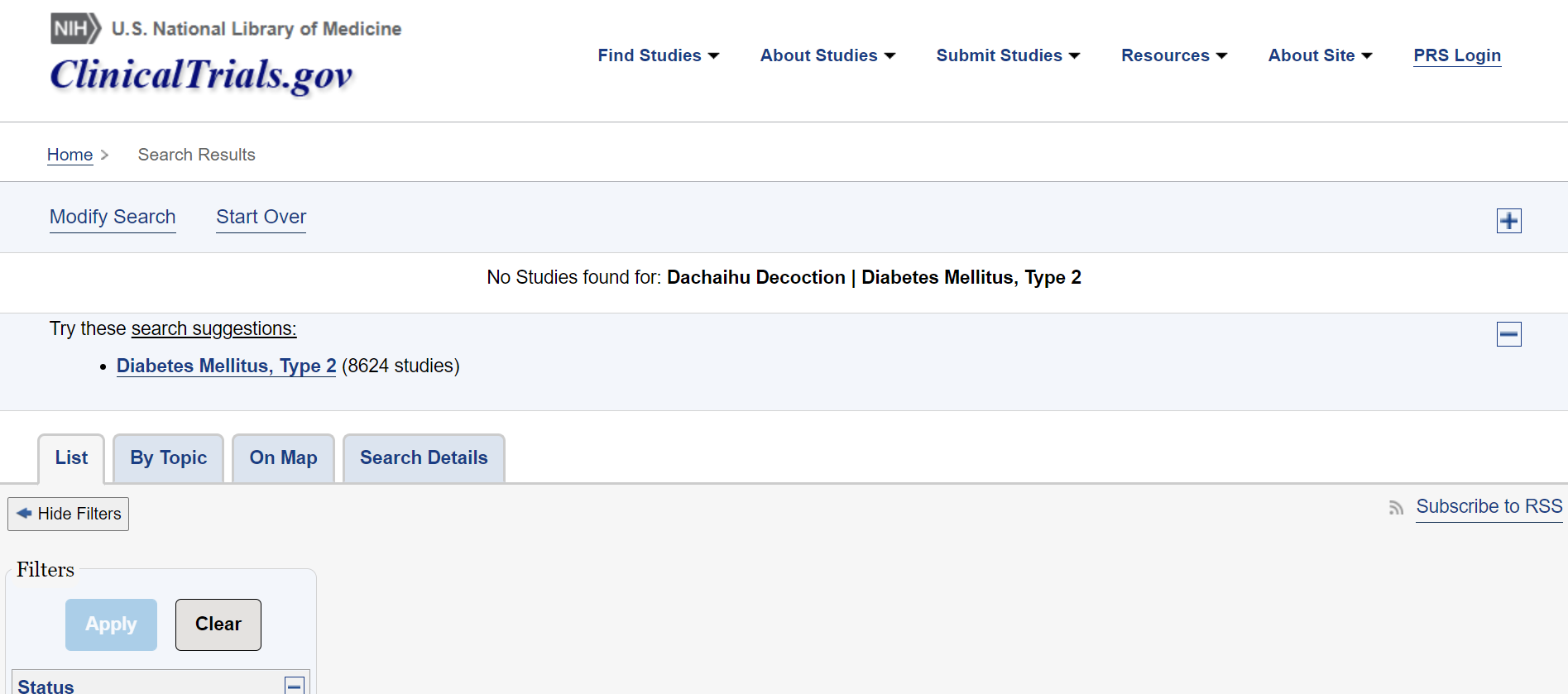


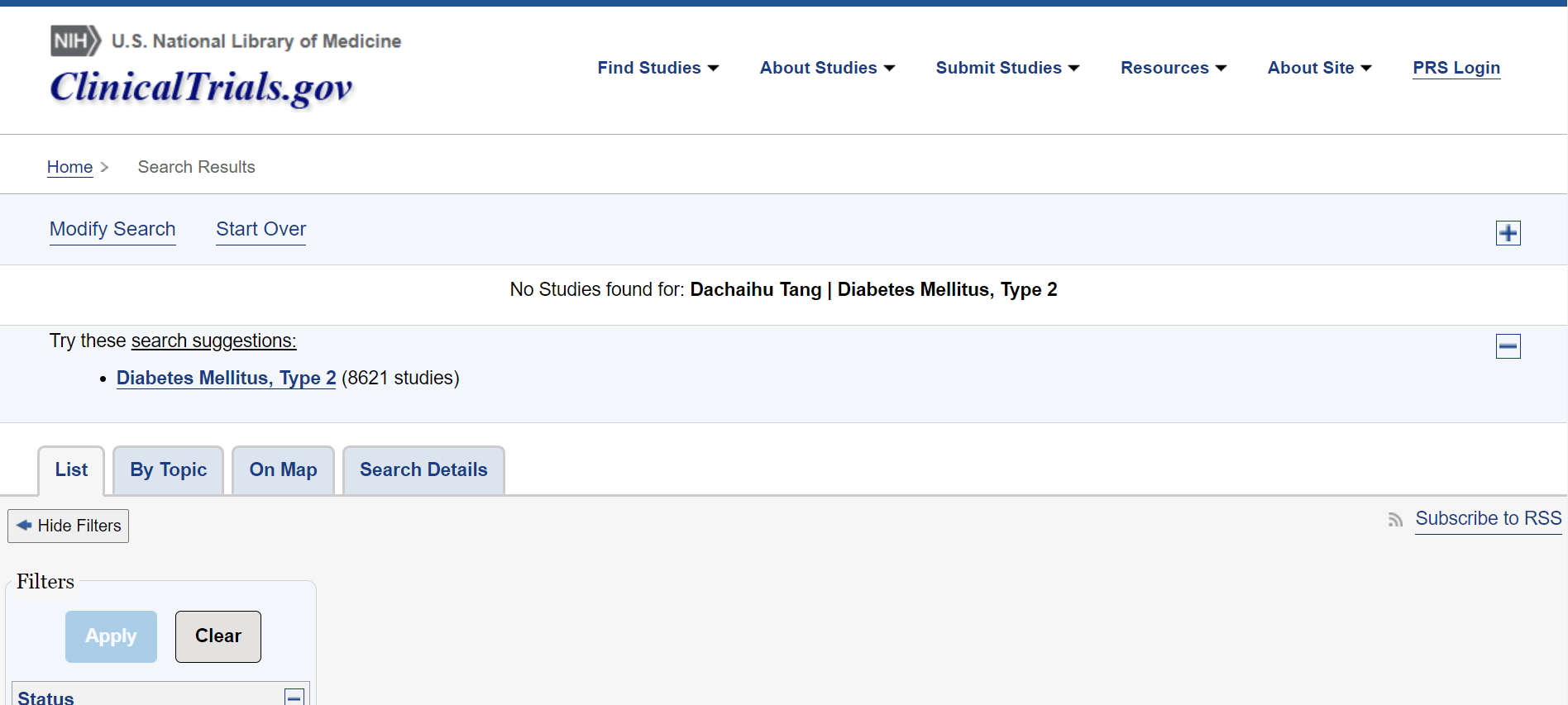


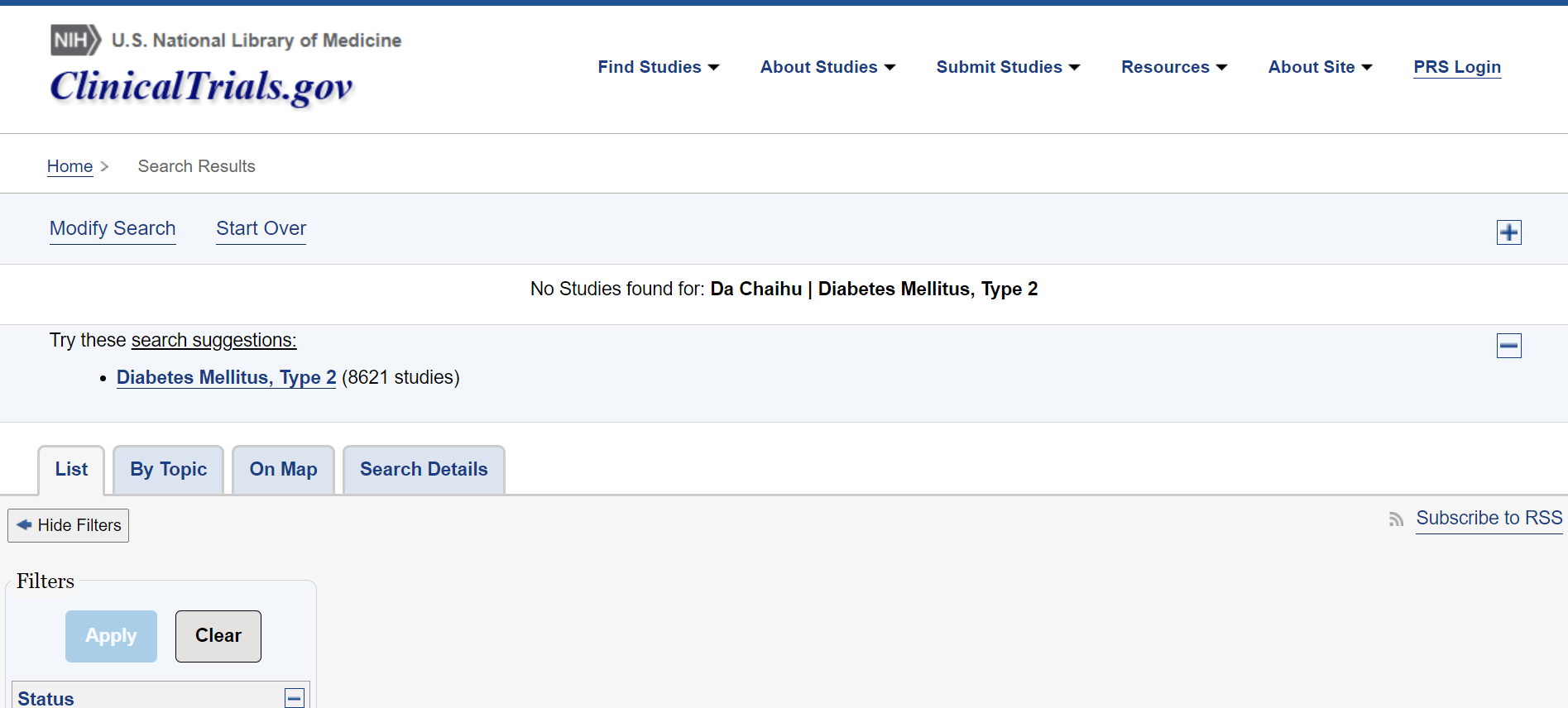


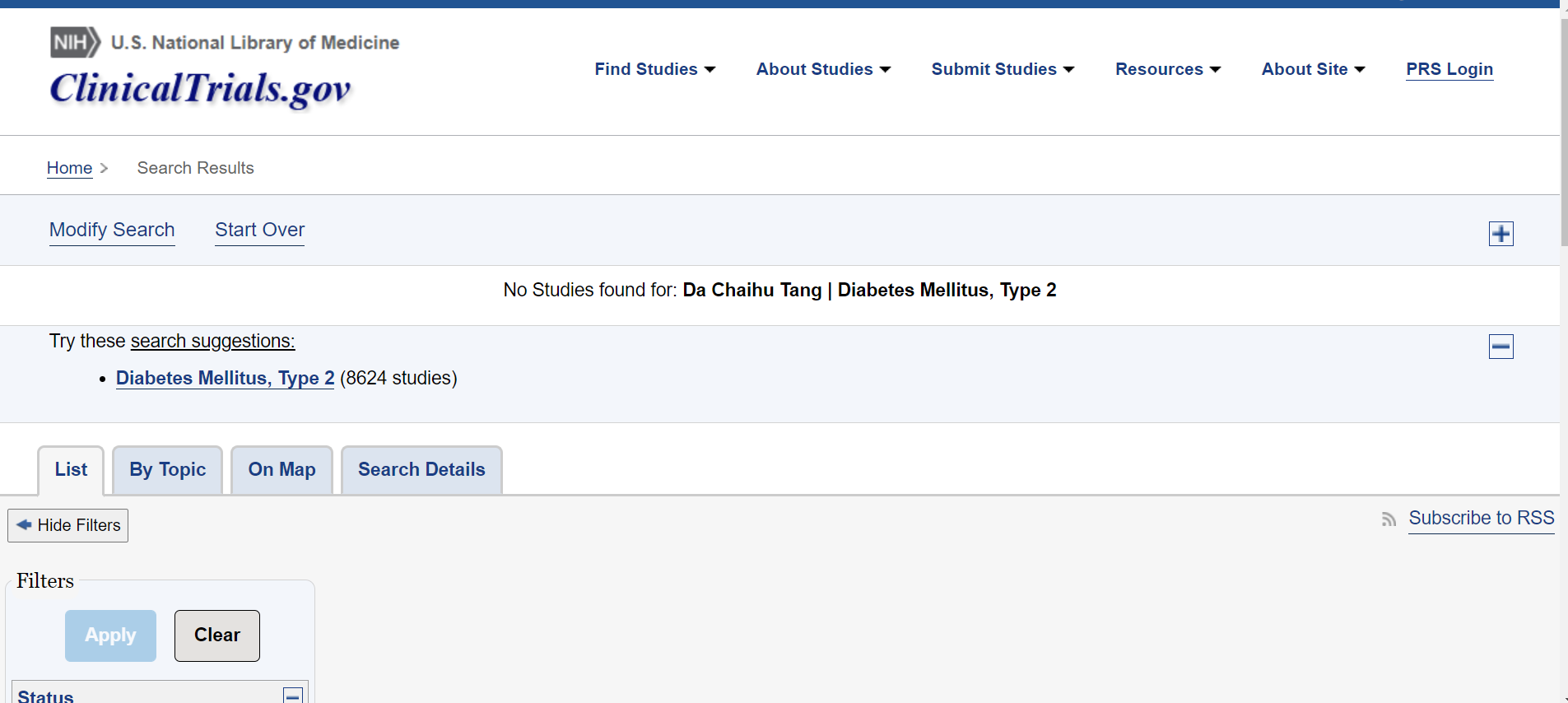


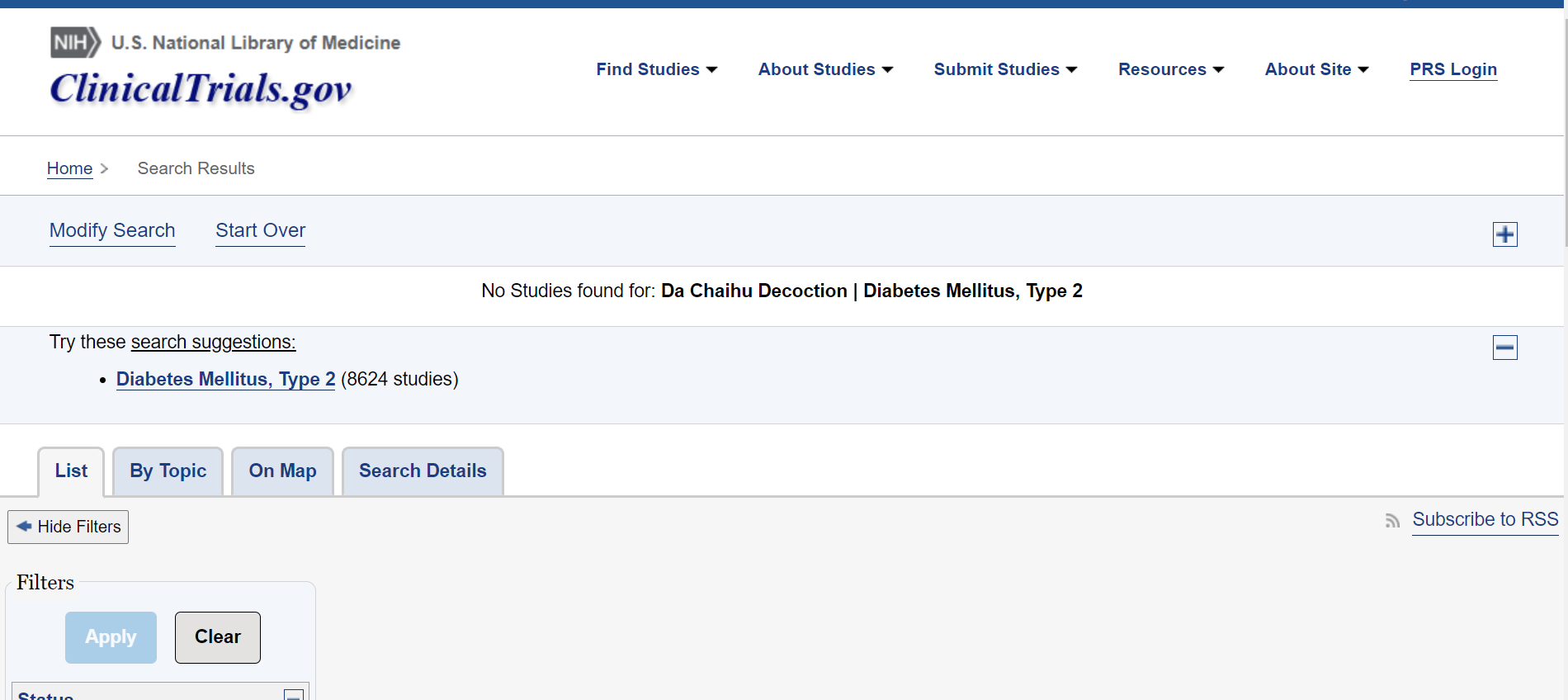


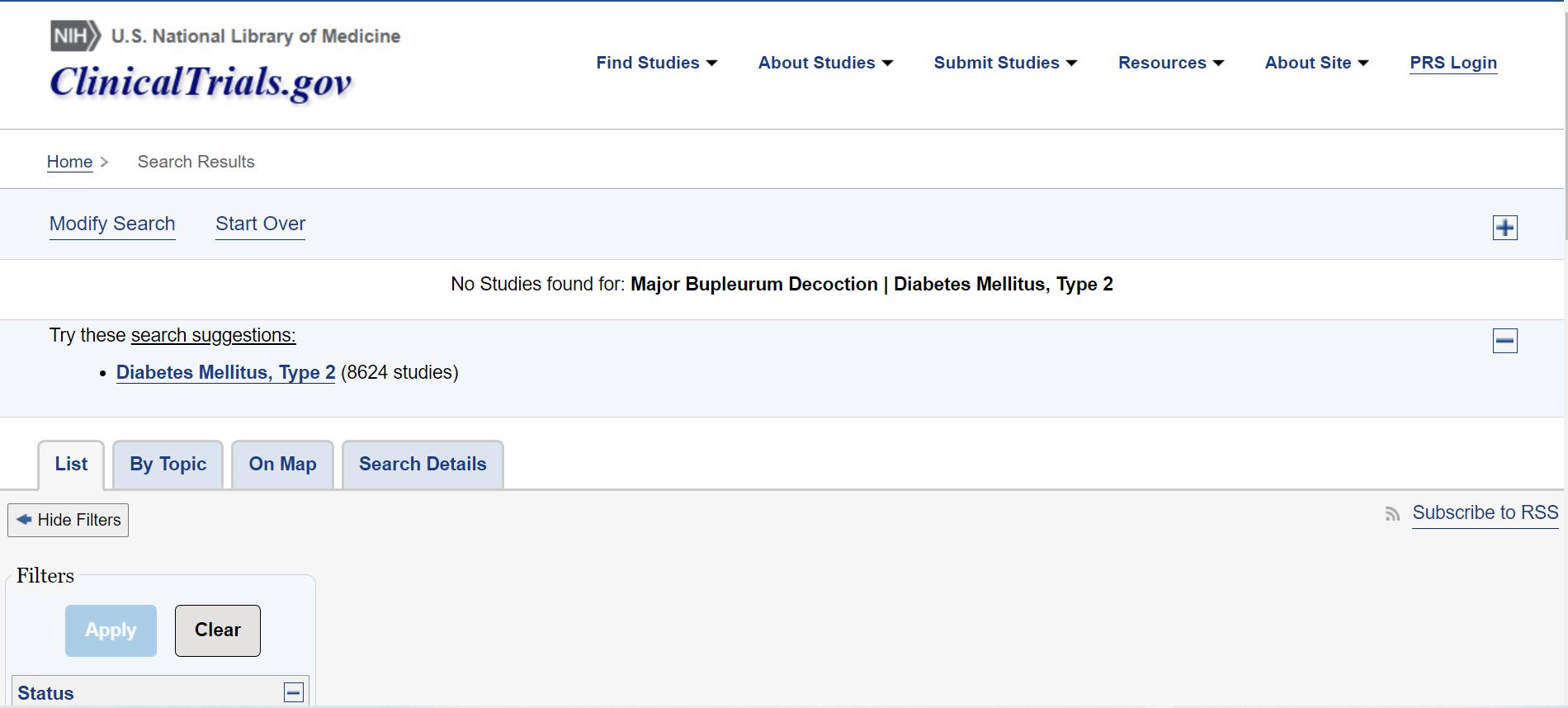


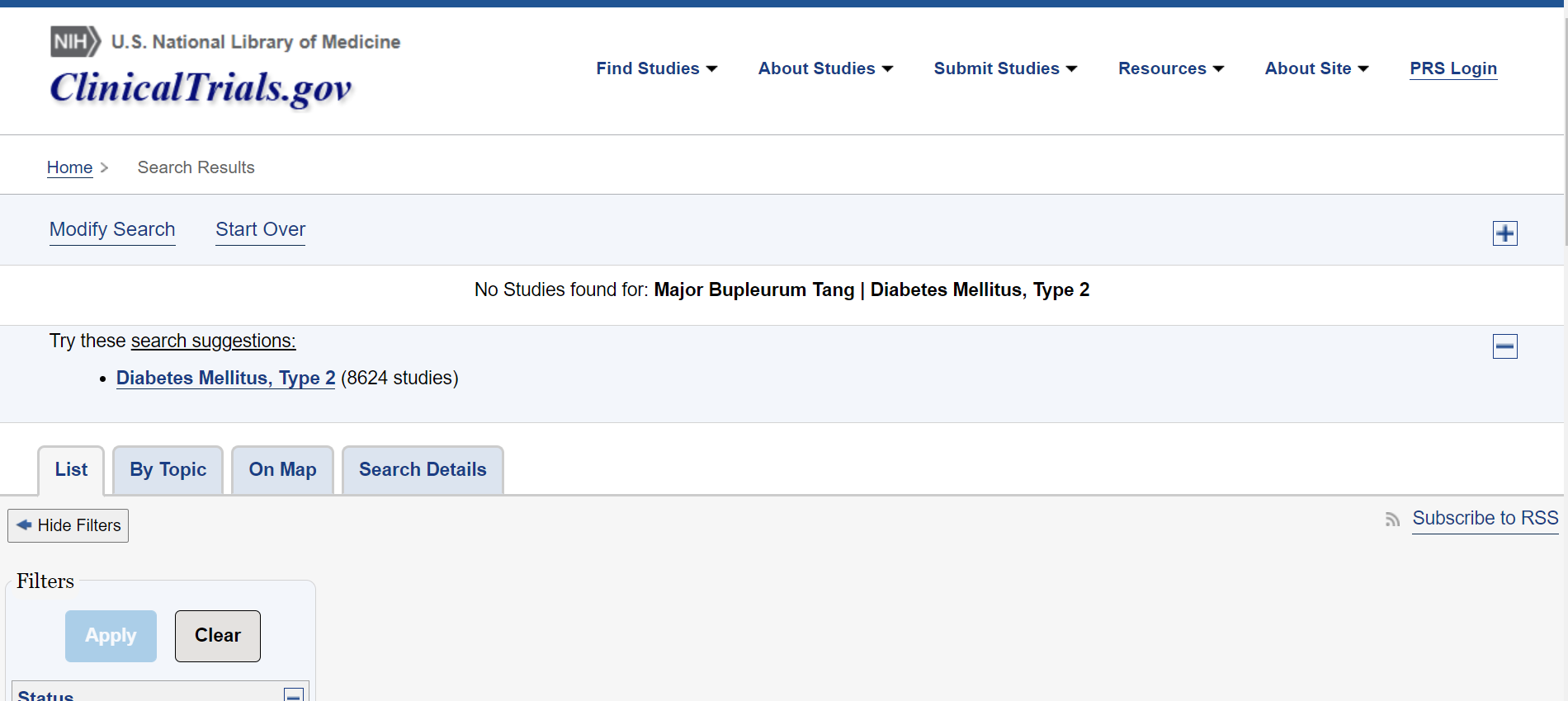


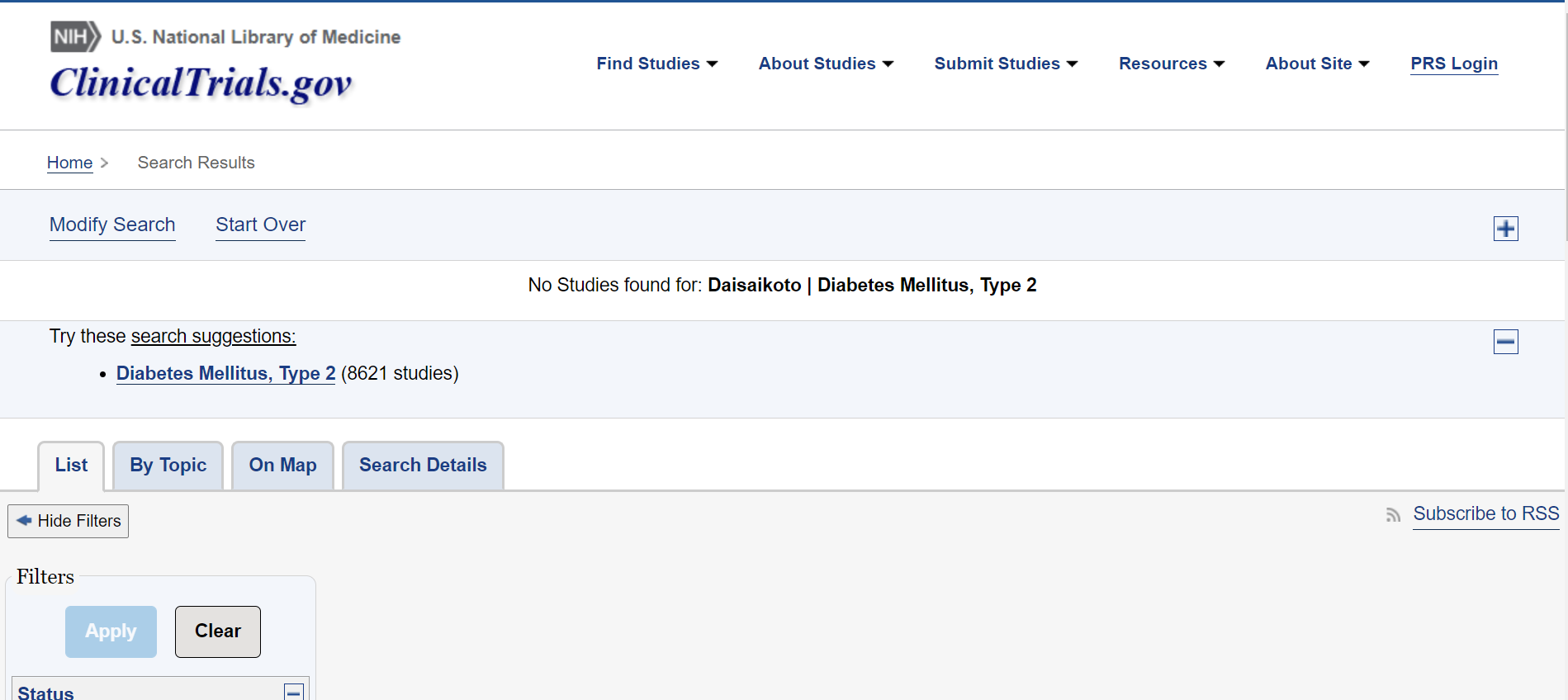


Chinese Clinical Trial Registry

The retrieval of the Chinese Clinical Trial Registry was conducted on November 14, 2021, and no records were retrieved.


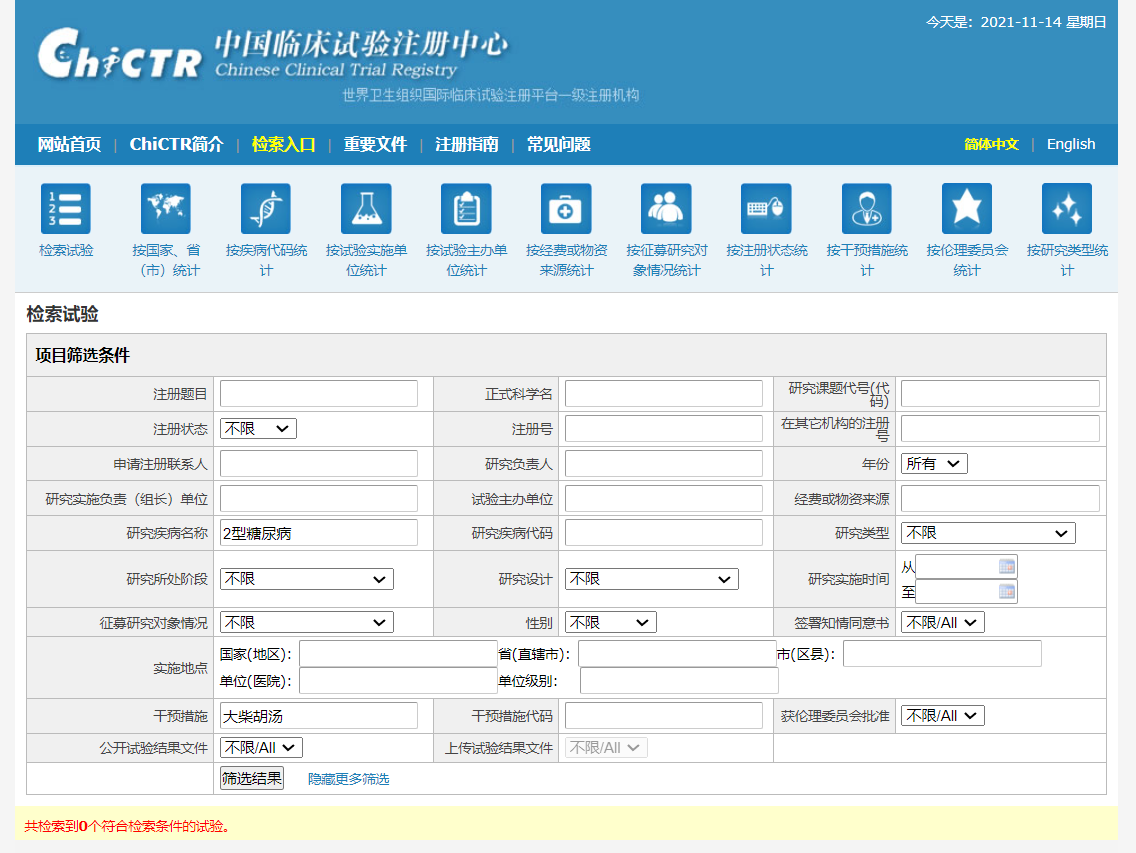


# Supplementary Material S3. The procedure for preliminary screening of the literature

**
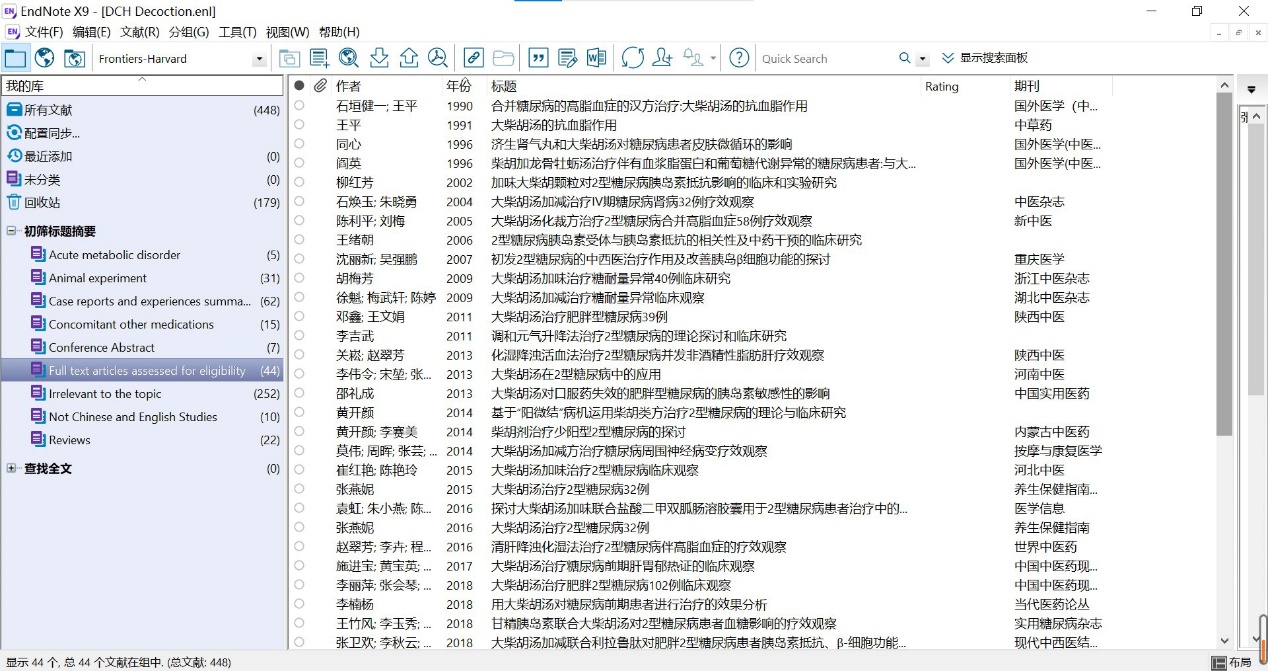
**

# Supplementary Material S4. Literature excluded after reading the full text and reasons

**1) The study design was not a randomized controlled trial:**

[1] Li, W.L., Song, K., and Zhang, X.J. (2013). Application of Dachaihu Decoction in Type 2 Diabetes. Henan Tradit Chin Med 33(3), 336-337. doi: 10.16367/j.issn.1003-5028.2013.03.013. (This is a review.)

[2] Huang, K.Y. (2014). The Study of "Slight Accumulation of Yang" and the Application of Chaihu Decoction and Analogue Formulae for Type 2 Diabetes. [Dissertation]. Guangzhou (Guangdong): Guangzhou University of Chinese Medicine. (This is a retrospective study.)

[3] Huang, K.Y., and Li, S.M. (2014). Study on Treatment of Shaoyang Type 2 Diabetes with Chaihu Decoction and Analogue Formulae. Inner Mongolia J Tradit Chin Med. 33(32), 1-3. doi: 10.16040/j.cnki.cn15-1101.2014.32.199.(This is a retrospective study.)

[4] Zhang, Y.N. (2016). Dachaihu Decoction in Treating 32 Cases of Type 2 Diabetes. J Health Guide. (51), 200. doi: 10.3969/j.issn.1006-6845.2016.51.192. (This is a retrospective study.)

[5] Shao, L.C. (2013). Study of Dachaihu Decoction Intervention on Insulin Sensitivity of Oral Drug Failure of Obese Patients with Diabetes Mellitus. China Prac Med. 8(20), 82-83. doi: 10.14163/j.cnki.11-5547/r.2013.20.100. (The literature does not mention randomization.)

[6] Shi, Y. (2019). To Observe the Clinical Effect of Modified Dachaihu Decoction in the Treatment of Obesity Type 2 Diabetes Mellitus. Home Med. (10), 143-144. (This literature was grouped according to the wishes of the investigators and was not a randomized controlled trial.)

**2) The study subjects were not patients with type 2 diabetes:**

[1] Shi, Y.J.Y., and Wang, P. (1990). Treatment of Hyperlipidemia Complicated with Diabetes by Chinese Prescription: Anti-hyperlipidemic Effect of Dachaihu Decoction. Foreign Med Sci: Tradit Chin Med. (6), 17-19.

[2] Hu, M.F. (2009). Clinical Study on 40 Cases of Impaired Glucose Tolerance Treated with Modified Dachaihu Decoction. Zhejiang J Tradit Chin Med. 44(5), 318-319.

[3] Xu, K., Mei, W.X., and Chen, T. (2009). Clinical Observation of Modified Dachaihu Decoction in Treating Impaired Glucose Tolerance. Hubei J TCM. 31(12), 57.

[4] Shi, J.B., Huang, B.Y., Liu, F., and Chen, H. (2017). Clinical Observation on Dachaihu Decotion in Treating Pre-diabetes with Syndrome of Heat Stagnation in Liver and Stomach. Chin Med Mod Dist Educ. 15(13), 72-74.

[5] Li, N.Y. (2018). Analysis of the Effect of Dachaihu Decoction in the Treatment of Patients with Prediabetes. Contemp Med Symp. 16(5), 207-208.

[6] Zhang, Y.Q., and Sun, C.L. (2020). Observation on the Curative Effect of Modified Dachaihu Decoction in Treating Heat Stagnation in Liver and Stomach Syndrome of Prediabetes Mellitus. Healthmust-ReadMag. (26), 80.

**3) Subjects with severe renal impairment or infection:**

[1] Shi, H.Y., and Zhu, X.Y. (2004). Observation on the Effect of Modified Dachaihu Decoction on 32 Cases of Stage IV Diabetic Nephropathy. J Tradit Chin Med 45(3), 195-196. doi: 10.13288/j.11-2166/r.2004.03.023.

**4) Interventions are not DCHD:**

[1] Wang, X.C. (2006). The Correlation between RBC-Insulin Receptor of Type 2 Diabetes Mellitus and Insulin Resistance and a Clinical Research of Treatment with Chinese Herbs. [Master's thesis]. Shijiazhuang(Hebei): Hebei Medical University.

[2] Li, J.W. (2011). Theoretical Exploration and Clinical Research on Therapy of Type 2 Diabetes Mellitus (T2DM) Based on the Method of Harmonizing Ascending and Descending of Essential QI. [Dissertation]. Guangzhou(Guangdong): Guangzhou University of Chinese Medicine.

**5) The control measure does not meet the standard:**

[1] Chen, L.P., and Liu, M. (2005). Observation on the Curative Effect of Modified Dachaihu Decoction in Treating 58 Cases of Type 2 Diabetes Mellitus Complicated with Hyperlipidemia. J New Chin Med. 37(11), 36-37. doi: 10.13457/j.cnki.jncm. 2005.11.017. (The control group was a lipid-lowering drug.)

[2] Yuan, H., Chen, L.Y., Fang, X.Y., Yin, K., Zhang, J., and Zhu, X.Y. (2019). Clinical Study of Dachaihu Decoction in Treating Newly Obese Type 2 Diabetes Mellitus. J Sichuan Tradit Chin Med. 37(10), 110-112. (Single-arm trial with no control group.)

[3] Wang, Z.F., Li, Y.X., Wu, R., Li, A.J., Zhang, Y., Shi, B., et al. (2018). Observation on the Effect of Insulin Glargine Combined with Dachaihu Decoction on Blood Sugar in Patients with Type 2 Diabetes Mellitus. J Pract Diabetol. 14(6), 65-66. (The types of hypoglycemic drugs in the control group and the experimental group were different.)

[4] Deng, X., and Wang, W.J. (2011). 39 Cases of Obesity Diabetes Treated by Dachaihu Decoction. Shaanxi J Tradit Chin Med. 32(9), 1171-1172. (The doses of hypoglycemic drugs in the experimental group were different from those in the control group.)

**6) Duplicate literature:**

[1] Wang, P. (1991). Anti-lipid Effect of Dachaihu Decoction. Chin Tradit Herb Drugs. 22(1), 46.

[2] Zhang, Y.N. (2015). Dachaihu Decoction in Treating 32 Cases of Type 2 Diabetes. J Health Guide: Med Res. (10), 200.

[3] Guan, S., and Zhao, C.F. (2013). Observation of Curative Effect of Resolving Dampness, Downbearing Turbidity and Invigorating Blood in Treating Type 2 Diabetes Complicated with Non-alcoholic Fatty Liver. Shaanxi J Tradit Chin Med. 34(4), 412-414.

**7) Lack of sufficient data results:**

[1] Tong, X. (1996). Effects of Jisheng Shenqi Pill and Dachaihu Decoction on Skin Microcirculation in Diabetic Patients. Foreign Med Sci: Tradit Chin Med. 18(1), 19.

[2] Yan, Y. (1996). Chaihu Jia Longgu Muli Decoction in Treating Diabetic Patients with Abnormal Plasma Lipoprotein and Glucose Metabolism: a Comparison with Da Chaihu Decoction. Foreign Med Sci: Tradit Chin Med. 18(3), 31-32.

[3] Mo, W., Zhou, H., Zhang, Y., and Lv, X. (2014). Observation on Curative Effect of Modified Dachaihu Decoction in Treating Diabetic Peripheral Neuropathy. Chin Manipulation Rehabil Med. 5(5), 114-115.

[4] Yuan, H., Zhu, X.Y., Chen, L.Y., Zhang, J., and Yin, K. (2016). Clinical Effect of Modified Dachaihu Decoction Combined with Metformin Hydrochloride Enteric-Coated Capsules in the Treatment of Patients with Type 2 Diabetes Mellitus. Med Inf. 29(16), 412-412. doi: 10.3969/j.issn.1006-1959.2016.16.408.

**8) There are obvious errors:**

[1] Li, J.J., and Zhou, L.H. (2020). Discussion on the Application of Dachaihu Decoction in Diabetes with Heat Stagnation in Liver and Stomach Syndrome. Diabetes World. 17(4), 66.

**Final Included References**

[1] Liu, H.F. (2002). Clinical and Experimental Study on Therapeutic Effect of Jiaweidachaihu Granula(JG) in Improving Insulin Resistance. [Dissertation]. Beijing: Beijing University of Chinese Medicine.

[2] Shen, L.X., and Wu, Q.P. (2007). Study on Improvement of Islet β Cell Function in Patients with Type 2 Diabetes by Integrative Chinese and Western Medicine. Chongqing Med. 36(23), 2420-2421.

[3] Cui, H.Y., and Chen, Y.L. (2015). Clinical Observation on the Treatment of Type 2 Diabetes with Modified Dachaihu Decoction. Hebei J TCM. 37(8), 1195-1197. doi: 10.3969/j.issn.1002-2619.2015.08.026.

[4] Zhao, C.F., Li, H., Cheng, L., and Guan, S. (2016). Clinical Observation on the Treatment of Type 2 Diabetes Mellitus with Hyperlipidemia by Clearing Liver, Downbearing Turbidity and Resolving Dampness. World Chin Med. 11(2), 253-255. doi: 10.3969/j.issn.1673-7202.2016.02.018.

[5] Li, L.P., Zhang, H.Q., Gao, Y.H., Chen, H.W., Wang, J.Y., and Liu, P.S. (2018). Clinical Observation on 102 Cases of Obesity Type 2 Diabetes Treated with Dachaihu Decoction. Chin Med Mod Dist Educ. 16(24), 43-45. doi: 10.3969/j.issn.1672-2779.2018.24.019.

[6] Zhang, W.H., Li, Q.Y., Yang, C.W., Zhou, Y.R., Guo, Z.H., Wang, T.T., et al. (2018). Effect of Dachaihu Decoction Combined with Liraglutide on Insulin Resistance, Beta-Cell Function and Low Inflammatory Response in Obese Patients with Type 2 Diabetes Mellitus. Mod J Integr Tradit Chin West Med. 27(1), 23-26+30. doi: 10.3969/j.issn.1008-8849.2018.01.006.

[7] Zhang, X.H. (2018). Clinical Observation of Dachaihu Decoction in Treating Type 2 Diabetes Mellitus. Yunnan J Tradit Chin Med. 39(7), 44-45. doi: 10.16254/j.cnki.53-1120/r.2018.07.018.

[8] Gao, L.J. (2019). Effects of Modified Dachaihu Decoction on Insulin Resistance, β -Cell Function and Low Inflammatory Response in the Adjuvant Treatment of Obesity Type 2 Diabetes Mellitus. China's Naturopathy. 27(14), 61-62. doi: 10.19621/j.cnki.11-3555/r.2019.1431.

[9] Li, S.W. (2019). Effects of Modified Dachaihu Decoction Combined with Liraglutide on Insulin Resistance, β-Cell Function and Low Inflammatory Response in Obese Type 2 Diabetes Mellitus. Harbin Med J. 39(2), 185-186.

[10] Zhang, H.S., Liu, X.Q., Han, X., Zhang, W., and Xu, J.J. (2019). Effect of Dachaihu Decoction on Hypoglycemic Efficacy and Blood Glucose Fluctuation in Type 2 Diabetes Mellitus with Initial Liver-Stomach Depression-Heat Treated by Intensive Insulin. Hubei J TCM. 41(11), 7-9.

[11] Bao, W.P., Fan, Y.F., and Chu, X.Q. (2020). Efficiency Observation of Dachaihu Decoction and Exenatide on Newly Diagnosed Type 2 Diabetes Mellitus Complicated by Non-alcoholic Fatty Liver. Shanxi J TCM. 36(2), 27-29.

[12] Chang, L.L. (2020). Effect of Insulin Glargine Combined with Dachaihu Decoction in the Treatment of T2DM Patients and Its Effect on Blood Sugar Level. J Pract Diabetol. 16(5), 71-72.

[13] Ji, J.L., and Che, Z.Y. (2020). 20 Cases of Type 2 Diabetes Treated with Modified Dachaihu Decoction Combined with Metformin. TCM Res. 33(9), 18-21. doi: 10.3969/j.issn.1001-6910.2020.09.06.

[14] Wang, Z.H. (2020). Clinical Efficacy of Modified Dachaihu Decoction Combined with Western Medicine in the Treatment of Obese Type 2 Diabetes Mellitus and Its Effect on Insulin Resistance in Patients. Henan Tradit Chin Med. 40(1), 56-59. doi: 10.16367/j.issn.1003-5028.2020.01.0015.

[15] Duan, J. (2021). Clinical Effect Analysis of Modified Dachaihu Decoction in the Treatment of Type 2 Diabetes Mellitus. Diabetes New World. 24(2), 60-62. doi: 10.16658/j.cnki.1672-4062.2021.02.060.

[16] Zhang, Q.J., Wu, R., Wang, Z.F., Qin, L., Guo, F.N., An, R., et al. (2021). Clinical Study of Modified Dachaihu Decoction on Type 2 Diabetes Mellitus Complicated with Hyperuricemia. J Clin Exp Med. 20(5), 478-482. doi: 10.3969/j.issn.1671-4695.2021.05.008.

[17] Zou, Y.L., Zhang, W.B., He, Y.Y., and Li, H.X. (2021). Effects of Dachaihu Decoction on Blood Glucose and Blood Lipid Levels in Patients with Type 2 Diabetes Mellitus Complicated by Obesity. Mod Med Health Res. 5(9), 18-20.

# Supplementary Material S5. Meta-regression analysis of HbA1c and FBG

5.1 The results of meta-regression analysis of HbA1c on average age.

5.2 The results of meta-regression analysis of HbA1c on sample size.

5.3 The results of meta-regression analysis of HbA1c on publication year.

5.4 The results of meta-regression analysis of FBG on average age.

5.5 The results of meta-regression analysis of FBG on sample size.

5.6 The results of meta-regression analysis of FBG on publication year.

# Supplementary Material S6. Subgroup analysis of HbA1c for DCHD combined with conventional treatment vs. conventional treatment

**
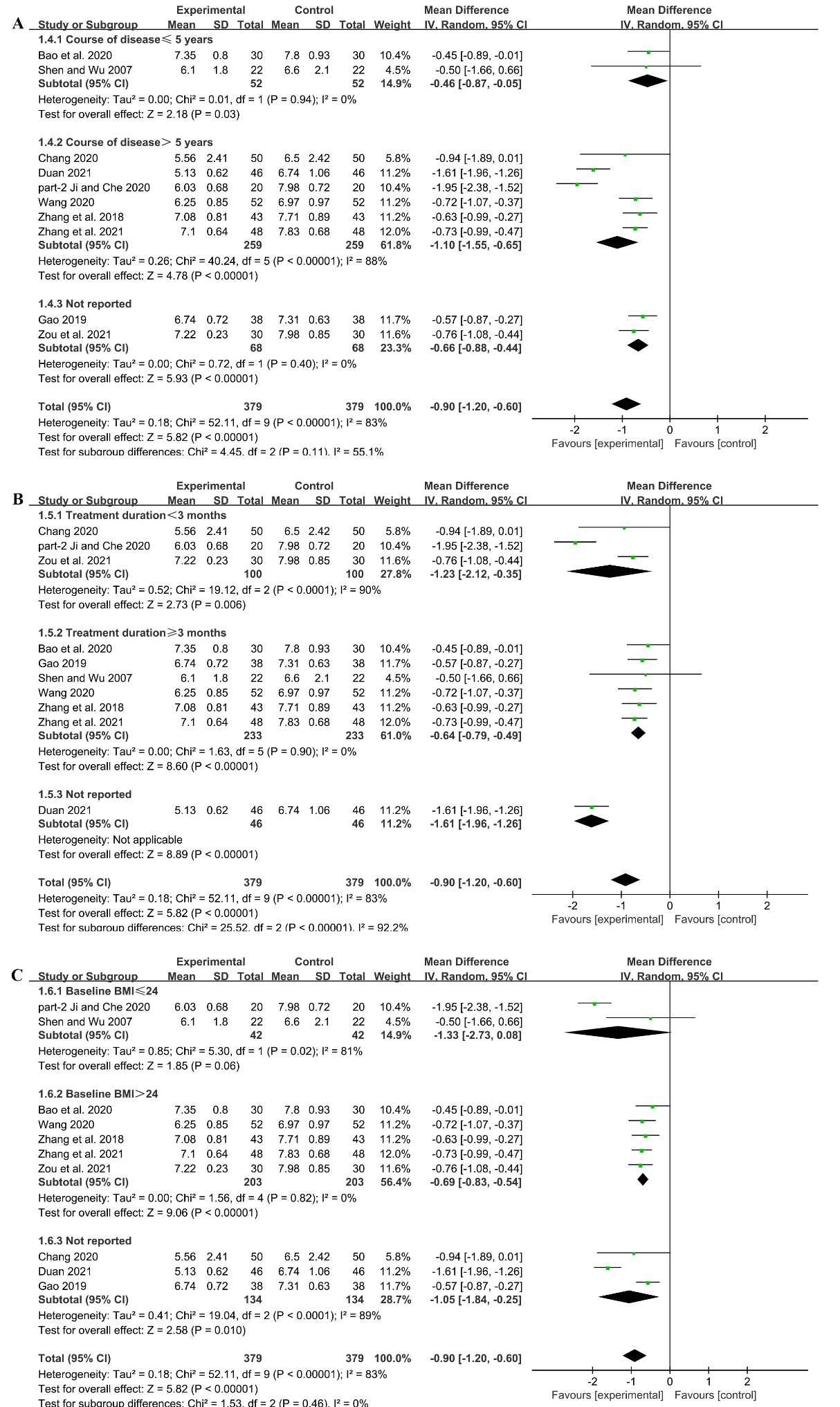
**

# Supplementary Material S7. Sensitivity analysis

7.1 The results of sensitivity analysis of HbA1c for DCHD combined with conventional treatment vs. conventional treatment.

7.2 The results of sensitivity analysis of HbA1c for DCHD vs. conventional treatment.

7.3 The results of sensitivity analysis of HbA1c for DCHD vs. conventional treatment after excluding Zhao et al. 2016.


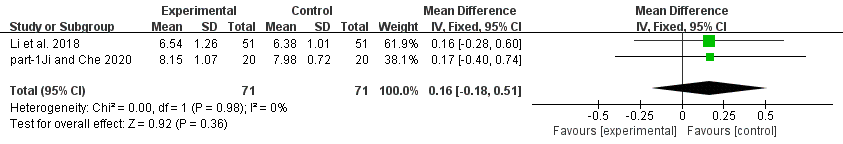


7.4 The results of sensitivity analysis of FBG for DCHD combined with conventional treatment vs. conventional treatment.

7.5 The results of sensitivity analysis of FBG for DCHD vs. conventional treatment.

7.6 The results of sensitivity analysis of FBG for DCHD vs. conventional treatment after excluding Zhao et al. 2016.


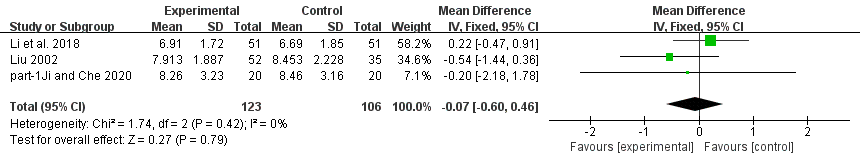


7.7 The results of sensitivity analysis of 2hPG for DCHD combined with conventional treatment vs. conventional treatment.

7.8 The results of sensitivity analysis of 2hPG for DCHD vs. conventional treatment.

7.9 The results of sensitivity analysis of 2hPG for DCHD vs. conventional treatment after excluding Zhao et al. 2016.


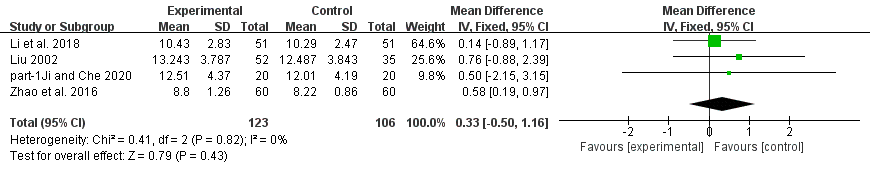


7.10 The results of sensitivity analysis of TC for DCHD combined with conventional treatment vs. conventional treatment.

7.11 The results of sensitivity analysis of TC for DCHD vs. conventional treatment after changing the effect model.


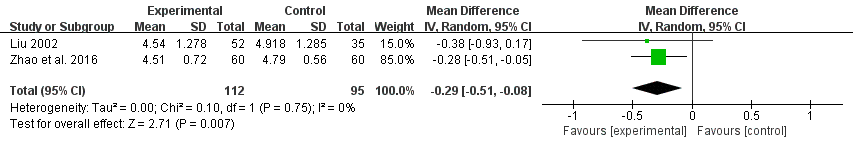


7.12 The results of sensitivity analysis of TG for DCHD combined with conventional treatment vs. conventional treatment.

7.13 The results of sensitivity analysis of TG for DCHD vs. conventional treatment after changing the effect model.


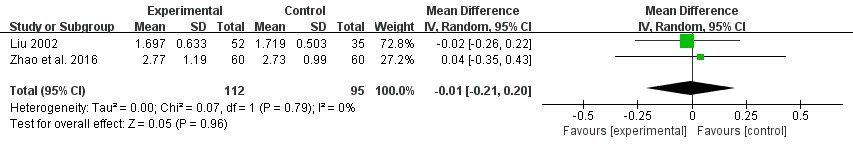


7.14 The results of sensitivity analysis of HDL-C for DCHD vs. conventional treatment after changing the effect model.


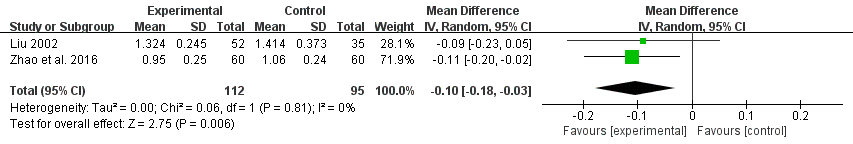


7.15 The results of sensitivity analysis of LDL-C for DCHD combined with conventional treatment vs. conventional treatment after changing the effect model.


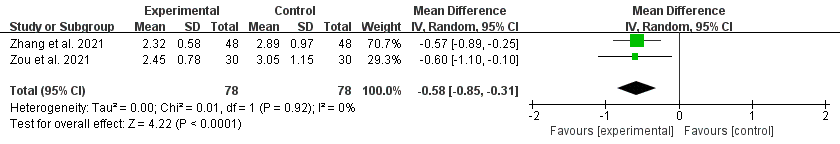


7.16 The results of sensitivity analysis of LDL-C for DCHD vs. conventional treatment after changing the effect model.


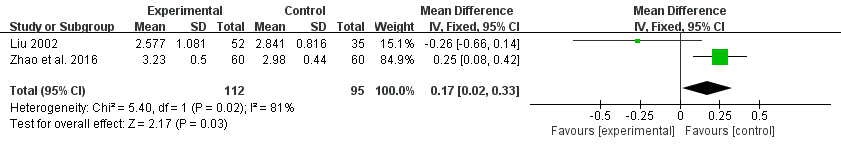


7.17 The results of sensitivity analysis of HOMA-IR for DCHD combined with conventional treatment vs. conventional treatment.

7.18 The results of sensitivity analysis of HOMA-β for DCHD combined with conventional treatment vs. conventional treatment.

7.19 The results of sensitivity analysis of BMI for DCHD combined with conventional treatment vs. conventional treatment.

7.20 The results of sensitivity analysis of BMI for DCHD vs. conventional treatment after changing the effect model.


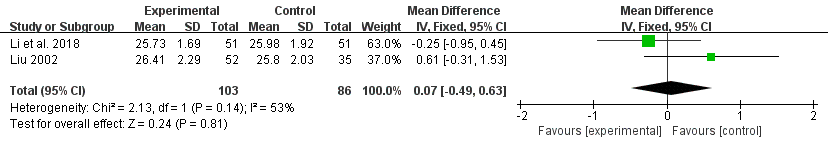


# Supplementary Material S8. Subgroup analysis of HbA1c for DCHD vs. conventional treatment


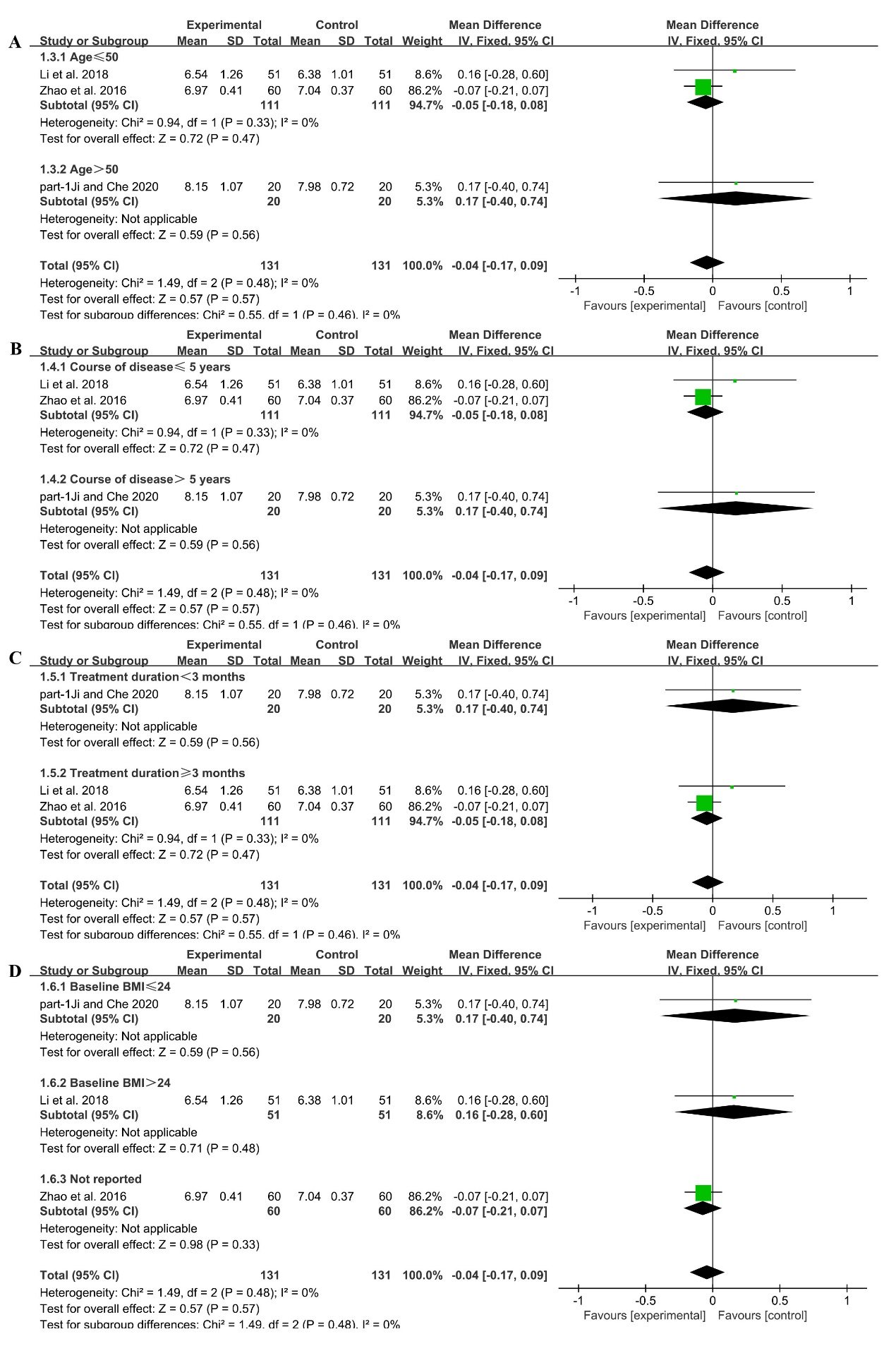


# Supplementary Material S9. Subgroup analysis of FBG for DCHD combined with conventional treatment vs. conventional treatment


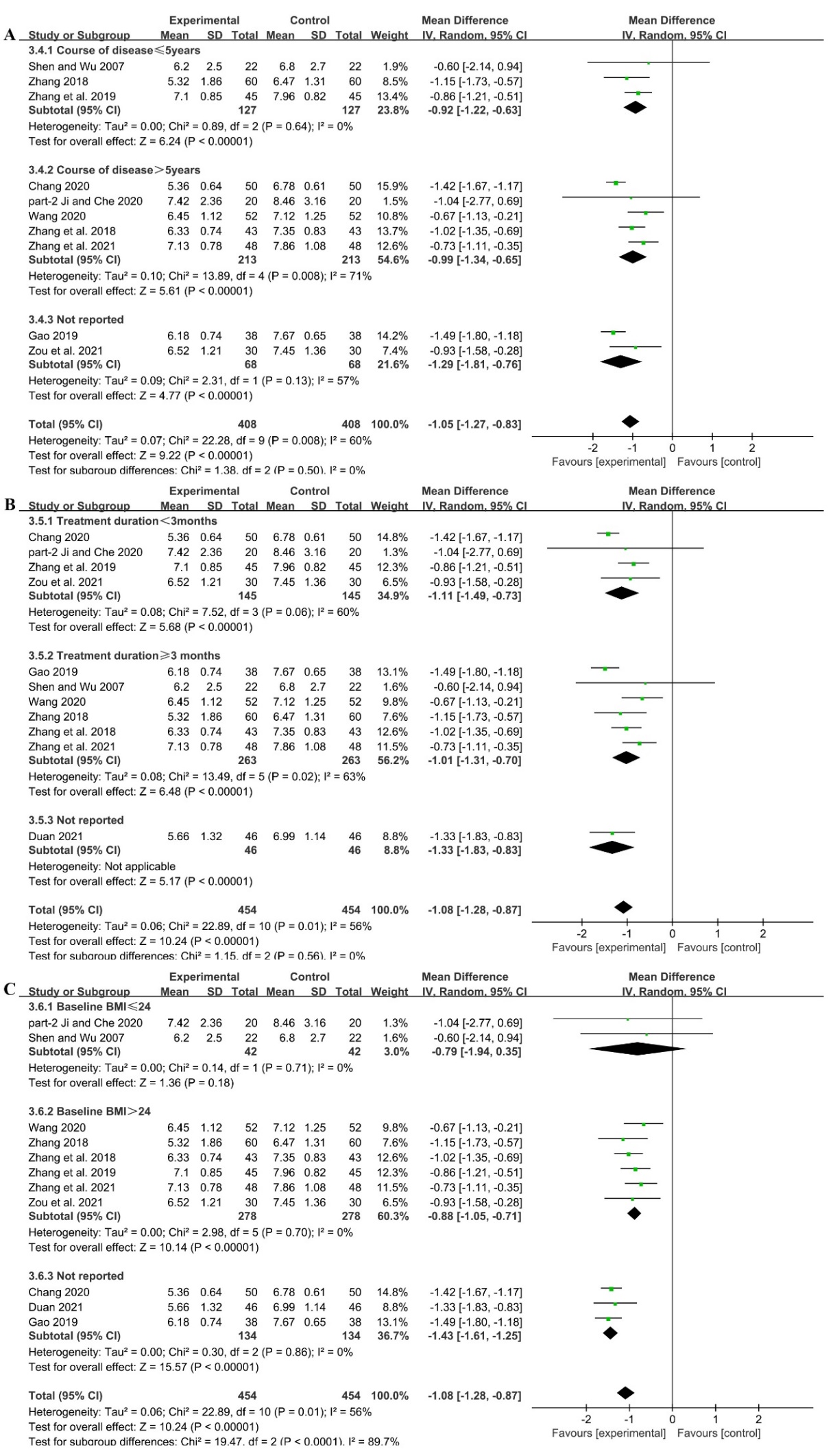


# Supplementary Material S10. Subgroup analysis of FBG for DCHD vs. conventional treatment

**
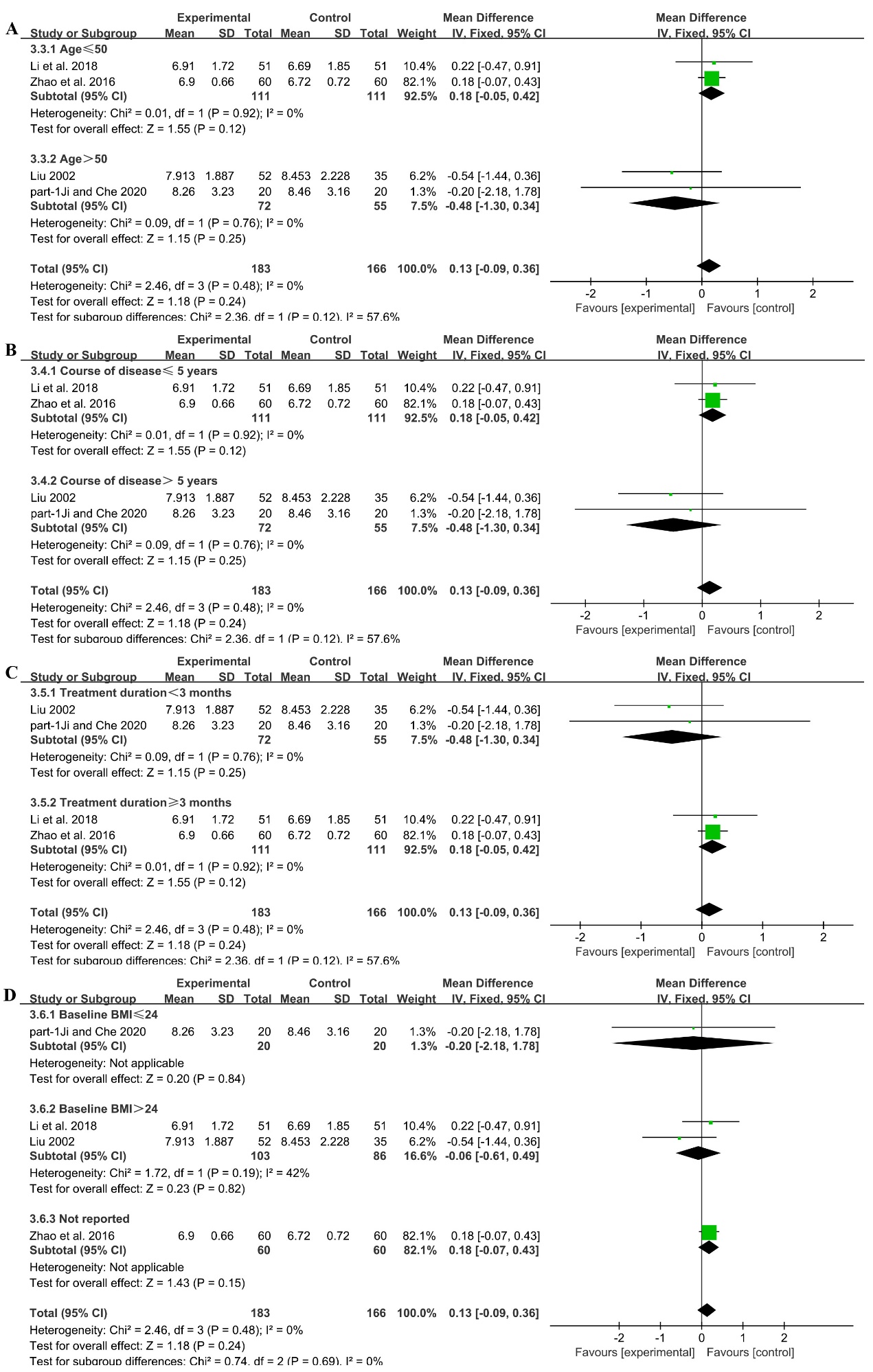
**

# Supplementary Material S11. Subgroup analysis of 2hPG for DCHD combined with conventional treatment vs. conventional treatment


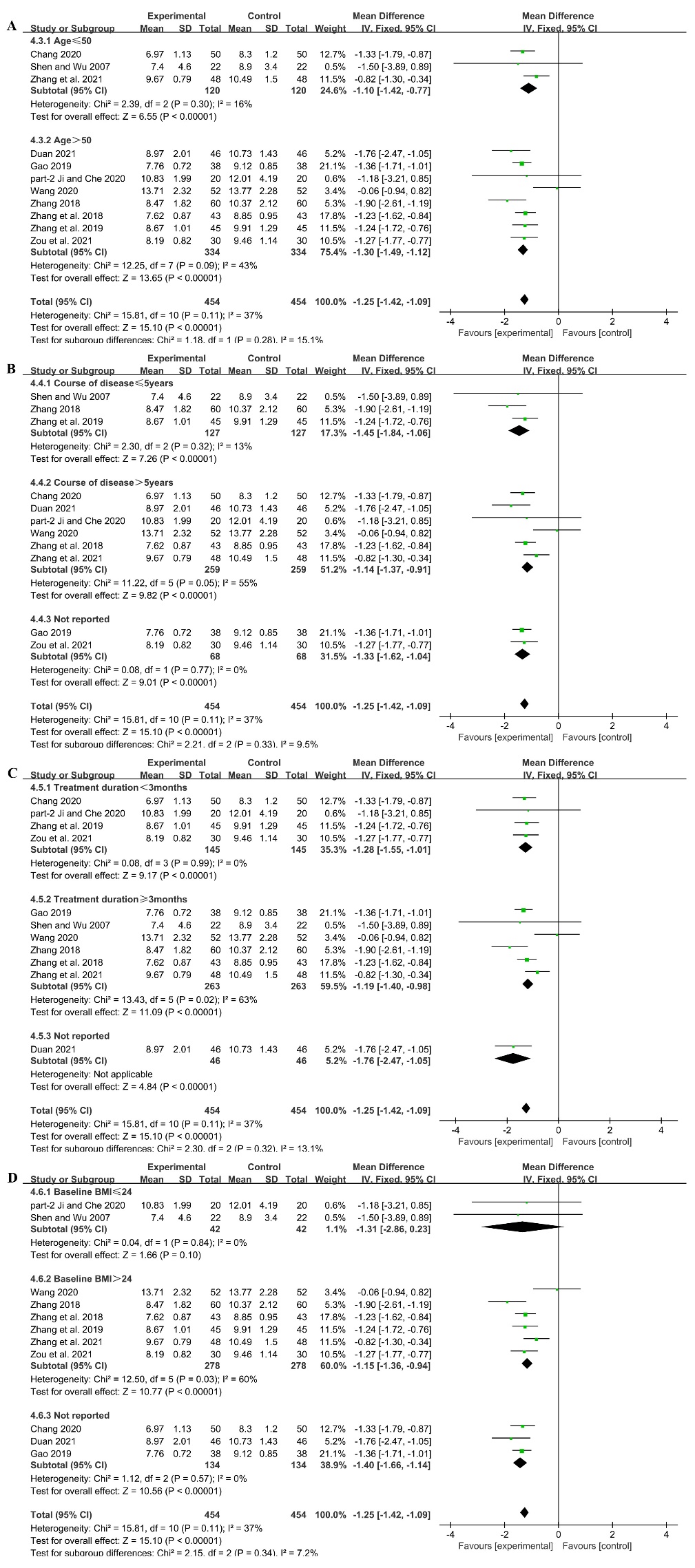


# Supplementary Material S12. Subgroup analysis of 2hPG for DCHD vs. conventional treatment

**
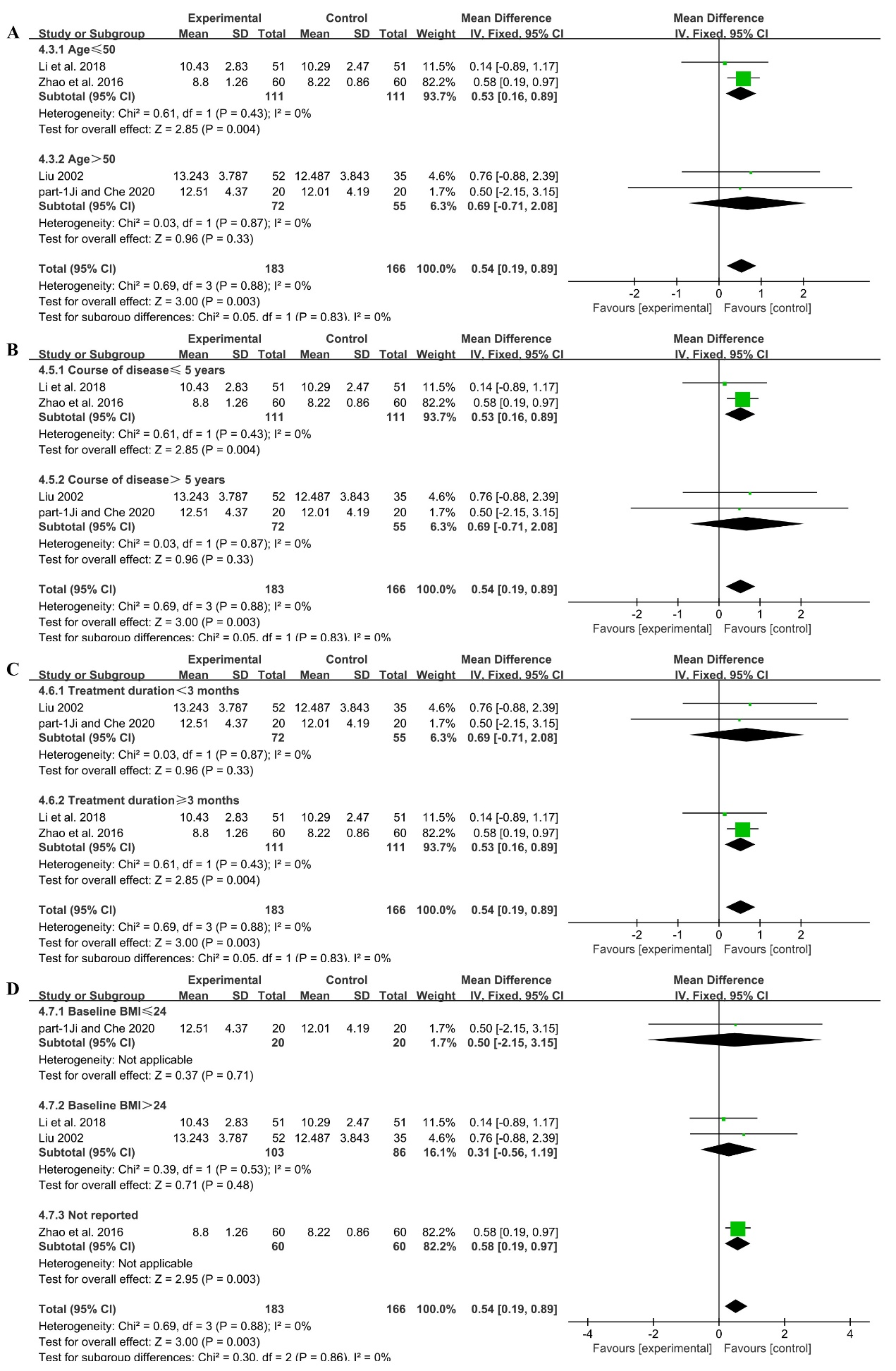
**

# Supplementary Material S13. Subgroup analysis of TC for DCHD combined with conventional treatment vs. conventional treatment


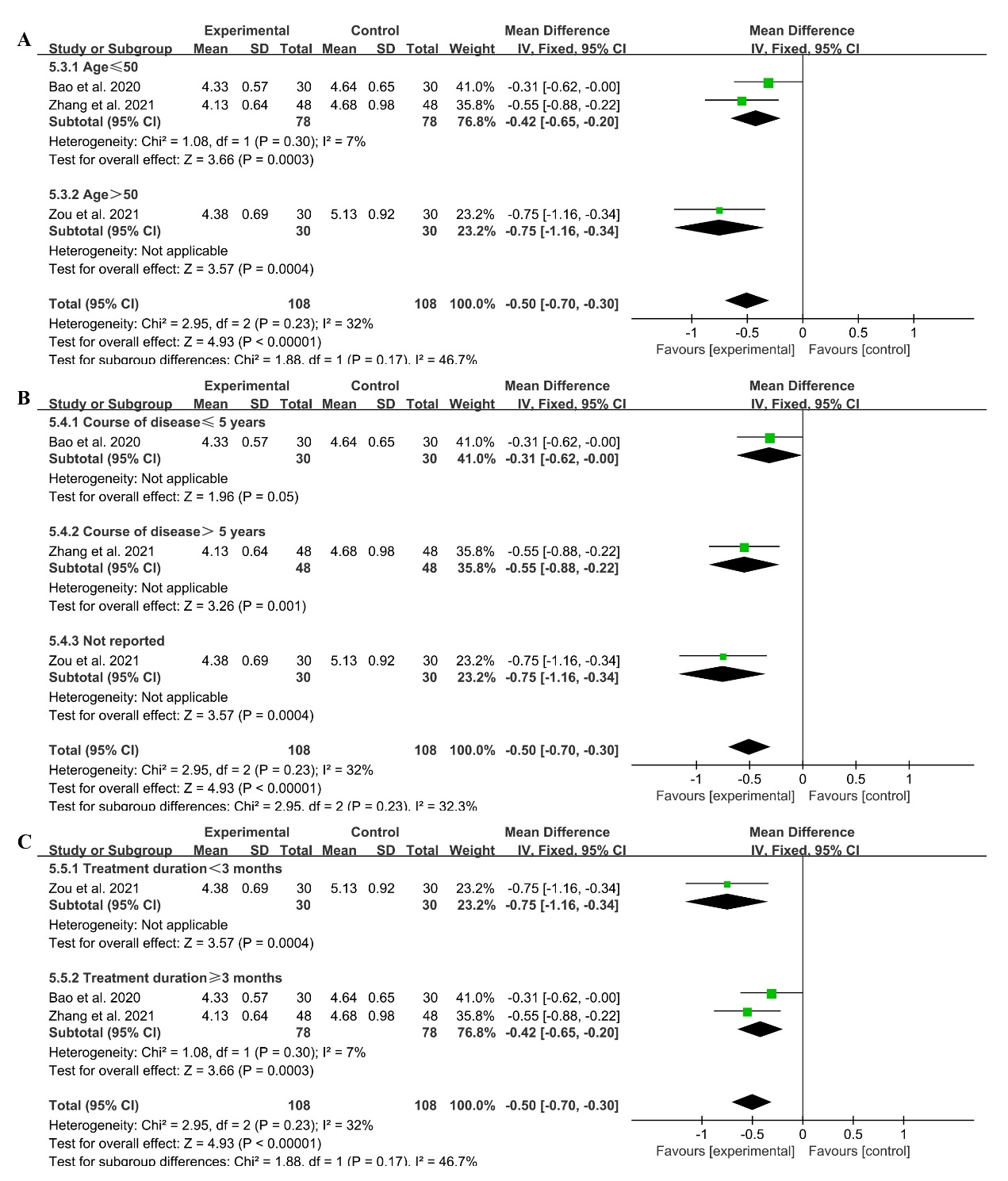


Supplementary Material S14. Subgroup analysis of TG for DCHD combined with conventional treatment vs. conventional treatment

**
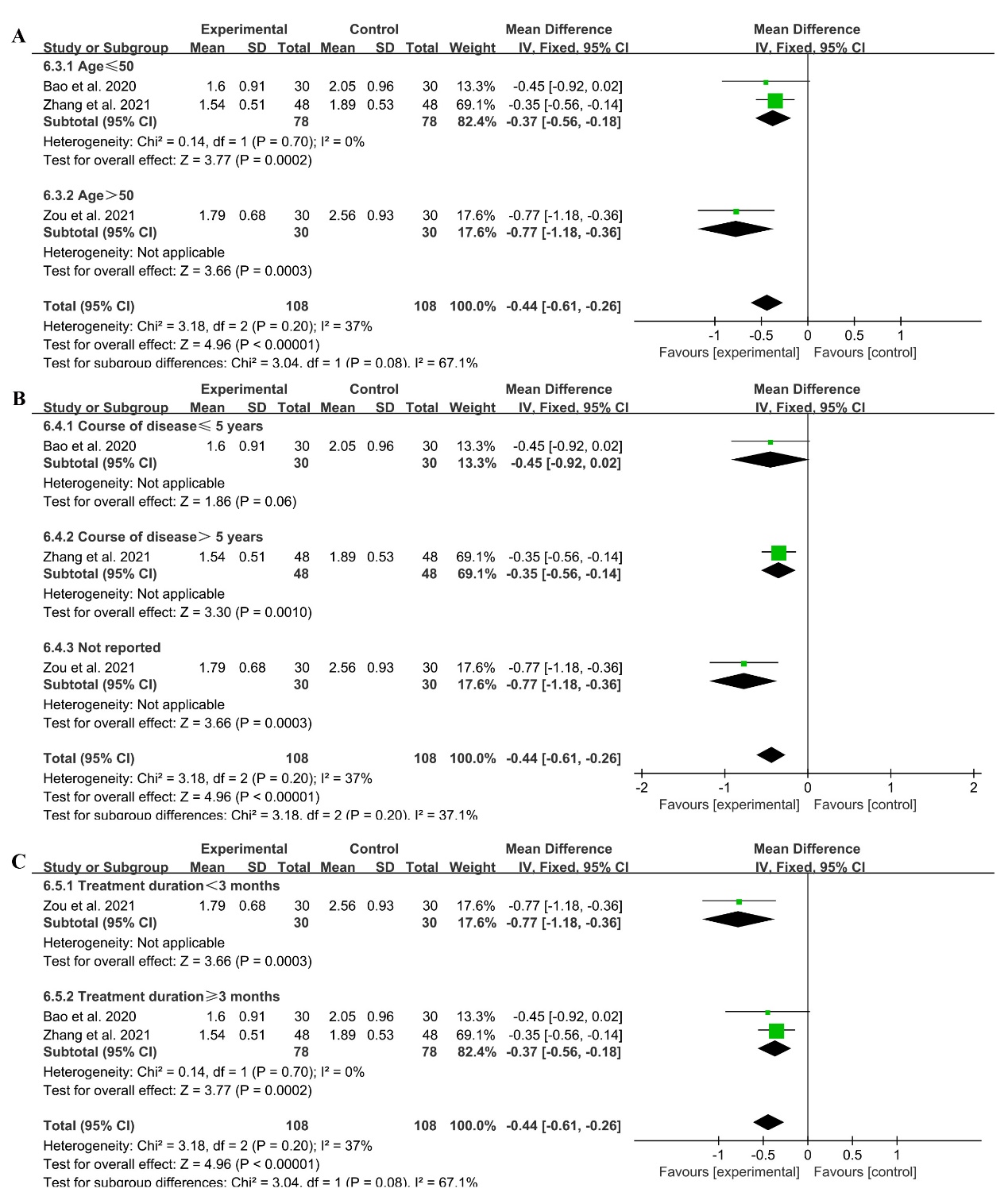
**

Supplementary Material S15. Subgroup analysis of HOMA-IR for DCHD combined with conventional treatment vs. conventional treatment

**
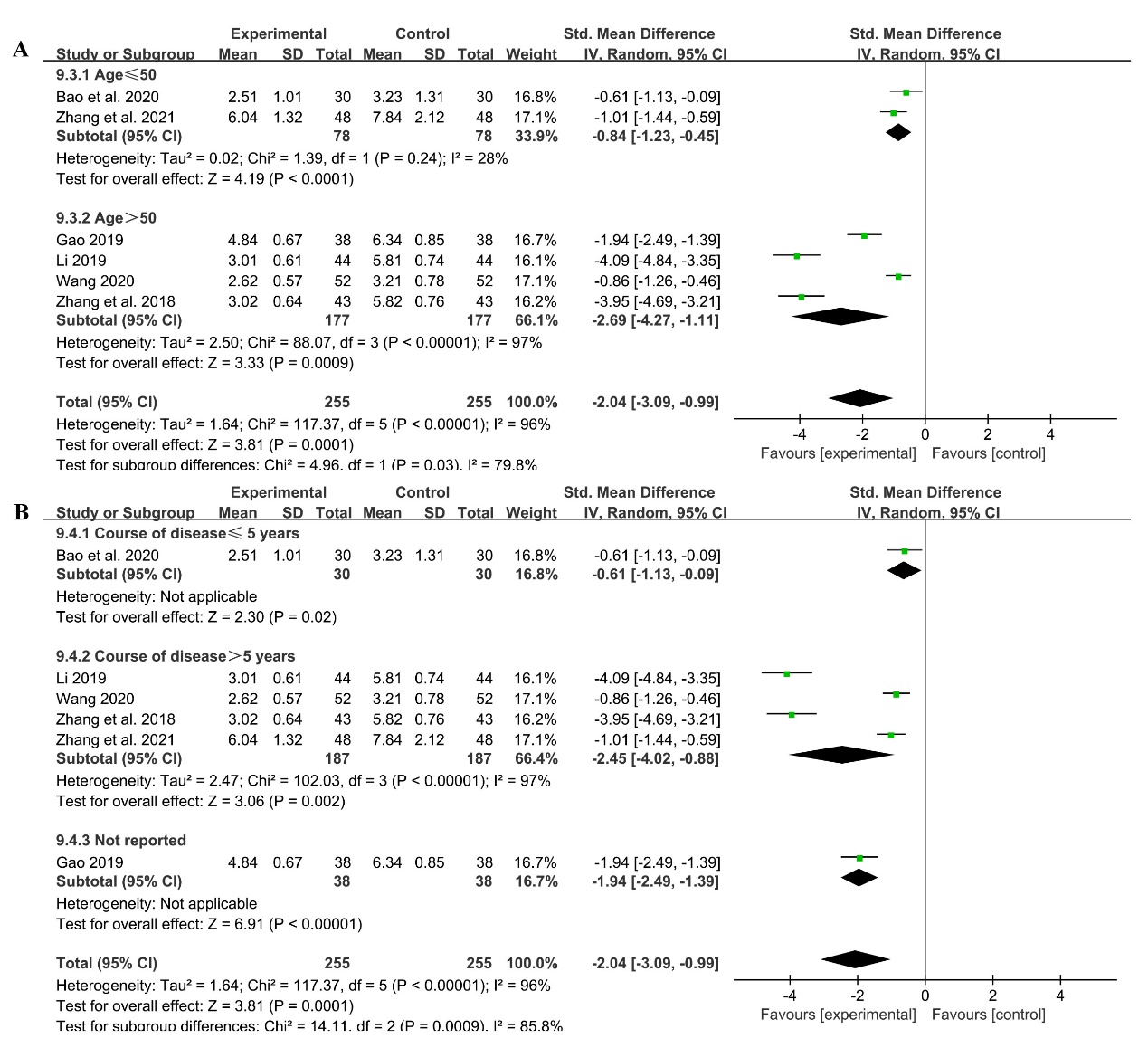
**

# Supplementary Material S16. Subgroup analysis of BMI for DCHD combined with conventional treatment vs. conventional treatment

**
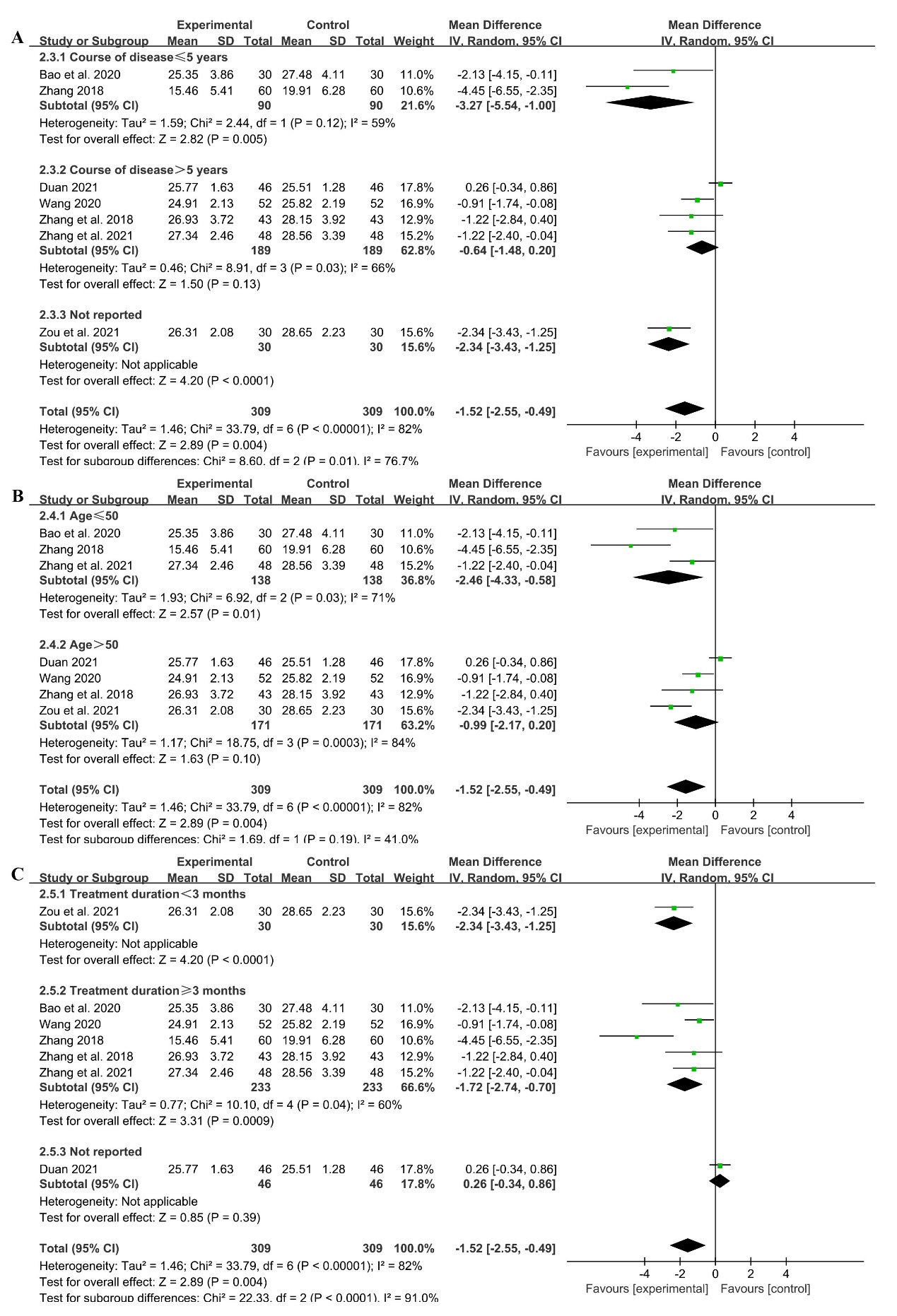
**

# Supplementary Material S17. Egger’s test of HBA1c, FBG and 2hPG

17.1 Egger’s test of HBA1c

17.2 Egger’s test of FBG

17.3 Egger’s test of 2hPG

# Supplementary Material S18. Assessment of evidence quality for each outcome

**DCHD combined with Conventional treatment vs. Conventional treatment**

| **Quality assessment** | | | | | | | **No of patients** | | **Effect** | | **Quality** | **Importance** |  |
| --- | --- | --- | --- | --- | --- | --- | --- | --- | --- | --- | --- | --- | --- |
|  |  |  |  |  |  |  |  |  |  |  |  |  |  |
| **No of studies** | **Design** | **Risk of bias** | **Inconsistency** | **Indirectness** | **Imprecision** | **Other considerations** | **HBA1C** | **Control** | **Relative (95% CI)** | **Absolute** |  |  |  |
| **HbA1c (Better indicated by lower values)** | | | | | | | | | | | | |  |
| 10 | randomised trials | serious^1^ | serious^2^ | no serious indirectness | no serious imprecision | none | 379 | 379 | - | MD 0.9 lower (1.2 to 0.6 lower) | ⊕⊕OO LOW | CRITICAL |  |
| **BMI (Better indicated by lower values)** | | | | | | | | | | | | |  |
| 7 | randomised trials | serious^1^ | serious^2^ | no serious indirectness | no serious imprecision | none | 309 | 309 | - | MD 1.52 lower (2.55 to 0.49 lower) | ⊕⊕OO LOW | IMPORTANT |  |
| **FBG (Better indicated by lower values)** | | | | | | | | | | | | |  |
| 11 | randomised trials | serious^1^ | serious^2^ | no serious indirectness | no serious imprecision | none | 454 | 454 | - | MD 1.14 lower (1.26 to 1.01 lower) | ⊕⊕OO LOW | CRITICAL |  |
| **2hPG (Better indicated by lower values)** | | | | | | | | | | | | |  |
| 11 | randomised trials | serious^1^ | no serious inconsistency | no serious indirectness | no serious imprecision | none | 454 | 454 | - | MD 1.25 lower (1.42 to 1.09 lower) | ⊕⊕⊕O MODERATE | CRITICAL |  |
| **TC (Better indicated by lower values)** | | | | | | | | | | | | |  |
| 4 | randomised trials | serious^1^ | serious^2^ | no serious indirectness | no serious imprecision | none | 168 | 168 | - | MD 0.62 lower (0.88 to 0.36 lower) | ⊕⊕OO LOW | IMPORTANT |  |
| **TG (Better indicated by lower values)** | | | | | | | | | | | | |  |
| 4 | randomised trials | serious^1^ | serious^2^ | no serious indirectness | serious^3^ | none | 168 | 168 | - | MD 0.76 lower (1.31 to 0.2 lower) | ⊕OOO VERY LOW | IMPORTANT |  |
| **LDL-C (Better indicated by lower values)** | | | | | | | | | | | | |  |
| 2 | randomised trials | serious^1^ | no serious inconsistency | no serious indirectness | serious^2^ | none | 78 | 78 | - | MD 0.58 lower (0.85 to 0.31 lower) | ⊕⊕OO LOW | IMPORTANT |  |
| **HOMA-IR (Better indicated by lower values)** | | | | | | | | | | | | |  |
| 6 | randomised trials | serious^1^ | serious^2^ | no serious indirectness | serious^3^ | none | 255 | 255 | - | SMD 2.04 lower (3.09 to 0.99 lower) | ⊕OOO VERY LOW | IMPORTANT |  |
| **HOMA-β (Better indicated by higher values)** | | | | | | | | | | | | |  |
| 4 | randomised trials | serious^1^ | no serious inconsistency | no serious indirectness | no serious imprecision | none | 177 | 177 | - | SMD 2.48 higher (2.2 to 2.76 higher) | ⊕⊕⊕O MODERATE | IMPORTANT |  |

^1^ Poor methodological quality, such as not using blinding or not reporting in detail the specific methods for generating random sequences and allocation concealment.
^2^ Large differences in the effect size of each study point or small overlap of confidence intervals, or large heterogeneity.

^3^ Small sample size or wide confidence interval.

**DCHD vs. Conventional treatment**

| **Quality assessment** | | | | | | | **No of patients** | | **Effect** | | **Quality** | **Importance** |  |
| --- | --- | --- | --- | --- | --- | --- | --- | --- | --- | --- | --- | --- | --- |
|  |  |  |  |  |  |  |  |  |  |  |  |  |  |
| **No of studies** | **Design** | **Risk of bias** | **Inconsistency** | **Indirectness** | **Imprecision** | **Other considerations** | **BMI** | **Control** | **Relative (95% CI)** | **Absolute** |  |  |  |
| **HbA1c (Better indicated by lower values)** | | | | | | | | | | | | |  |
| 3 | randomised trials | serious^1^ | no serious inconsistency | no serious indirectness | serious^2^ | none | 131 | 131 | - | MD 0.04 lower (0.17 lower to 0.09 higher) | ⊕⊕OO LOW | CRITICAL |  |
| **BMI (Better indicated by lower values)** | | | | | | | | | | | | |  |
| 2 | randomised trials | serious^1^ | serious^2^ | no serious indirectness | serious^3^ | none | 103 | 86 | - | MD 0.07 higher (0.49 lower to 0.63 higher) | ⊕OOO VERY LOW | IMPORTANT |  |
| **FBG (Better indicated by lower values)** | | | | | | | | | | | | |  |
| 4 | randomised trials | serious^1^ | no serious inconsistency | no serious indirectness | no serious imprecision | none | 183 | 166 | - | MD 0.13 higher (0.09 lower to 0.36 higher) | ⊕⊕⊕O MODERATE | CRITICAL |  |
| **2hPG (Better indicated by lower values)** | | | | | | | | | | | | |  |
| 4 | randomised trials | serious^1^ | serious^2^ | no serious indirectness | no serious imprecision | none | 183 | 166 | - | MD 0.54 higher (0.19 to 0.89 higher) | ⊕⊕OO LOW | CRITICAL |  |
| **TC (Better indicated by lower values)** | | | | | | | | | | | | |  |
| 2 | randomised trials | serious^1^ | serious^2^ | no serious indirectness | serious^3^ | none | 112 | 95 | - | MD 0.29 lower (0.51 to 0.08 lower) | ⊕OOO VERY LOW | IMPORTANT |  |
| **TG (Better indicated by lower values)** | | | | | | | | | | | | |  |
| 2 | randomised trials | serious^1^ | no serious inconsistency | no serious indirectness | serious^2^ | none | 112 | 95 | - | MD 0.01 lower (0.21 lower to 0.2 higher) | ⊕⊕OO LOW | IMPORTANT |  |
| **HDL-C (Better indicated by higher values)** | | | | | | | | | | | | |  |
| 2 | randomised trials | serious^1^ | serious^2^ | no serious indirectness | serious^3^ | none | 112 | 95 | - | MD 0.1 lower (0.18 to 0.03 lower) | ⊕OOO VERY LOW | IMPORTANT |  |
| **LDL-C (Better indicated by lower values)** | | | | | | | | | | | | |  |
| 2 | randomised trials | serious^1^ | serious^2^ | no serious indirectness | serious^3^ | none | 112 | 95 | - | MD 0.17 higher (0.02 to 0.33 higher) | ⊕OOO VERY LOW | IMPORTANT |  |
| **HOMA-IR (Better indicated by lower values)** | | | | | | | | | | | | |  |
| 1 | randomised trials | serious^1^ | no serious inconsistency | no serious indirectness | serious^2^ | none | 60 | 60 | - | MD 0.2 lower (0.75 lower to 0.35 higher) | ⊕⊕OO LOW | IMPORTANT |  |

^1^ Poor methodological quality, such as not using blinding or not reporting in detail the specific methods for generating random sequences and allocation concealment.
^2^ Large differences in the effect size of each study point or small overlap of confidence intervals, or large heterogeneity.
^3^ Small sample size or wide confidence interval.

**DCHD vs. Placebo**

| **Quality assessment** | | | | | | | **No of patients** | | **Effect** | | **Quality** | **Importance** |  |
| --- | --- | --- | --- | --- | --- | --- | --- | --- | --- | --- | --- | --- | --- |
|  |  |  |  |  |  |  |  |  |  |  |  |  |  |
| **No of studies** | **Design** | **Risk of bias** | **Inconsistency** | **Indirectness** | **Imprecision** | **Other considerations** | **FBG** | **Control** | **Relative (95% CI)** | **Absolute** |  |  |  |
| **HbA1c (Better indicated by lower values)** | | | | | | | | | | | | |  |
| 1 | randomised trials | serious^1^ | no serious inconsistency | no serious indirectness | serious^2^ | none | 60 | 60 | - | MD 0.35 lower (0.68 to 0.02 lower) | ⊕⊕OO LOW | CRITICAL |  |
| **FBG (Better indicated by lower values)** | | | | | | | | | | | | |  |
| 1 | randomised trials | serious^1^ | no serious inconsistency | no serious indirectness | serious^2^ | none | 60 | 60 | - | MD 0.91 lower (1.29 to 0.53 lower) | ⊕⊕OO LOW | CRITICAL |  |
| **2hPG (Better indicated by lower values)** | | | | | | | | | | | | |  |
| 1 | randomised trials | serious^1^ | no serious inconsistency | no serious indirectness | serious^2^ | none | 60 | 60 | - | MD 0.53 lower (1.11 lower to 0.05 higher) | ⊕⊕OO LOW | CRITICAL |  |

^1^ Poor methodological quality, such as not using blinding or not reporting in detail the specific methods for generating random sequences and allocation concealment.
^2^ Large differences in the effect size of each study point or small overlap of confidence intervals, or large heterogeneity.
^3^ Small sample size or wide confidence interval.
